# Unveiling an indole alkaloid diketopiperazine biosynthetic pathway that features a unique stereoisomerase and multifunctional methyltransferase

**DOI:** 10.1038/s41467-023-38168-3

**Published:** 2023-05-03

**Authors:** Garrett Deletti, Sajan D. Green, Caleb Weber, Kristen N. Patterson, Swapnil S. Joshi, Tushar M. Khopade, Mathew Coban, James Veek-Wilson, Thomas R. Caulfield, Rajesh Viswanathan, Amy L. Lane

**Affiliations:** 1 Department of Chemistry & Biochemistry, University of North Florida, Jacksonville, FL 32224 USA; 2 Departments of Chemistry & Biology, Indian Institute of Science Education and Research Tirupati, Tirupati, Andhra Pradesh India; 3 Department of Cancer Biology, Mayo Clinic, Jacksonville, FL 32224 USA; 4 Department of Neuroscience, Mayo Clinic, Jacksonville, FL 32224 USA; 5 Present Address: Department of Chemistry, Emory University, Atlanta, GA 30322 USA

**Keywords:** Isomerases, Transferases, Natural product synthesis, Biosynthesis, Isomerases

## Abstract

The 2,5-diketopiperazines are a prominent class of bioactive molecules. The nocardioazines are actinomycete natural products that feature a pyrroloindoline diketopiperazine scaffold composed of two D-tryptophan residues functionalized by *N*- and *C*-methylation, prenylation, and diannulation. Here we identify and characterize the nocardioazine B biosynthetic pathway from marine *Nocardiopsis* sp. CMB-M0232 by using heterologous biotransformations, in vitro biochemical assays, and macromolecular modeling. Assembly of the *cyclo*-L-Trp-L-Trp diketopiperazine precursor is catalyzed by a cyclodipeptide synthase. A separate genomic locus encodes tailoring of this precursor and includes an aspartate/glutamate racemase homolog as an unusual D/L isomerase acting upon diketopiperazine substrates, a phytoene synthase-like prenyltransferase as the catalyst of indole alkaloid diketopiperazine prenylation, and a rare dual function methyltransferase as the catalyst of both *N*- and *C*-methylation as the final steps of nocardioazine B biosynthesis. The biosynthetic paradigms revealed herein showcase Nature’s molecular ingenuity and lay the foundation for diketopiperazine diversification via biocatalytic approaches.

## Abstract (web summary)

Diketopiperazine (DKP) natural products have diverse structures and biological functions. Here, the authors elucidate the biosynthetic pathway for indole alkaloid DKP nocardioazine B which includes DKP stereoisomerization by an unusual aspartate/glutamate racemase homolog and N- and C-methylation by a dual function methyltransferase.

## Introduction

Molecules with 2,5-diketopiperazine (DKP) groups are structurally diverse, widespread in nature, and offer myriad biological activities^1^. Nonribosomal peptide synthetases (NRPSs) were the only recognized biocatalysts for formation of the two peptide bonds of DKPs until the first cyclodipeptide synthases (CDPSs) were functionally characterized just over a decade ago^2^. CDPSs utilize two aminoacyl-tRNA substrates for the assembly of DKP skeletons, which are subsequently functionalized and diversified via tailoring reactions^3,4^. Nearly all of the >800 experimentally characterized or bioinformatics-predicted CDPSs are from bacterial genomic loci that also encode putative DKP tailoring enzymes^5^, yet these pathways remain strikingly understudied relative to those for other common biosynthetic classes. Exploration of a small sampling of these CDPS-containing pathways revealed unprecedented enzymology^6–14^, yielded structurally fascinating bioactive metabolites^15–17^, and enabled chemoenzymatic syntheses^18–20^.

DKP natural products constructed from tryptophan often feature pyrroloindoline moieties, which confer structural rigidity that contributes toward the biological activities of these molecules^21^. Pyrroloindoline DKPs include the lansais^22^, naseseazines^23^, guanitrypmycins^10,14^, nocardioazines (**1**–**2**)^24^, and numerous others. Functionalization at indoline *C*3 is common among these natural products, including bonds from *C*3 to alkyl, aromatic, and hydroxy groups^21^. Most pyrroloindoline DKPs feature L-tryptophan residues, with the inverted DKP configuration of nocardioazines A-B as a noteworthy exception^25,26^.

**Fig. 1:**
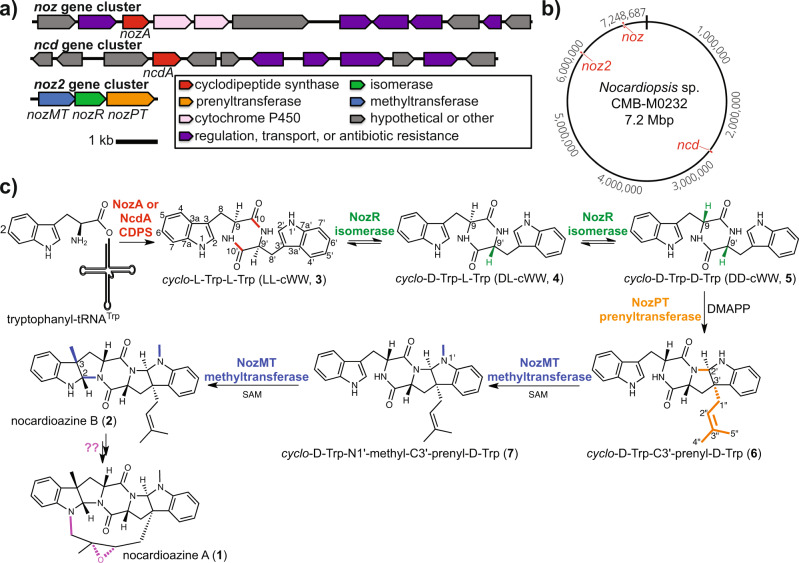
Overview of the nocardioazine B pathway from *Nocardiopsis* sp. CMB-M0232. **a** The *noz* and *ncd* gene clusters encode LL-cWW (**3**) production^27^, with CDPSs NozA and NcdA each catalyzing the assembly of **3** from Trp-tRNA^Trp^^30^. The *noz2* cluster encodes tailoring enzymes that transform **3** into nocardioazine B (**2**). **b** The *noz*, *ncd*, and *noz2* clusters are from different loci of the chromosome. **c** The biosynthetic pathway yielding **2** is proposed in the current study. (SAM *S*-adenosyl-L-methionine; DMAPP dimethylallyl pyrophosphate).

The nocardioazines (**1**–**2**) are *C*3’-prenylated, *N*1’- and *C*3-methylated, and diannulated pyrroloindoline alkaloid DKP natural products isolated from the marine actinomycete *Nocardiopsis* sp. CMB-M0232^24^. Nocardioazine A (**1**) reverses drug resistance in cancer cell lines by acting as a noncytotoxic inhibitor of P-glycoprotein^24^. Hypotheses about the biosynthetic pathway for *C*3’ prenylation inspired our prior biomimetic strategies to access the nocardioazines and related natural products^27–29^. Our earlier work also established that CDPSs NozA and NcdA from the *noz* and *ncd* genomic loci (Fig. 1a), respectively, each catalyze the assembly of *cyclo*-L-Trp-L-Trp (LL-cWW, **3**) as a plausible nocardioazine precursor^27,30^. Intriguingly, total syntheses established that **1**–**2** possess a 9 *R*,9’*R* (D,D-DKP) configuration^25,26^, which is inverted from the 9 *S*,9’*S* (L,L-DKP) configuration of putative precursor **3**. Further, heterologous expression of the *noz* cluster in *Streptomyces coelicolor* M1146 yielded **3** but no other candidate nocardioazine intermediates or products^27^. This led us to hypothesize that the nocardioazine pathway is distributed over multiple genomic loci, contrary to the typical paradigm of single loci encoding entire biosynthetic pathways^31^.

Herein, we report elucidation of the complete nocardioazine B (**2**) biosynthetic pathway, revealing that the LL-cWW (**3**) product of the *noz* or *ncd* locus is tailored by enzymes from the *noz2* locus to yield **2**. Our results link inversion of the L-configuration of **3** to a unique aspartate/glutamate (Asp/Glu) racemase homolog. We also implicate a member of the emerging phytoene synthase-like (PSL) family of prenyltransferases as the catalyst of *C*3’ prenylation of *cyclo*-D-Trp-D-Trp (DD-cWW, **5**), in contrast to other members that act upon L-configuration DKPs. Further, we report the biochemical characterization of an *S*-adenosyl-L-methionine (SAM) dependent methyltransferase that catalyzes both *N*1’ and *C*3 methylation of the asymmetric prenylated intermediate **6**, unveiling a rare catalyst of electrophilic methylation at two different nucleophilic acceptors to yield nocardioazine B.

## Results

### Distribution of the nocardioazine B pathway over multiple genomic loci

Bioinformatics analyses of the 7.2 Mbp *Nocardiopsis* sp. CMB-M0232 genome sequence revealed a locus (*noz2*, Fig. 1a) encoding three putative enzymes for tailoring of LL-cWW (**3**) to yield nocardioazine B (**2**): Asp/Glu racemase homolog NozR, PSL prenyltransferase homolog NozPT, and SAM-dependent methyltransferase homolog NozMT (Supplementary Table 1). To evaluate the involvement of *noz2* in nocardioazine biogenesis, this cluster was cloned using the nonintegrative *Escherichia coli*-*Streptomyces* shuttle plasmid pUWL201^32^ and the resulting construct or empty vector introduced into *S. lividans* TK24.

**Fig. 2:**
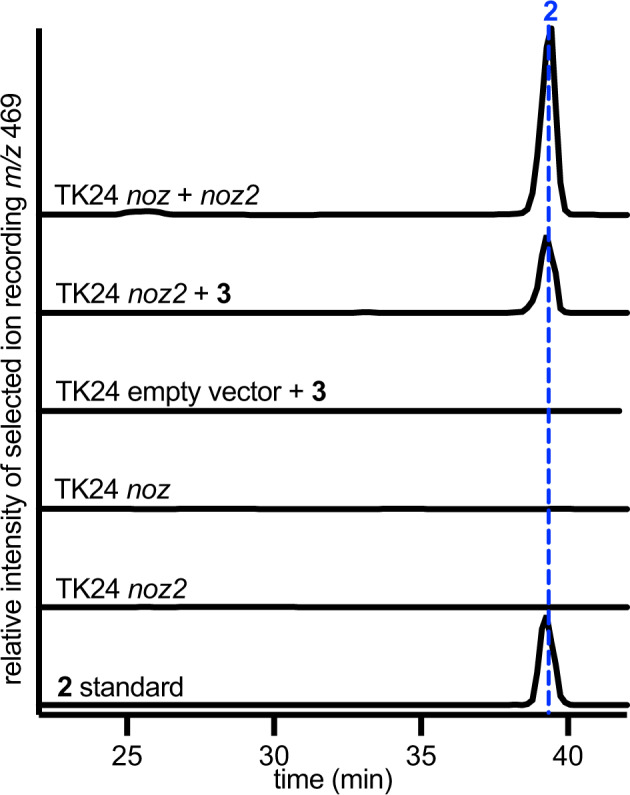
Multiple gene clusters from *Nocardiopsis* sp.CMB-M0232 are required for nocardioazine B (2) biogenesis. Heterologous co-expression of the *noz* and *noz2* clusters in *S. lividans* TK24 resulted in **2** production, based on LC-MS with positive-mode electrospray ionization (ESI^+^) selected ion recording (SIR) for [M + H]^+^ *m/z* 469. LC was conducted using a C_18_ column with gradient mobile phase of H_2_O/MeCN (40–90%) and 0.1% HOAc, as detailed in Methods. Transformants lacking either cluster failed to produce **2**. Chemical complementation of *noz2* transformants with LL-cWW (**3**) also yielded **2**, while complementation of empty pUWL201 vector transformants resulted in no accumulation of **2**. Source data are provided as a Source Data file.

Chemical complementation of resulting *noz2* cluster transformants with synthetic hypothesized precursor LL-cWW (**3**) resulted in the production of nocardioazine B (**2**) based on LC-MS^2^ analyses, while corresponding experiments with empty pUWL201 vector transformants failed to yield **2** (Fig. 2, Supplementary Fig. 1). Further, **2** was also produced by *S. lividans* TK24 co-transformants with both *noz2* and integrative cosmid pAL-*noz*, which harbors the *noz* cluster (including *nozA* CDPS) and was previously used for the heterologous production of **3** in *S. coelicolor* M1146^27^. Production of nocardioazine A (**1**) was not detected by LC-MS in any of these experiments (Supplementary Fig. 2). *S. lividans* TK24 transformants with *noz* but not *noz2* yielded **3** and no later stage nocardioazine intermediates; transformants with *noz2* but not *noz* produced no candidate intermediates unless supplemented with **3** (Fig. 2, Supplementary Fig. 3). These results establish that nocardioazine biogenesis requires multiple genomic loci; the *noz* or *ncd* locus encodes production of precursor **3** as established by our earlier studies^27,30^, while *noz2* encodes subsequent tailoring reactions of **3** to yield **2**.

### D/L DKP isomerization by a unique member of the Asp/Glu racemase family

**Fig. 3:**
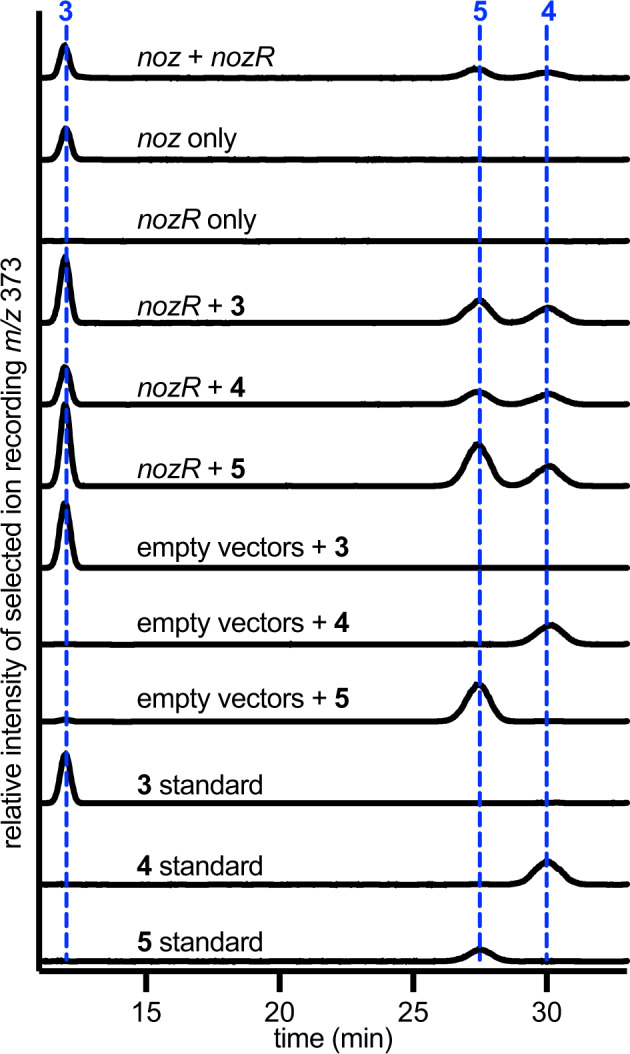
Heterologous expression and in vivo biotransformations support NozR as a D/L DKP isomerase. Heterologous co-expression of *noz* (encoding NozA CDPS and yielding **3**) with *nozR* in *S. lividans* TK24 resulted in the production of cWW stereoisomers, as revealed by LC-MS using ESI^+^ with SIR [M + H]^+^ *m/z* 373. LC was completed using a chiral cellulose column with isocratic mobile phase of H_2_O/MeCN (50/50) and 0.1% HOAc, as detailed in Methods. Likewise, chemical complementation of *S. lividans* TK24 *nozR* transformants with individual cWW isomers **3,** **4**, or **5** resulted in observation of all three cWW stereoisomers. Source data are provided as a Source Data file.

Aiming to establish the order and molecular details of LL-cWW (**3**) tailoring reactions to yield nocardioazine B (**2**), each of the three *noz2* cluster genes (i.e., *nozMT*, *nozR*, *nozPT*) were individually cloned using pUWL201. *S. lividans* TK24 was transformed with the resulting three constructs or with empty pUWL201 vector. Chemical complementation of each transformant with synthetic **3** and metabolite profiling by LC-MS with reversed-phase C_18_ and/or chiral cellulose stationary phases (detailed in Methods) revealed the production of candidate nocardioazine intermediates exclusively by transformants with *nozR* (Fig. 3, Supplementary Figs. 4, 5). Both *cyclo*-D-Trp-L-Trp (DL-cWW, **4**) and DD-cWW (**5**) were identified from these cultures by comparison of LC-MS profiles with synthetic cWW stereoisomers (Fig. 3), implying NozR as a D/L DKP isomerase. Analogously, transformants with both the *noz* cluster (including *nozA* CDPS yielding LL-cWW) and *nozR* also produced a mixture of cWW stereoisomers (Fig. 3), while transformants with both *noz* and *nozMT* or *nozPT* failed to produce candidate methylated or prenylated intermediates, respectively (Supplementary Figs. 4, 5). Together, these data support that the inverted DKP configuration of nocardioazines A-B (**1**–**2**) relative to precursor LL-cWW (**3**) is catalyzed by NozR, and that stereoisomerization precedes prenylation and methylation.

**Fig. 4:**
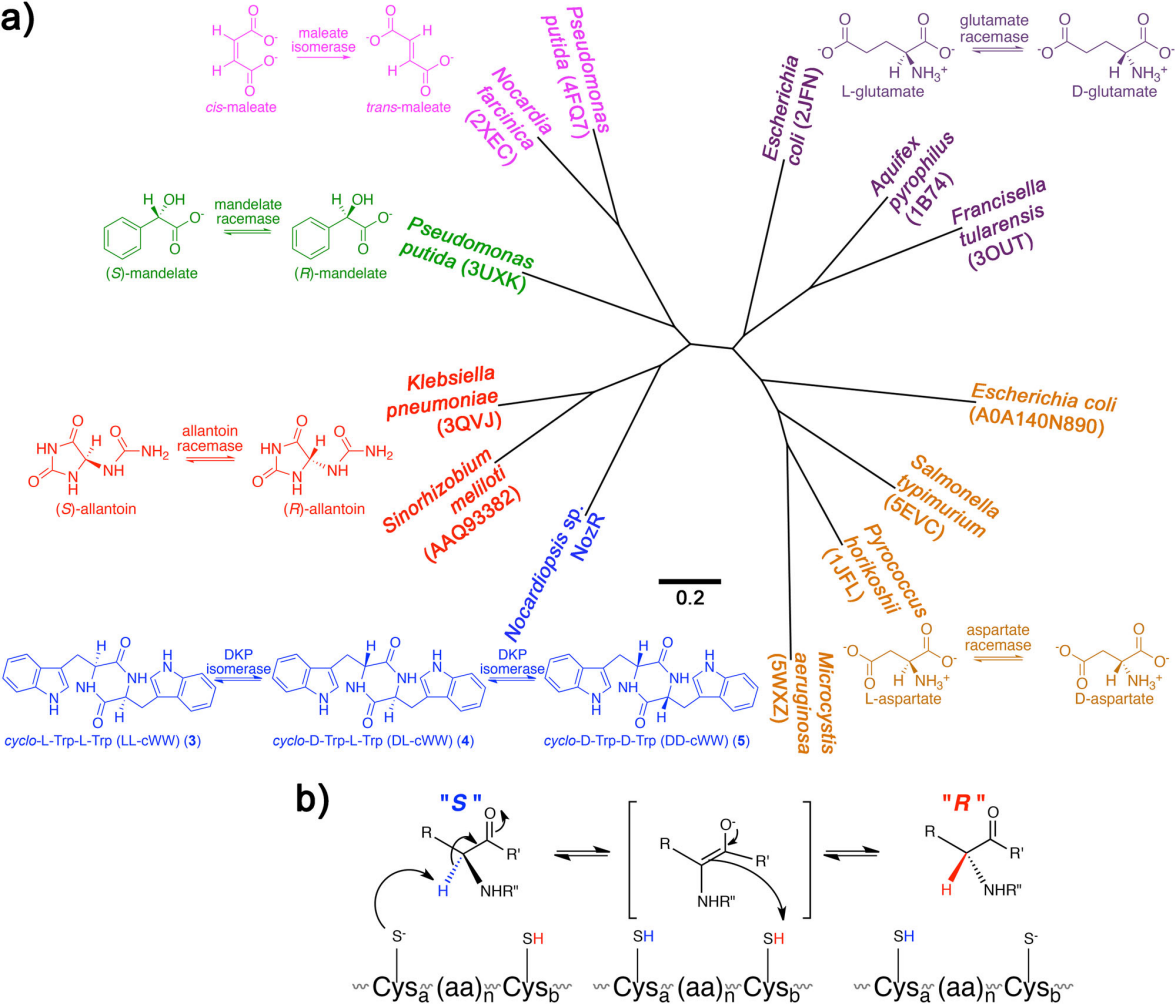
Relationship between DKP stereoisomerase NozR and representative Asp/Glu racemase family members^33–38^. **a** An unrooted neighbor-joining tree supports the phylogenetic relationship of NozR with representative functionally characterized members of the Asp/Glu racemase family. Species names and protein accession numbers for these isomerases are color-coded to match schematics of typical reactions catalyzed by each. **b** In prototypical reactions catalyzed by Asp/Glu racemases, one residue of the catalytic dyad (e.g. Cys_a_) deprotonates the α-H of a stereoisomer to yield an enolate intermediate, which is reprotonated via the other catalytic dyad residue (e.g. Cys_B_) from the opposite face of the molecule to yield reversible inversion of configuration.

Phylogenetic comparison of the NozR sequence with sequences of functionally characterized homologs supported NozR as a member of the widespread Asp/Glu racemase family (Fig. 4)^33–38^. Prototypical family members catalyze bidirectional racemization with an acid-base catalytic dyad, commonly of cysteine residues. These residues (Cys-75 and Cys-179) are conserved in NozR, suggesting that it also catalyzes reversible stereoisomerization (Fig. 4b, Supplementary Fig. 6). In support of this hypothesis, complementation of *S. lividans* TK24 *nozR* transformants with any single cWW stereoisomer resulted in production of mixtures of all three stereoisomers (Fig. 3).

To further confirm the Cys-75/Cys-179 catalytic dyad of NozR, pUWL201 constructs encoding NozR C75A, C179A, and C75A/C179A were created. Transformation of *S. lividans* TK24 with each of these three constructs followed by chemical complementation with individual cWW isomers **3**–**5** resulted in no detectable interconversion of stereoisomers (Supplementary Fig. 7), supporting the hypothesized catalytic roles of Cys-75 and Cys-179 (Fig. 4b).

**Fig. 5:**
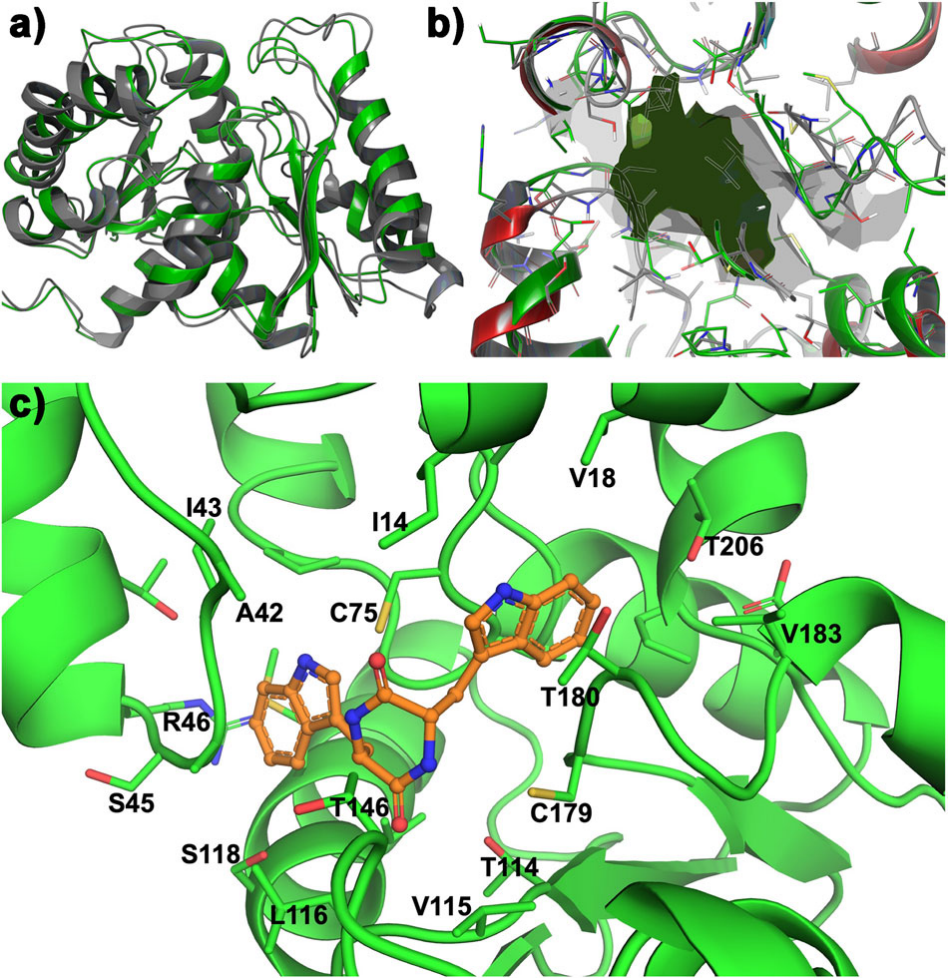
Molecular model of DKP D/L isomerase NozR and NozR complex with substrate LL-cWW (**3**). **a** Superposition of the NozR model (gray) with homolog allantoin racemase (green, PDB 3QVJ) revealed secondary and tertiary structure conservation. **b** Superposition of the binding pocket surface for NozR (gray) with that of allantoin racemase (dark green) revealed an expanded pocket for NozR, predicted to accommodate the DKP group and bulky indole residues of cWW. **c** Docking of **3** (orange carbon atoms) with NozR indicated accommodation of both the DKP moiety and indole groups. NozR residues within 4 Å of **3** are rendered as licorice sticks and labeled. Sulfur atoms from Cys-75 and Cys-179 of the catalytic dyad are 3.7 Å and 4.2 Å, respectively, from *C*9 of **3**. Supplementary Data 1 is a PDB file of this structure.

The structural contrast between cWW and previously reported substrates of Asp/Glu racemase family members (Fig. 4a) suggests unique details of DKP substrate recognition by NozR. Hence, we generated structural models of NozR to compare this DKP isomerase with other family members. Guided by crystal structures for other members of the Asp/Glu racemase superfamily^33–38^, a molecular model of NozR was developed using YASARA and Prime^39^. Superposition of the NozR structural model with allantoin racemase (PDB 3QVJ in Fig. 4) supported conservation of major secondary and tertiary structural features (Fig. 5a). The NozR structural model revealed an expanded substrate binding pocket relative to allantoin racemase (Fig. 5b). LL-cWW (**3**) was found to bind with high affinity in this pocket (binding ΔG −10.0 kcal/mol). The docking pose of **3** was quite striking, with the protein pocket having essential cavity spaces to accommodate the two bulky indole groups and DKP moiety (Fig. 5b, c, Supplementary Data 1). This cavity included the proposed Cys-75 and Cys-179 catalytic dyad with sulfur atoms of these residues positioned 3.7 Å and 4.2 Å, respectively, from *C*9 of **3** (Fig. 5c). Our docking studies also revealed several residues within 4 Å of **3** as candidates for recognition of this substrate (Fig. 5c).

### DKP prenylation by a phytoene synthase-like (PSL) prenyltransferase

**Fig. 6:**
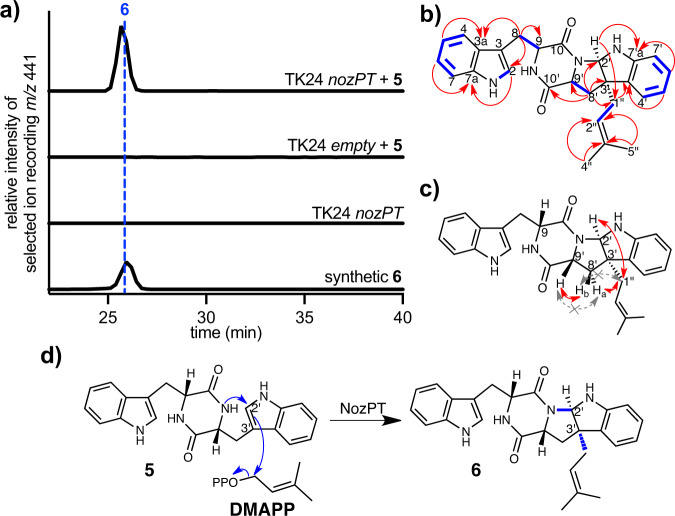
In vivo biotransformations implicate NozPT as an indole alkaloid DKP prenylation catalyst. **a** Heterologous expression of *nozPT* by *S. lividans* TK24 and complementation with DD-cWW (**5**) resulted in the production of prenylated **6**, with [M + H]^+^ *m/z* 441 by ESI^+^. LC was conducted with a reversed-phase C_18_ column and gradient mobile phase of H_2_O/MeCN (40–90%) containing 0.1% HOAc, detailed in “Methods”. **b** Atomic connectivity of **6** was established by key COSY (blue) and ^1^H-^13^C HMBC (red) correlations. **c** Key ROESY correlations (in red) established the configuration of **6**, with H-2’ and prenyl groups *exo* to H-9/H-9’; correlations in gray with X were absent or very weak compared to correlations in red. **d** The predicted mechanism for NozPT-mediated reactions includes DMAPP transfer onto **5** with *C*-*N* annulation to yield **6**. Source data are provided as a Source Data file.

With evidence for NozR catalyzing stereoisomerization of LL-cWW (**3**) to provide the 9 *R*,9 *R*’ (D,D-DKP) configuration reported for nocardioazine B (**2**)^25,26^, we hypothesized that NozPT and/or NozMT act upon DD-cWW (**5**). Chemical complementation of *S. lividans* TK24 *nozPT*, *nozMT*, or empty pUWL201 vector transformants with **3**–**5** and evaluation of resulting metabolite profiles revealed the production of candidate nocardioazine intermediates only by *nozPT* transformants supplemented with **4**–**5** (Fig. 6, Supplementary Figs. 4, 5). Trace amounts of a candidate prenylated intermediate were observed in these complementation experiments with **4**, precluding metabolite isolation and structure elucidation. Six-liter fermentation enabled purification and structure elucidation of the biotransformation product resulting from supplementation with **5**.

This metabolite was identified as *cyclo*-D-Trp-*C*3’-prenyl-D-Trp DKP (**6**) by detailed analyses of HRMS, 1D and 2D NMR spectra, and electronic circular dichroism (ECD) spectra (Supplementary Table 2; Supplementary Figs. 8–15). The atomic connectivity of **6** was established by ^1^H and ^13^C NMR spectral data in combination with HSQC, COSY and HMBC correlations (Fig. 6b), while the relative configuration of **6** was determined by ROESY correlations. Specifically, a strong ROESY correlation between H-2’ and H-1”s supported the *cis* orientation of H-2’ with the prenyl group at C-3’ (Fig. 6c). A strong correlation between H-9’ and H-8’b (not H-8’a) indicated these two protons reside on the same face of the molecule, while a correlation between H-1”s and H-8’a (not H-8’b) supported placement of these groups on the opposite face of the molecule (Supplementary Figs. 13, 14). These ROESY correlations, along with our observation of DD-cWW (**5**) (with 9 *R*, 9’*R* configuration) as the precursor for this prenylation reaction, supported the 2’*R*, 3’*R*, 9 *R*, 9’*R* absolute configuration proposed for **6**. This absolute configuration was further corroborated by agreement of ECD spectral data with synthetic **6** (Supplementary Fig. 15) prepared using methodology recently reported by our groups^28^.

An in vivo synthetic biology approach for hemiterpene production was recently established by engineering *E. coli* BL21(DE3) with a plasmid (pETDuet-PhoN-IPK) that overexpresses kinases PhoN and IPK to transform exogenous 3-methylbut-2-en-1-ol and 3-methylbut-3-en-1-ol into dimethylallyl pyrophosphate (DMAPP) and isopentyl pyrophosphate (IPP)^40^. We envisioned that the co-expression of this DMAPP/IPP production system with NozPT would both provide further functional evidence for NozPT and examine the feasibility of precursor-directed production of prenylated DKPs in *E. coli*. Hence, we cloned *nozPT* using expression vector pCDFDuet and co-transformed *E. coli* BL21(DE3) with the resulting pCDFDuet-nozPT construct and pETDuet-PhoN-IPK^40^. Induction of gene expression followed by supplementation of cell suspensions with DD-cWW (**5**), 3-methylbut-2-en-1-ol, and 3-methylbut-3-en-1-ol resulted in the production of prenylated **6**, while trials supplemented with LL-cWW (**3**) rather than **5** failed to produce prenylated product (Supplementary Fig. 16a). Similarly, the incubation of cell lysates from *E. coli* pCDFDuet-nozPT with **5** and DMAPP resulted in production of **6** (Supplementary Fig. 16b). Together, these results reaffirm **5** as a substrate of NozPT and imply that NozPT-mediated prenylation requires DMAPP (Fig. 6d).

NozPT shares greatest sequence similarity with phytoene synthase-like (PSL) family proteins, including ones demonstrated as indole alkaloid prenylation catalysts. These include NzsG (GenBank #ALL53320; 45% similarity) from the neocarazostatin pathway^41^, DmtC1 (#AVP32202; 51% similarity) from the drimentine DKP pathway^6^, SazB-PT (#CQR65853; 63% similarity) from the streptoazine DKP pathway^42^, and GczB (#RLV09121, 63% similarity) and GczC (#RLV08932, 64% similarity) from the griseocazine DKP pathway^15^. In contrast to the apparent preference of NozPT for DD-cWW, DmtC1, SazB-PT, GczB, and GczC act upon indole alkaloid DKP substrates composed of L-configuration amino acids.

### Functional characterization of a rare catalyst of electrophilic methylation at two different nucleophilic acceptors

We next aimed to establish the function of NozMT, the only methyltransferase homolog encoded within the *noz*, *ncd*, or *noz2* loci. Transformation of *S. lividans* TK24 with a pUWL201-based construct harboring *nozMT* and complementation with cWW stereoisomers **3**–**5** resulted in no production of candidate methylated cWW derivatives (Supplementary Fig. 4). Hence, we hypothesized that NozMT instead acts upon prenylated **6** to catalyze both *C*3 and *N*1’ methylation, yielding nocardioazine B (**2**). Supporting this hypothesis, the chemical complementation of *S. lividans* TK24 *nozMT* transformant cultures with **6** resulted in the production of **2** (Supplementary Fig. 17). These in vivo results suggest that NozMT catalyzes the transfer of methyl groups onto two different acceptor atoms (*C*3 and *N*1’) of asymmetrical prenylated substrate **6**.

**Fig. 7:**
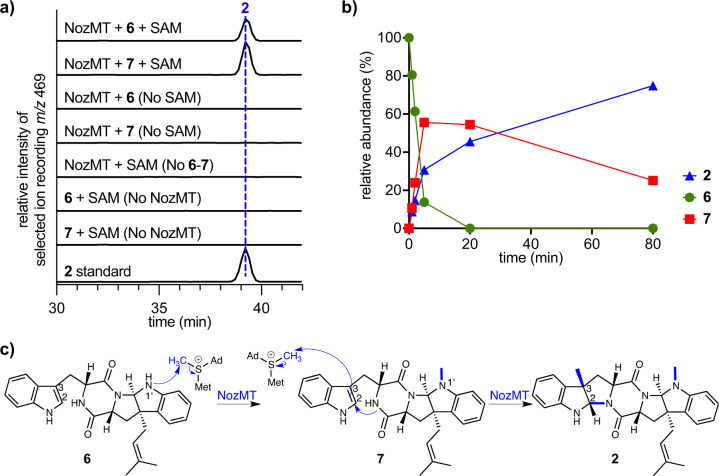
In vitro functional assays revealed that NozMT catalyzes *N*1’- followed by *C*3-methylation as the final steps in nocardioazine B assembly. **a** Incubation of purified recombinant NozMT with **6** or **7** and SAM yielded nocardioazine B (**2**), with [M + H]^+^ *m/z* 469 by ESI^+^. LC was conducted with a C_18_ stationary phase and gradient mobile phase of H_2_O/MeCN (40–90%) containing 0.1% HOAc, as detailed in Methods. **b** The time course of reactant **6,** *N*1’-methylated intermediate **7**, and product **2** relative concentrations during the NozMT-catalyzed reaction supports successive *N*1’- and *C*3-methylation (data points indicate means for *n* = 2 independent replicates). **c** Postulated mechanism of sequential methylation reactions of **6** catalyzed by NozMT. Source data are provided as a Source Data file.

To directly interrogate this unusual apparent dual function of NozMT, it was produced as a hexahistidine-tagged protein in *E. coli* and purified by Ni affinity chromatography (Supplementary Fig. 18). Incubation of NozMT with SAM as the electrophilic methyl donor and candidate substrates **3**–**6** revealed methylation of only prenylated **6** (Fig. 7a, Supplementary Fig. 19). Omission of SAM from reaction mixtures with **6** resulted in no nocardioazine B production (Fig. 7a), and the addition of metal cofactors was not required for catalytic activity.

To establish the order of *N*1’ and *C*3 methylation, enzymatic reaction mixtures were monitored by LC-MS for monomethylated intermediates and nocardioazine B (**2**) product over time. *N*1’ methylated **7** appeared as the only detectable monomethylated intermediate, and its concentration decreased in proportion to **2** formation (Fig. 7b, Supplementary Fig. 20). To directly confirm *N*1’ methylated **7** as a NozMT substrate, this compound was chemically synthesized from **6**. Incubation of synthetic *N*1’ methylated **7** with SAM and NozMT resulted in the production of **2** (Fig. 7a), unequivocally establishing *N*1’ methylation, followed by *C*3 methylation with annulation, as the final steps of **2** biosynthesis (Fig. 7c).

To compare the kinetics for NozMT-catalyzed *N*1’ methylation of **6** vs. *C*3 methylation of **7**, steady-state kinetic parameters of NozMT were evaluated by measuring initial reaction rates for a range of substrate concentrations with constant enzyme concentration. For *N*1’ methylation of **6**, an apparent average *K*_*m*_ of 146μM and *k*_cat_ of 1.5 s^−1^ (*n* = 2 independent replicates) were found; for *C*3 methylation of **7**, an apparent *K*_*m*_ of 606 μM and *k*_cat_ of 2.1 s^−1^ were determined (Supplementary Fig. 21). These values support a higher affinity and greater catalytic efficiency of NozMT for substrate **6** over **7**.

The NozMT sequence shares homology with functionally characterized DKP methyltransferases, including StspM1 (GenBank #QEI59523; 57% similarity) which catalyzes *C*3 methylation of LL-cWW (**3**)^43^. Previously characterized SazB-MT (#CQR65853), which catalyzes indole *N*1-methylation of **3**^42^, shares no significant sequence similarity with either NozMT or StspM1.

**Fig. 8:**
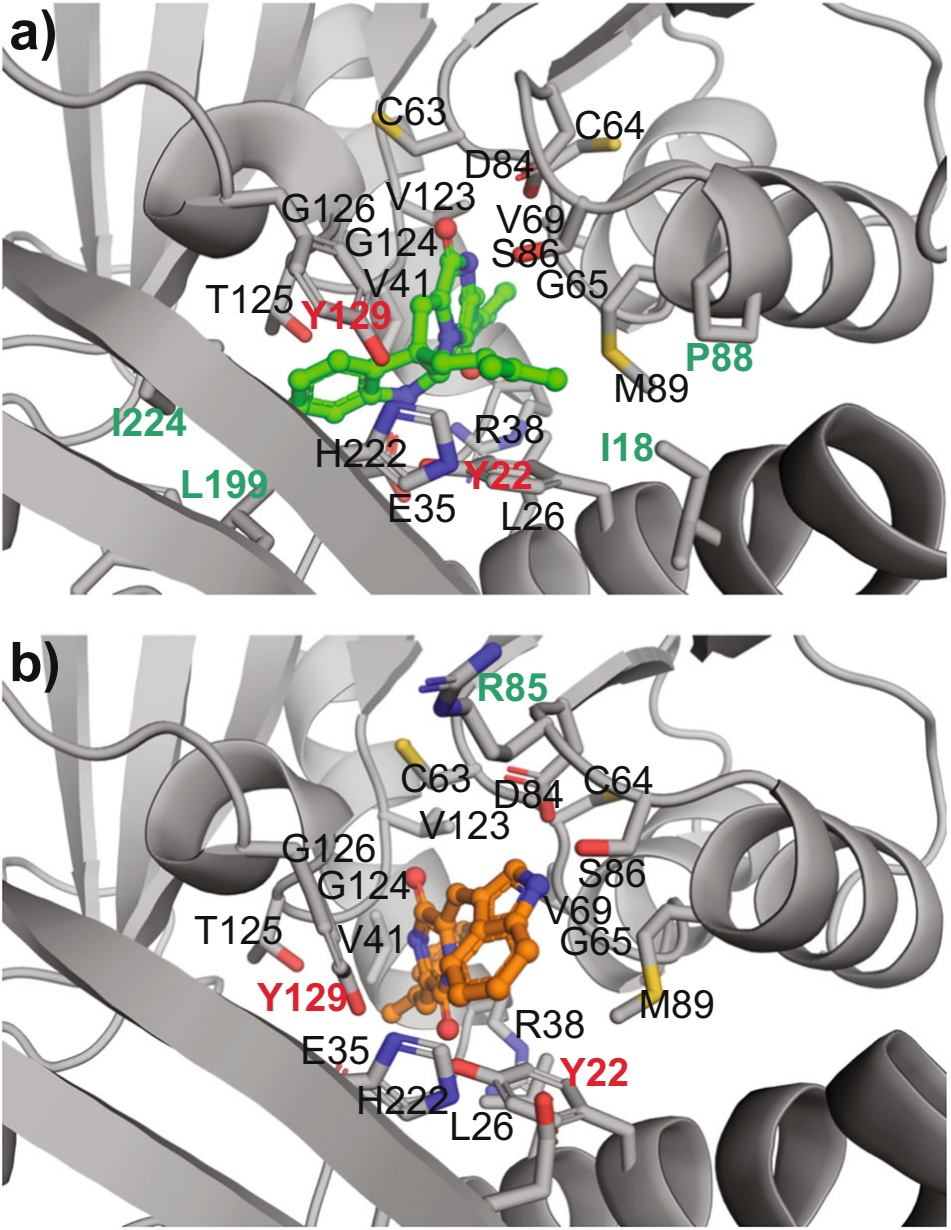
Molecular model of methyltransferase NozMT docked with tolerated substrate *cyclo*-D-Trp-*C*3’-prenyl-D-Trp (**6**) and rejected substrate DD-cWW (**5**). **a** Docking of **6** (green carbon atoms) with NozMT gave a binding ΔG of −12.6 kcal/mol. **b** Docking of **5** (orange carbon atoms) gave an inferior ΔG of −10.4 kcal/mol. In (**a**)–(**b**), NozMT residues within 4 Å of each candidate substrate are rendered as licorice sticks and labeled. Residues unique to NozMT binding of **5** vs. **6** are labeled in green, and suggest additional hydrophobic interactions with **6** (via Ile-18, Pro-88, Leu-199, Ile-224). Candidate catalytic residues Tyr-22 and Tyr-129, targeted in site-directed mutagenesis experiments, are labeled in bold red. Supplementary Data 2–3 provide PDB files of these docking structures.

Construction of a molecular model of NozMT using PHYRE^44^, Prime^45,46^, and YASARA^47–51^ supported that NozMT shares features with the class I methyltransferase superfamily, including a Rossmann-like fold with α-helices forming a sandwich around β-strands required for SAM binding (Supplementary Fig. 22a). The predicted NozMT active site was proximal to this conserved SAM binding site (Supplementary Fig. 22b)^52^. To probe the structural basis for NozMT tolerance of prenylated **6** and rejection of non-prenylated **5**, each of these DKPs was docked with the NozMT model (Supplementary Data 2–3). Docking of **6** with NozMT gave a binding ΔG of −12.6 kcal/mol compared to an inferior binding ΔG of −10.4 kcal/mol for **5**; the binding affinity of NozMT for **6** was 36-fold greater than for **5**. The greater number of hydrophobic contacts with **6** (via Ile-18, Pro-88, Leu-199, Ile-224) may account for its greater predicted binding affinity (Fig. 8).

Previously characterized metal cofactor-independent class I methyltransferases have been reported to act through proximity-desolvation or general acid-base mechanisms, both of which often include tyrosine residues^52,53^. This drew our attention to Tyr-22 and Tyr-129 from the docking-supported active site of NozMT (Fig. 8), and we aimed to probe the roles of these residues in substrate recognition and/or catalysis through site-directed mutagenesis. NozMT Y129F was readily produced as a soluble hexahistidine-tagged protein in *E. coli* and purified; NozR Y22F was insoluble, preventing evaluation of its catalytic activity. NozMT Y129F failed to catalyze methylation of either **6** or **7** (Supplementary Fig. 23), supporting an essential role of Tyr-129 in substrate recognition and/or catalysis.

## Discussion

Our work establishes that the nocardioazine B pathway is distributed over multiple genomic loci, in contrast to the typical paradigm of single loci encoding entire pathways^31^. Production of precursor LL-cWW (**3**) is encoded by either CDPS NozA or NcdA from the *noz* or *ncd* clusters, respectively^27,30^. Tailoring of **3** to yield nocardioazine B (**2**) requires the *noz2* cluster, encoding DKP D/L isomerase NozR, prenyltransferase NozPT, and methyltransferase NozMT.

Our results (Fig. 3) implicate NozR as a unique D/L isomerase acting upon a DKP substrate. Previously reported DKPs with one or more D-amino acid residues instead originated from the direct incorporation of D-amino acids via nonribosomal peptide synthetases (NRPSs), from NRPS epimerization domains acting upon peptidyl intermediates, or from nonenzymatic acid- or base-catalyzed mechanisms^1,54^. Hence, our findings unveil a unique paradigm for the biogenesis of DKPs containing D-amino acid residues. Since stereochemistry is a major determinant of biological function^55^, our findings open doors for the development of biosynthetic tools to craft DKPs with unique configurations and biological activities.

Bioinformatics analyses and structural models support NozR as a member of the widespread cofactor-independent Asp/Glu racemase family (Figs. 4,5). Other members of this family catalyze the stereoisomerization of common biomolecules, including amino acids and intermediates in purine degradation (Fig. 4a)^55,56^. Relative to previously reported substrates of members of this family, cWW is markedly bulkier. Recognition of cWW may be explained by a substrate binding pocket of NozR that is expanded relative to those of other Asp/Glu racemase family members, such as allantoin racemases (Fig. 5b).

Members of the Asp/Glu racemase family feature a conserved acid-base catalytic dyad^33–38^. A basic residue deprotonates the substrate α-H (e.g., H-9 or H-9’ of cWW) to yield a resonance-stabilized intermediate, which accepts a proton from an acidic residue on the opposite face of the molecule and results in the inversion of configuration at individual stereocenters (Fig. 4b). “Balanced” acid-base catalytic pairs (typically with two cysteines) result in reaction bidirectionality, while “unbalanced” pairs result in unidirectional reactions^33–38,55,56^. We hypothesize that the mechanism of NozR is consistent with these prior reports, since catalytic cysteine residues are conserved between NozR and other Asp/Glu racemase family members (Supplementary Fig. 6), our biotransformation experiments imply that NozR-catalyzed reactions are reversible (Fig. 3), and *S. lividans* TK24 cultures expressing NozR C75A, C179A, or C75A/C179A mutants failed to catalyze the stereoisomerization of cWWs **3**–**5** (Supplementary Fig. 7). Together, these results poise us for investigations of the molecular details of DKP D/L isomerase NozR and its potential as a biosynthetic tool for stereochemistry manipulation.

Our findings implicate NozPT as the catalyst of *C*3’ prenylation of DD-cWW (**5**). NozPT shares greatest sequence similarity with members of the emerging phytoene synthase-like (PSL) family of prenyltransferases. PSL family members do not share significant sequence similarity with the widespread indole prenyltransferase family, which includes members catalyzing reverse and normal prenylation at all indole group positions from diverse substrates that include numerous DKPs^57^. While very few PSL prenyltransferases have been experimentally characterized, bioinformatics analyses suggest these enzymes are frequently encoded in bacterial genomic loci with CDPSs^5^. Biochemically characterized PSL prenyltransferases include DmtC1, which catalyzes *C*3 farnesylation during drimentine DKP biogensis^6^, SazB-PT, which catalyzes *endo* prenylation of LL-cWW at *C*3 and *C*3’ during streptoazine assembly^42^, and GczB, which catalyzes *exo* prenylation of LL-cWW at *C*3 and *C*3’ during griseocazine assembly^15^. Expanding upon the substrate scope of this prenyltransferase family, our results provide evidence for a PSL prenyltransferase acting upon a D-configuration DKP. Observed products of these *C*3 and *C*3’ indole alkaloid DKP prenylation reactions all feature annulation. From our prior observation of annulation concomitant to *C*3’ biomimetic indole alkaloid DKP prenylation reactions^27–29^, we infer that annulation is likely spontaneous following enzyme-catalyzed prenylation at *C*3’.

Our results establish NozMT as the catalyst of electrophilic methyl transfer from SAM to both *N*1’ and *C*3 nucleophilic acceptor sites of asymmetrical prenylated intermediate **6** to yield nocardioazine B (**2**) (Fig. 7). While methyltransferases are ubiquitous in primary and secondary metabolism, enzymes mediating the methylation of multiple different acceptor atoms are exceptionally rare^52^. Among such methyltransferases, acceptor atoms are generally from the same group; for example, a SAM-dependent methyltransferase from *Arabidopsis* was found to catalyze halomethane formation from multiple halogen nucleophiles^58^. In contrast, NozMT catalyzes the methylation of atoms (*C*3 and *N*1’) from two different groups and hence marks an unusual addition to Nature’s biosynthetic repertoire.

Our finding that NozMT catalyzes the methylation of asymmetric prenylated **6** but not cWW stereoisomers **3**–**5** (Fig. 7a, Supplementary Figs. 4, 19) reveals the prenyl group and/or annulated scaffold of **6** as critical determinants of substrate recognition. This substrate specificity, along with methylation at multiple distinct sites of **6** in a specific order (Fig. 7b, c), suggests an intriguing relationship between the structure of NozMT and its catalytic activity.

Our NozMT structural model supported conservation of the Rossmann fold required for SAM binding between NozMT and other class I methyltransferases (Fig. 8, Supplementary Fig. 22a)^52^, and our docking studies predicted binding of substrate **6** at an active site proximal to the SAM binding site (Supplementary Fig. 22b). These docking studies suggested 36-fold greater binding affinity of NozMT with prenylated substrate **6** than non-prenylated **5**, in alignment with our enzyme assay finding that NozMT tolerated **6** but rejected **5** (Fig. 7, Supplementary Fig. 19).

Our in vitro assays required no metal supplements for NozMT catalytic activity (Fig. 7), supporting it as a metal cofactor-independent methyltransferase. Such natural product methyltransferases function through two typical mechanisms^52,53^. In the proximity-desolvation mechanism, the active site structure and chemical environment promote methylation by driving the nucleophilic acceptor of the substrate into close proximity to the SAM methyl group and excluding water from the interface between these groups. In prior studies of this mechanism, the mutagenesis of individual active site residues generally did not fully abolish catalytic activity^52,53^. The second metal-independent methylation mechanism involves general acid-base catalysis, where catalytic bases deprotonate the nucleophilic acceptor of the substrate to promote bond formation with the proximal electrophilic methyl group from SAM. In prior studies of this mechanism, the mutagenesis of catalytic residues typically resulted in abolition of enzyme activity^52,53^.

Both the proximity-desolvation and acid-base methylation mechanisms commonly involve tyrosine residues^52,53^. This prompted us to use site-directed mutagenesis to evaluate the roles of the two tyrosine residues (Tyr-22 and Tyr-129) within 4 Å of docked substrate **6** (Fig. 8a). While efforts to generate soluble NozMT Y22F failed, potentially due to a critical role of Tyr-22 in protein folding, NozMT Y129F was successfully produced and purified. The Y129F mutant failed to catalyze *N*1’ or *C*3’ methylation (Supplementary Fig. 23). Based on prior methyltransferase mechanistic studies^52,53^, this abolition of enzyme activity more strongly suggests an acid-base mechanism rather than a proximity-desolvation mechanism. Together, these results support an intriguing relationship between the NozMT structure, substrate recognition, and catalysis. The current work sets the stage for future in-depth structural biology investigations to further delineate the unique function of this DKP methyltransferase and probe its mechanistic details.

Nocardioazine B (**2**) was previously hypothesized as a precursor of nocardioazine A (**1**)^24^. We hypothesized that the two cytochrome P450 homologs encoded by the *noz* locus (Fig. 1a) may catalyze *C*2”-*C*3” epoxidation of the alkene group of nocardioazine B and formation of the *C*4”-*N*1 linkage to yield **1**. However, no production of **1** was observed for cultures of *S. lividans* TK24 transformants with both the *noz* and *noz2* clusters (Supplementary Fig. 2). It is plausible that these cytochrome P450s are not adequately expressed in the heterologous host; an alternative possibility is that enzymes catalyzing the transformation of **2** to **1** are encoded outside of the *noz* and *noz2* clusters.

The nocardioazine B biosynthetic pathway established herein revises the pathway previously postulated by bioinformatics analyses of an incomplete draft genome sequence of *Nocardiopsis* sp. CMB-M0232 and by comparison of metabolites from this strain with candidate intermediates prepared by chemical syntheses^27,30^. The *noz2* cluster, encoding all tailoring reactions of LL-cWW to yield nocardioazine B, was absent from the prior draft genome sequence. Further, in our earlier study, we isolated and identified annulated *cyclo*-*C*3-methyl-L-Trp-L-Trp DKP from *Nocardiopsis* sp. CMB-M0232 as a putative nocardioazine precursor^27^. The nocardioazine pathway established herein excludes this methylated biosynthetic intermediate, since our in vivo and in vitro experiments both refuted cWWs as NozMT substrates (Supplementary Figs. 4, 19). Instead, cryptic enzyme(s) outside of the nocardioazine loci reported herein apparently catalyze other methylation reactions of cWW from *Nocardiopsis* sp. CMB-M0232.

In summary, we established the nocardioazine B pathway, which is uniquely split over multiple genomic loci. This pathway provides evidence for a D/L isomerase acting upon DKP substrates, extends the emerging PSL prenyltransferase family, and unveils a rare methyltransferase catalyzing the electrophilic methylation of two distinct nucleophilic acceptors. These expanded paradigms for the biological assembly of DKPs, a prominent class of bioactive molecules, lay the foundation for future chemoenzymatic syntheses of novel DKPs and showcase Nature’s biomolecular ingenuity.

## Methods

### Genome sequencing, assembly, and bioinformatics analyses

*Nocardiopsis* sp. CMB-M0232 was fermented as previously described^24^, and genomic DNA isolated using the cetyl trimethyl ammonium bromide (CTAB) procedure for DNA isolation^59^. Genomic DNA library preparation, sequencing, and assembly were conducted by the National Center for Genome Resources (NCGR) using Pacific Biosciences (PacBio) sequencing technology. A size-selected DNA library was prepared using the PacBio size selected 20 kB protocol, sequenced using one PacBio SMRTcell, and de novo assembly conducted using HMAP to yield one contig of 7.2 Mbp. Putative open-reading frames and corresponding protein sequences were determined using GeneMarkS v4.28^60^. To hypothesize nocardioazine pathway enzymes, these protein sequences were queried for similarity to reported CDPSs, PSL-like prenyltransferases, and DKP methyltransferases using BLASTP in Geneious version 10 (Biomatters). Protein sequence alignments were prepared using ClustalW v1.2.2^61^, and phylogenetic trees constructed using Geneious version 10 Treebuilder.

### Cloning of *noz2* cluster gene(s) and transformation of *S. lividans* TK24

The following genes or clusters were cloned using *E. coli*-*Streptomyces* shuttle vector pUWL201 under the control of the ErmE promoter^32^: full *noz2* cluster (*nozMT, nozR, nozPT*), *nozMT*, *nozR*, and *nozPT*. Each gene insert along with the region ~20 nt upstream from the start codon was amplified by polymerase chain reaction (PCR) from *Nocardiopsis* sp. CMB-M0232 gDNA with Platinum^TM^ SuperFi^TM^ DNA polymerase (ThermoFisher Scientific) following manufacturer directions and supplementing reactions with 3% (v/v) DMSO. Oligonucleotide sequences are listed in Supplementary Table 3, and contained ~20-nt homology extensions corresponding to the HindIII (5’) and BamHI (3’) restriction site regions of pUWL201. Vector pUWL201 was restriction digested with HindIII and BamHI, and the linearized plasmid joined with each PCR product using HiFi Builder (New England Biolabs) following manufacturer directions. Resulting constructs were introduced by electroporation into *E. coli* NEB 10-beta (New England Biolabs), plasmid DNA purified, and gene inserts fully sequenced for confirmation. Methylation deficient *E. coli* ET12567 was transformed with at least two replicates of each construct, and constructs purified from resulting transformants. Each construct or empty pUWL201 was introduced into host *S. lividans* TK24 protoplasts via polyethylene glycol (PEG)-assisted transformation^59^. Transformants were selected based on thiostrepton (50 μg/mL) resistance and confirmed by colony PCR amplification of gene inserts and sequencing of resulting amplicons.

### Transformation of *S. lividans* TK24 with both the *noz* cluster and *noz2* gene(s)

Previously, we cloned the *noz* cluster using SuperCos 1 cosmid vector (Agilent), which was amended with an apramycin resistance cassette and genetic elements for integration into *Streptomyces* genomes^27^; this yielded pAL5571 (synonymous with pAL-noz). Herein, pAL-noz was introduced into *S. lividans* TK24 by intergeneric conjugation from *E. coli* ET12567/pUZ8002 using standard methods^59,62^. Integration of pAL-noz into the *S. lividans* TK24 chromosome was confirmed by PCR amplification and sequencing of selected genes spanning the entire *noz* cluster.

To generate *S. lividans* TK24 transformants with both the *noz* and *noz2* clusters, *S. lividans* TK24 pAL-noz protoplasts were transformed with a non-integrative pUWL201 construct containing the full *noz2* cluster using standard methods^59^. Transformants were selected by resistance to both apramycin and thiostrepton (50 μg/mL each) and confirmed by colony PCR amplification and sequencing of gene inserts from both the *noz* and *noz2* clusters. Analogous approaches were used to transform *S. lividans* TK24 pAL-noz with empty pUWL201 or with pUWL201 constructs carrying *nozMT, nozR*, or *nozPT*.

### Site-directed mutagenesis of D/L isomerase NozR

Constructs of pUWL201 encoding NozR site-directed mutants C75A, C179A, and double mutant C75A/C179A were prepared using the Agilent QuikChange XL II kit with primers listed in Supplementary Table 4. The manufacturer’s protocol was followed, except that 50 ng of DNA template were used per 25 μL reaction and polymerization was increased to 23 thermal cycles. *E. coli* XL10-Gold ultracompetent cells were transformed by heat shock with resulting constructs, and mutations confirmed by DNA sequencing of at least two replicate constructs. Constructs were introduced by electroporation into methylation deficient *E. coli* ET12567. Resulting demethylated constructs were purified and introduced into *S. lividans* TK24 protoplasts as described above. Resulting transformants encoding NozR mutants were evaluated alongside transformants encoding wild-type NozR for isomerization of cWWs **3**–**5** as outlined below.

### Heterologous expression and biotransformations using *S. lividans* TK24

To evaluate the above *S. lividans* TK24 transformants for production of nocardioazine intermediates and products, 5 mL cultures were fermented in saline M1 medium prepared as previously described^24^. Medium for transformants with *noz* (pAL-noz) was supplemented with apramycin (50 μg/mL), and medium for transformants with pUWL201 constructs were supplemented with thiostrepton (50 μg/mL). Cultures were fermented at 30 °C with shaking at 220 rpm for 6–8 days, and extracted with an equal volume of EtOAc. Extracts were concentrated in vacuo and resolubilized with MeOH (750 μL) for analyses by LC-MS. Heterologous biotransformation (chemical complementation) experiments were conducted equivalently, except that cultures were supplemented with a selected hypothesized precursor (500 μM) or DMSO vehicle. All heterologous expression and chemical complementation experiments were conducted at least in triplicate.

### Analyses of biosynthetic products by LC-MS

Chemical extracts resulting from heterologous expression and biotransformation experiments were subjected to LC-MS with ESI^+^ and SIR with MS^2^ for putative nocardioazine pathway intermediates using a Thermo LTQ-XL MS tuned to optimize DKP detection and interfaced with a Thermo UltiMate 3000 UHPLC system. Data were processed with Thermo XCalibur (Thermo Fisher Scientific) and exported to GraphPad Prism 9 for plotting. Metabolite retention times, SIRs, and MS^2^ fragmentation patterns were compared with synthetic standards or previously isolated nocardioazine B (**2**)^24,27,28^.

LC-MS experiments with a chiral stationary phase were conducted using a Lux 5 µ Cellulose 2 4.6 × 100 mm column (Phenomenex) and an isocratic mobile phase of 50/50 H_2_O/MeCN supplemented with 0.1% HOAc and flow rate of 0.2 mL/min. LC-MS experiments with a C_18_ stationary phase were completed using an Acclaim 120 5 µ C18 4.6 × 100 mm column (Thermo Scientific) and a gradient mobile phase consisting of an initial H_2_O/MeCN (60/40) hold for 5 min followed by a linear gradient to 90% MeCN over 30 min and hold at 90% MeCN for 7 min using a flow rate of 0.2 mL/min. All solvents were supplemented with 0.1% HOAc throughout.

### Isolation and identification of 6

*S. lividans* TK24 transformants with *nozPT* in pUWL201 were inoculated into six 2.8 L Fernbach flasks, each containing 1 L of saline M1 media supplemented with thiostrepton (50 μg/mL) and DD-cWW (**5**, 500 μM). Cultures were fermented at 30 °C with shaking at 250 rpm for eight days, extracted with an equal volume of EtOAc, and the resulting extract concentrated in vacuo. Biosynthetic intermediate **6** was partially purified by solid phase extraction using a Varian HF MEGA BE-C18 (10 g, 60 mL) column with mobile phase gradient from 20–100% MeOH in H_2_O, and **6** eluting in the fraction corresponding to 90% MeOH. Further purification was accomplished by HPLC, using an Agilent Zorbax SB-C18 column (9.4 × 250 mm, 5 μm) with a mobile phase consisting of an initial 3 min H_2_O/MeCN (60/40) hold followed by linear gradient to 90% MeCN over 22 min, and flow rate of 3.0 mL/min with the fraction collected at 13–16 min containing **6**.

The experimental exact mass of **6** was determined by high-resolution ESI^+^ MS using an Agilent 6545 Q-TOF instrument, which provided an [M + H]^+^ *m/z* 441.2289 (theoretical *m/z* 441.2291 for C_27_H_29_N_4_O_2_). Purified **6** (3 mg) was subjected to 1D (^13^C and ^1^H) and 2D (HSQC, HMBC, COSY, ROESY) NMR experiments using an Agilent VNMRS 500 MHz instrument with 5 mm AutoX DB PFG probe. NMR analyses were conducted in CDCl_3_ and processed using MestReNova v14 (MestReLab), with all spectral data referenced to residual chloroform signals (i.e. 77.1 ppm for ^13^C and 7.24 ppm for ^1^H). ECD spectral data were collected at an analyte concentration of 100 μM in MeOH using a Jasco J-1500 CD spectrometer.

### Production of 6 using engineered *E. coli* system

An *E. coli* codon-optimized synthetic gene encoding NozPT amended with EcoRI and HindIII restriction sites at the 5’ and 3’ ends, respectively, was synthesized by Eurofins Genomics using the sequence listed in Supplementary Information. This synthetic gene was subjected to restriction digestion with EcoRI and HindIII, then ligated at the corresponding sites of pCDFDuet-1 (Novagen) to yield pCDFDuet-nozPT, which was propagated using *E. coli* JM109 and whose sequence was confirmed by DNA insert sequencing. *E. coli* BL21(DE3) was transformed with this construct along with pETDuet-PhoN-IPK prepared in earlier work^40^. Transformants with pETDuet-PhoN-IPK and empty pCDFDuet were also generated.

Cultures of these *E. coli* BL21(DE3) transformants were prepared in LB medium supplemented with ampicillin (50 μg/mL) and streptomycin (50 μg/mL), grown to OD_600_ 0.5–0.6, and gene expression induced by supplementation with isopropyl β-D-1-thiogalactopyranoside (IPTG, 1 mM). Cultures were incubated for 12 h at 30 °C with shaking at 220 rpm, cells pelleted by centrifugation at 2000 × *g*, and washed 2× with phosphate-buffered saline (1 vol). Cell pellets were resuspended with M9 medium (0.1 vol) containing DKP **3** or **5** (1 mM), 3-methylbut-2-en-1-ol (5 mM), and 3-methylbut-3-ene-1-ol (5 mM). Controls lacking either alcohols or DKP were prepared equivalently. These bioreactions were incubated overnight at 30 °C with shaking at 220 rpm, extracted with EtOAc (2 vol), concentrated in vacuo, and resuspended with MeOH (1 vol) for analysis by LC-MS.

### Production of 6 using lysates from *E. coli* expressing NozPT

*E. coli* BL21(DE3) was transformed with either pCDFDuet-nozPT or empty pCDFDuet-1 vector. Cultures of these transformants were prepared in LB (50 mL) supplemented with streptomycin (50 μg/mL), grown at 37 °C with shaking (250 rpm) to OD_600_ ~ 0.5, induced with IPTG (1 mM), and incubated at 30 °C with shaking (250 rpm) for an additional 12 h. Cells were pelleted by centrifugation at 2000 × *g*, washed twice with an equal volume of lysis buffer (50 mM Tris pH 7.5, 50 mM NaCl, 2.5 mM MgCl_2_), resuspended with 4 mL of this buffer, and sonicated. Resulting lysates were centrifuged at 17,000×*g* for 10 min, and the supernatant passed through a 0.2 μm filter. In vitro assays were conducted by incubating lysate (500 μL) with **5** (500 μM) and/or DMAPP (500 μM) overnight at 30 °C, with at least two replicates of each sample type. Reactions were quenched by addition of MeCN (1 vol), and evaluated for product **6** using the above C_18_ stationary phase and H_2_O/MeCN gradient LC-MS method.

### Cloning, expression, and purification of methyltransferase NozMT

The gene encoding NozMT was codon optimized for *E. coli* expression and synthesized by Eurofins Genomics with BamHI and HindIII restriction sites at the 5’ and 3’ ends, respectively, using the sequence listed in Supplementary Information. This synthetic gene was subjected to restriction digestion with these enzymes, then ligated into the corresponding sites of pQE31 (Qiagen). The resulting pQE31-nozMT construct was propagated using *E. coli* JM109 and its sequence confirmed by DNA insert sequencing. This construct was introduced into *E. coli* M15(pREP4) for production of NozMT as an N-terminal hexahistidine-tagged protein. Cultures of *E. coli* pQE31-nozMT were prepared in LB supplemented with ampicillin (100 μg/mL) and kanamycin (25 μg/mL), incubated at 200 rpm and 37 °C to OD_600_ ~ 0.4, induced with IPTG (1 mM), and incubated for an additional 24 h at 220 rpm and 15 °C.

Cells (~1.3 g) were pelleted by centrifugation, resuspended with 14 mL lysis buffer (40 mM Tris pH 7.5, 200 mM NaCl, 10% v/v glycerol), and sonicated. Resulting lysates were centrifuged (17,000 × *g*, 4 °C, 30 min), and the supernatant filtered (0.45 μm). The resulting soluble protein mixture was supplemented with 20 mM imidazole and batch bound by incubation with Ni-NTA resin (2 mL, Thermo Scientific) on ice for 2 h. The mixture was poured into a column and eluted sequentially with 1 mL aliquots of lysis buffer containing increasing concentrations of imidazole (40 mM, 60 mM, 100 mM, 300 mM) followed by 3 mL of 500 mM imidazole. NozMT was enriched to >90% purity in the fraction corresponding to 500 mM imidazole, based on SDS-PAGE (Supplementary Fig. 18). NozMT was concentrated to 33 μM and the buffer exchanged to storage buffer (40 mM Tris pH 7.5, 200 mM NaCl, 10% v/v glycerol, 1 mM DTT) using a 10-kD cutoff centrifugal concentrator. Purified NozMT was flash frozen in an EtOH/dry ice mixture and stored at −80 °C. The yield of NozMT was ~1.6 mg/L.

### Site-directed mutagenesis of methyltransferase NozMT

Constructs of pQE31 encoding NozMT site-directed mutants Y22F and Y129F were prepared using the Agilent QuikChange XL II kit with primers listed in Supplementary Table 5 and following the manufacturer protocol. *E. coli* XL10-Gold ultracompetent cells were transformed by heat shock with resulting constructs, and mutations confirmed for at least two replicates by DNA sequencing. These constructs were introduced by heat shock into expression host *E. coli* M15 (pREP4). Recombinant NozMT Y129F mutant was produced and purified as described for NozMT above. For NozMT Y22F mutant, cultivation temperatures ranging from 15–37 °C, incubation times from 4–24 h, and IPTG concentrations of 0.1 and 1 mM were used unsuccessfully to generate soluble protein.

### Methyltransferase NozMT activity assays

In vitro assays to evaluate the function and substrate tolerance of NozMT and NozMT Y129F mutant were conducted by incubating enzyme (5 μM) with SAM (2 mM) and candidate substrate (1 mM) in assay buffer (40 mM Tris pH 7.5, 200 mM NaCl, 1% v/v DMSO) at a total volume of 100 μL overnight. Control assays were conducted by omitting NozMT, SAM, or substrate. Reactions were quenched by addition of MeOH (4.5 vol), and reaction products evaluated by LC-MS using the above C_18_ stationary phase and H_2_O/MeCN gradient method. To evaluate the order of *N*- and *C*-methylation of prenylated **6**, a time course experiment was conducted using the same parameters above except with 8 mM SAM. Reaction mixtures were quenched at timepoints ranging from 30 sec to 80 min and the above LC-MS method utilized to assess the relative ratios of prenylated **6** reactant, monomethylated intermediate **7**, and nocardioazine B (**2**) product based on peak areas for SIRs of *n* = 2 independent replicates.

### Evaluation of methyltransferase NozMT kinetics

Kinetics constants (*K*_*m*_, *V*_max_, *k*_cat_) for NozMT were evaluated by conducting a series of assays with variable substrate concentration and constant NozMT. A dilution series of ten concentrations of **6** (6–900 μM) and twelve concentrations of **7** (35–900 μM) were prepared in 200 μL of assay buffer containing excess SAM (2 mM). Mixtures were held at 37 °C and reactions initiated by addition of NozMT (10 μM). Two minutes was established as a reasonable time point for approximation of initial reaction rates, so reactions were quenched after two minutes by addition of MeCN (2 vol); reactants and products were quantified by analytical HPLC with UV detection at 210 nm. Analytical HPLC was conducted using a Zorbax SB-C18 column (Agilent 4.6 × 150 mm, 5 μm) with a 0.75 mL/min flow rate and H_2_O/MeCN gradient of 30–100% MeCN over 20 mins. All assays were conducted with *n* = 2 independent replicates. HPLC-UV data were processed using Agilent OpenLab v2.7. Rate vs. substrate concentration was plotted using GraphPad Prism 9 with nonlinear regression for Michaelis-Menten kinetics to calculate *K*_*m*_, *V*_max_, and *k*_cat_ and standard errors.

### DKP synthetic standards

Synthetic nocardioazine precursors were prepared, purified, and characterized in our earlier work^27,28^, except for *cyclo*-D-Trp-L-Trp DKP (**4**), *cyclo*-D-Trp-*C*3’-prenyl-D-Trp (**6**), and *cyclo*-D-Trp-*N*1’-methyl-*C*3’-prenyl-D-Trp (**7**). DKPs **4,** **6**, and **7** were prepared, purified, and characterized as described in Supplementary Methods and Supplementary Figs. 24–44, using methods established for their stereoisomers in our earlier work^27,28^.

### Macromolecular models

The isomerase NozR and methyltransferase NozMT sequences were used to generate complete structural models of each enzyme. The models were constructed based on multiple alignments, in which each domain was modeled as a separate unit, which were then assembled into a final composite hybrid model as further described in Supplemental Methods. The models were generated from consensus between the programs PHYRE v2.0^44^, Prime v3.0 (Schrodinger, LLC, New York, NY)^45,46^, and YASARA v21.8.26 SSP/Homology/PSSM Method^47–51^. The variable loops and gaps were filled using homology and knowledge-based potentials with YASARA. Each missing loop was modeled using the Loop Search module in Sybyl 8.0 or with YASARA loop modeler^47–51,63–65^. The selection of final loops was based on highest homology, as well as lowest root mean square deviations (RMSDs). The side chains and rotamers were adjusted with empirical potentials, simulated annealing with explicit solvent, and small equilibration simulations using YASARA’s refinement protocol. These were verified by WHAT-IF and PROCHECK^66^. Refinement of the hybrid models was carried out using a limited molecular dynamics-based refinement in YASARA consisting of the Secondary Structure Prediction (SSP) for YASARA parameterization, pKa assignment, solvation and simulated annealing and pre-equilibration setup, and energy minimizations^47–49,64^. Both homology and fold recognition were considered, and a final refinement with entire models were completed using YASARA for 250 ps of MD embedded in an empirical force field. The models were then subjected to energy optimization with Polak-Ribiere conjugate gradient (PRCG) with an R-dependent dielectric for finalization^67^. Further description is available in Supplemental Methods.

### Reporting summary

Further information on research design is available in the Nature Portfolio Reporting Summary linked to this article.

## Supplementary information

Supplementary Information

Description of Additional Supplementary Files

Supplementary Data 1

Supplementary Data 2

Supplementary Data 3

Reporting Summary

## Data Availability

The *noz2* cluster has been deposited in the GenBank database under accession code [https://www.ncbi.nlm.nih.gov/nuccore/MZ913435]. Other sequences from this study are available under the following accession codes: *noz* cluster KT184400; *ncd* cluster KT184401; representative racemases 2XEC, 3UXK, AAQ93382, 5WXZ, 1JFL, 5EVC, A0A140N890, 3OUT, 1B74, 2JFN, 4FQ7; representative prenyltransferases ALL53320, AVP32202, CQR65853, RLV09121, RLV08932; representative methyltransferases QEI59523, CQR65853. The NMR, UV-Vis, IR, ECD, LC-MS, and kinetics data generated in this study are provided in the Supplementary Information and/or Source Data. The macromolecular models generated in this study are provided as supplementary PDB files. Biological materials may be obtained upon request to the corresponding author. Source data are provided with this paper.

## Source data

Source Data
