# Supplementary material for: Unveiling an indole alkaloid diketopiperazine biosynthetic pathway that features a unique stereoisomerase and multifunctional methyltransferase
Source: Nat Commun. 2023 May 3;14:2558. doi: 10.1038/s41467-023-38168-3 (PMC10156859; doi:10.1038/s41467-023-38168-3)
Supplement: Supplementary file 1 — Supplementary Information [file 41467_2023_38168_MOESM1_ESM.pdf]

Supplementary Information

Unveiling an indole alkaloid diketopiperazine biosynthetic pathway that features a unique stereoisomerase and multifunctional methyltransferase

Garrett Deletti[1], Sajan D. Green[1], Caleb Weber[1], Kristen N. Patterson[1,5], Swapnil S. Joshi[2], Tushar M. Khopade[2], Mathew Coban[3], James Veek-Wilson[1], Thomas Caulfield[3,4], Rajesh Viswanathan[1,2,*], Amy L. Lane[1,*]

[1]Department of Chemistry & Biochemistry, University of North Florida, Jacksonville, FL 32224 USA
[2]Departments of Chemistry & Biology, Indian Institute of Science Education and Research Tirupati, Andhra Pradesh, India
[3]Department of Cancer Biology, Mayo Clinic, Jacksonville, FL 32224 USA
[4]Department of Neuroscience, Mayo Clinic, Jacksonville, FL 32224 USA
[5]Current address: Department of Chemistry, Emory University, Atlanta, GA 30322 USA
These authors contributed equally: Garrett Deletti, Sajan D. Green
*Correspondence and requests for materials should be addressed to A.L.L. (email: Amy.Lane@unf.edu) or R.V. (email: rajesh@iisertirupati.ac.in).

Table of Contents

Supplementary Methods: 5

DKP synthesis methods…………………………………………………………….. 5

Macromolecular modeling and docking methods……………………………… 11

Sequences of synthetic genes for expression of NozPT and NozMT………. 12

Supplementary Table 1. Annotation of *noz2* genomic region from *Nocardiopsis* sp. CMB-M0232. 13

Supplementary Figure 1. MS^2 spectra for nocardioazine B (**2**) resulting from heterologous expression and biotransformation experiments. 13

Supplementary Figure 2. LC-MS evaluation of *S. lividans* TK24 *noz* and/or *noz2* heterologous expression and biotransformation cultures for nocardioazine A. 14

Supplementary Figure 3. LC-MS evaluation of *S. lividans* TK24 *noz* and/or *noz2* heterologous expression and biotransformation cultures for candidate nocardioazine B intermediates. 15

Supplementary Figure 4. LC-MS evaluation of candidate methylated nocardioazine intermediates resulting from complementation of *S. lividans* TK24 *nozMT* with cWWs or heterologous expression of *noz* with *nozMT*. 18

Supplementary Figure 5. LC-MS evaluation of candidate prenylated nocardioazine intermediates resulting from complementation of *S. lividans* TK24 *nozPT* with cWWs or heterologous expression of *noz* with *nozPT*. **19**

Supplementary Figure 6. Alignment of NozR amino acid sequence with selected allantoin racemase sequences. **20**

Supplementary Figure 7. LC-MS evaluation of cWW stereoisomers resulting from complementation of *S. lividans* TK24 expressing wild-type NozR or C75A, C179A, or C75A/C179A mutants with individual cWW stereoisomers. **21**

Supplementary Table 2. Summary of ^{1}H, ^{13}C, HMBC, COSY, and ROESY NMR spectral data for biosynthetic *cyclo*-D-Trp-C3'-prenyl-D-Trp DKP (**6**) in $CDCl_3$. **22**

Supplementary Figure 8. ^{1}H NMR spectrum of biosynthetic *cyclo*-D-Trp-C3'-prenyl-D-Trp DKP (**6**). **23**

Supplementary Figure 9. ^{13}C NMR spectrum of biosynthetic *cyclo*-D-Trp-C3'-prenyl-D-Trp DKP (**6**). **24**

Supplementary Figure 10. HSQC spectrum of biosynthetic *cyclo*-D-Trp-C3'-prenyl-D-Trp DKP (**6**). **25**

Supplementary Figure 11. COSY spectrum of biosynthetic *cyclo*-D-Trp-C3'-prenyl-D-Trp DKP (**6**). **26**

Supplementary Figure 12. HMBC spectrum of biosynthetic *cyclo*-D-Trp-C3'-prenyl-D-Trp DKP (**6**). **27**

Supplementary Figure 13. ROESY spectrum of biosynthetic *cyclo*-D-Trp-C3'-prenyl-D-Trp DKP (**6**). **28**

Supplementary Figure 14. Enlargement of region of ROESY spectrum for **6** showing key correlations for relative stereochemistry determination. **29**

Supplementary Figure 15. Electronic circular dichroism (ECD) spectra for biosynthetic and synthetic **6**. **30**

Supplementary Figure 16. LC-MS evaluation of *cyclo*-D-Trp-C3'-prenyl-D-Trp (**6**) production by *E. coli* NozPT heterologous expression systems. **30**

Supplementary Figure 17. LC-MS evaluation of nocardioazine B production from complementation of *S. lividans* TK24 *nozMT* transformants with prenylated **6**. **31**

Supplementary Figure 18. SDS-PAGE analysis of purified recombinant NozMT. **31**

Supplementary Figure 19. LC-MS evaluation of cWW stereoisomers as NozMT substrates *in vitro*. **32**

Supplementary Figure 20. LC-MS evaluation of NozMT enzyme assay reaction products at selected time points. **33**

Supplementary Figure 21. Kinetics characterization of NozMT for methylation of **6** and **7**. **34**

Supplementary Figure 22. Structural model of dual function *N*- and *C*-methyltransferase NozMT and Connolly surface of NozMT docked with **6**. **35**

Supplementary Figure 23. *In vitro e*valuation of NozMT Y129F mutant for catalysis of **6-7** methylation. **36**

Supplementary Table 3. Sequences of oligonucleotides for PCR amplification of *noz2* cluster genes for cloning with pUWL201. **37**

Supplementary Table 4. Sequences of oligonucleotides for site-directed mutagenesis of *nozR* in pUWL201. **37**

Supplementary Table 5. Sequences of oligonucleotides for site-directed mutagenesis of *nozMT* in pQE31. **37**

Supplementary Figure 24. IR spectrum for L-Trp-N-Boc. **38**

Supplementary Figure 25. IR spectrum for D-Trp-OMe hydrochloride salt. **39**

Supplementary Figure 26. IR spectrum of L-Trp-D-Trp dipeptide. **40**

Supplementary Figure 27. IR spectrum of *cyclo*-L-Trp-D-Trp DKP (**4**). **41**

Supplementary Figure 28. UV-Vis spectrum for L-Trp-N-Boc. **42**

Supplementary Figure 29. UV-Vis spectrum for D-Trp-OMe hydrochloride salt. **43**

Supplementary Figure 30. UV-Vis spectrum of L-Trp-D-Trp dipeptide. **44**

Supplementary Figure 31. UV-Vis spectrum of *cyclo*-L-Trp-D-Trp DKP (**4**). **45**

Supplementary Figure 32. ^{1}H NMR spectrum for L-Trp-N-Boc. **46**

Supplementary Figure 33. ^{13}C NMR spectrum for L-Trp-N-Boc. **47**

Supplementary Figure 34. ^{1}H NMR spectrum for D-Trp-OMe hydrochloride salt. **48**

Supplementary Figure 35. ^{13}C NMR spectrum for D-Trp-OMe hydrochloride salt. **49**

Supplementary Figure 36. ^{1}H NMR spectrum for L-Trp-D-Trp dipeptide. **50**

Supplementary Figure 37. ^{13}C NMR spectrum for L-Trp-D-Trp dipeptide. **51**

Supplementary Figure 38. ^{1}H NMR spectrum for synthetic *cyclo*-L-Trp-D-Trp DKP (**4**). **52**

Supplementary Figure 39. ^{13}C NMR spectrum for synthetic *cyclo*-D-Trp-L-Trp (**4**). **53**

Supplementary Figure 40. ^{1}H NMR spectrum for synthetic *cyclo*-D-Trp-C3'-prenyl-D-Trp (**6**). **54**

Supplementary Figure 41. ^{13}C NMR spectrum for synthetic *cyclo*-D-Trp-C3'-prenyl-D-Trp (**6**). **55**

Supplementary Figure 42. ^{1}H NMR spectrum for synthetic *cyclo*-D-Trp-N1'-methyl-C3'-prenyl-D-Trp DKP (**7**). **56**

Supplementary Figure 43. ^{13}C NMR spectrum for synthetic *cyclo*-D-Trp-N1'-methyl-C3'-prenyl-D-Trp DKP (**7**). **57**

Supplementary Figure 44. ROESY NMR spectrum for synthetic *cyclo*-D-Trp-N1'-methyl-C3'-prenyl-D-Trp DKP (**7**). **58**

References from Supplementary Information **59**

Supplementary Methods

<u>DKP synthesis and purification</u>

<u>General Methods for DKP synthesis and purification:</u>

General Information and Reagents

Unless otherwise stated, each reaction was carried out under a blanket of argon, using standard syringe-septum and cannulation techniques. Amino acids and prenyl bromide were purchased from Sigma Aldrich or Spectrochem. EDC chloride and HOBt monohydrate were purchased from Spectrochem. Triethyl amine, formic acid, glacial acetic acid, and other general synthetic reagents were obtained from Rankem Chemicals or Fisher Scientific and were used without further purification. All commercially obtained synthetic compounds were at least 95% pure, and all commercially obtained solvents were at least 98% pure.

Chromatography

Thin-layer chromatography (TLC) was performed using silica gel 60 GF_{254} pre-coated aluminum backed plates (2.5 mm), specifically to monitor the progress of each chemical reaction and used as a guide for purification of the ensuing mixtures. Various combinations of EtOAc/hexane were used as eluent. Visualization of spots after TLC was accomplished by using phosphomolybdic acid (PMA) stain and UV light (254 nm). Synthetic products were purified using column chromatography (Silica gel grade: 200-400 mesh, 40-63 µm) with mobile phases listed for each compound below. Unless otherwise noted, yields refer to compounds isolated to analytical purity after chromatography.

Analytical Characterization

IR spectral data were collected using a Bruker Alpha FT-IR spectrophotometer. The samples for IR were prepared in ethanol. Similarly, the samples for UV-Vis measurements were prepared in ethanol and the absorbance was measured using an Agilent Cary Series UV-Vis-NIR ppectrophotometer. High-resolution mass spectral data (HRMS) were collected using time of flight (TOF) electrospray ionization in positive ionization mode (ESI^+). Electronic circular dichroism (ECD) spectra were collected in methanol using a Jasco J-1500 CD spectrometer.

NMR spectroscopic analyses were conducted for each product. ^{1}H NMR (400 MHz) and ^{13}C NMR (100 MHz) spectra for **4**, **7**, and intermediates leading to **4** were recorded on a 400 MHz spectrometer with sample temperature of 298K. The ^{1}H NMR (500 MHz) spectrum for **6** was collected using a 500 MHz spectrometer with sample temperature of 298K. Chemical shift values (δ) for NMR spectra are reported in parts per million (ppm) relative to the residual (indicated) solvent peak. Data for ^{1}H NMR are reported as follows: chemical shift (δ, ppm), multiplicity (s = singlet, brs = broad singlet, d = doublet, t = triplet, q = quartet, ddd = doublet of doublet of doublet, m = multiplet, cm = complex multiplet), coupling constants (in Hz), and integration corresponding to the number of protons. For ^{13}C NMR spectra, the type of carbon (C, CH, CH_2 or CH_3) was determined by Distortionless Enhancement by Polarization Transfer (DEPT) NMR experiment, and notations provided in parentheses after chemical shift.^{13}C NMR data is reported in parts per million (δ) relative to the residual (indicated) solvent peak.

Synthesis and purification of *cyclo*-D-Trp-L-Trp (4)

L-Trp-N-Boc carbamate:

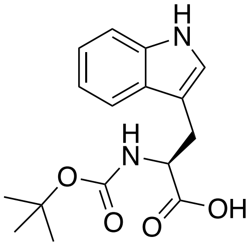

To a magnetically stirred clear solution of L-Trp (5.0 g, 25 mmol, 1.0 equiv.) in 250 mL of THF-H_2O (1:1) were added Na_2CO_3 (6.2 g, 75 mmol, 3.0 equiv.) and $NaHCO_3$ (7.8 g, 74 mmol, 3.0 equiv.). The resulting turbid solution (pH ~10.5) was cooled to 0 °C using H_2O/ice bath and stirred for 15 min. To this mixture was added Boc Anhydride (5.6 mL, 25 mmol, 1.0 equiv.) dropwise. The resulting solution was stirred for 15-20 min at 0 °C, the ice bath removed, and the reaction stirred at room temperature overnight. THF was removed *in vacuo*, and the product mixture was acidified by addition of a 1N aq. HCl. The mixture was then transferred to a separatory funnel and extracted three times with EtOAc. The combined organic phase was washed with brine, dried over anhydrous Na_2SO_4, and filtered. Concentration under reduced pressure gave 7.0 g (23 mmol, 93% yield) of white solid that was directly used for the next step without further purification. Images of IR, UV-Vis, ^{1}H NMR, and ^{13}C NMR spectral data are provided in Supplementary Figs. 24, 28, 32, and 33, respectively.

FT-IR (cm^{-1}): 516, 589, 744, 854, 1055, 1161, 1238, 1362, 1400, 1499, 1054, 1688, 2930, 2978, 3057, 3411.

^{1}H NMR (400 MHz, DMSO-*d*$_6$) δ 12.53 (s, 1H), 10.82 (s, 1H), 7.52 (d, *J* = 7.9 Hz, 1H), 7.33 (d, *J* = 8.1 Hz, 1H), 7.15 (t, *J* = 6.7 Hz, 1H), 7.09 – 7.03 (m, 1H), 7.01 – 6.93 (m, 2H), 4.14 (td, *J* = 9.0, 4.7 Hz, 1H), 3.13 (dd, *J* = 14.5, 4.6 Hz, 1H), 2.97 (dd, *J* = 14.6, 9.3 Hz, 1H), 1.33 (s, 9H).

^{13}C NMR (100 MHz, DMSO-*d*$_6$) δ 174.4, 155.9, 136.6, 127.6, 124.1, 121.4, 118.8, 118.6, 111.9, 110.6, 78.5, 55.0, 28.6, 27.3.

HRMS: Calc. for $C_{16}H_{21}N_2O_4$ $[M+H]^+$ *m/z* = 305.1501, found = 305.1498.

D-Trp methyl ester hydrochloride:

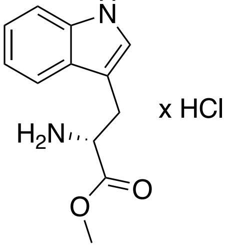

Thionyl chloride (8.9 mL, 123 mmol, 2.5 equiv.) was added drop wise to a cold (0 °C) solution of anhydrous MeOH (excess) under magnetic stirring. The solution was stirred at 0 °C for 30 min and then D-Trp (10.0 g, 48.9 mmol, 1.0 equiv.) was added and the resulting solution heated at 60 °C for 18 h. After evaporation of methanol *in vacuo*, a white residue of D-Trp methyl ester hydrochloride salt (10.5 g, 41.2 mmol, 84% yield) was obtained, which was used without further purification. Images of IR, UV-Vis, ^{1}H NMR, and

^{13}C NMR spectral data are provided in Supplementary Figs. 25, 29, 34, and 35, respectively.
FT-IR (cm^{-1}): 552, 622, 668, 740, 865, 941, 997, 1101, 1144, 1234, 1353, 1446, 1512, 1589, 1748, 2378, 2634, 2871, 3282, 3582, 3654, 3846, 3927.
^{1}H NMR (400 MHz, CD_3OD) δ 7.43 (d, *J* = 7.9 Hz, 1H), 7.30 (d, *J* = 8.1 Hz, 1H), 7.12 (s, 1H), 7.03 (t, *J* = 8.1 Hz, 1H), 6.96 (t, *J* = 7.5 Hz, 1H), 4.25 – 4.19 (m, 1H), 3.67 (s, 3H), 3.31 – 3.23 (m, 1H), 3.20 (dt, *J* = 3.2, 1.6 Hz, 1H).
^{13}C NMR (100 MHz, CD_3OD) δ 169.4 (Carbonyl C=O), 136.9 (C), 126.8 (C) 124.4 (CH), 121.5 (CH), 118.9 (CH) 117.5 (CH), 111.3 (CH), 106.1 (C), 53.3 (CH), 52.3 (CH_3), 26.2 (CH_2).

Methyl (tert-butoxycarbonyl)-D-tryptophyl-L-tryptophanate (L-Trp-D-Trp dipeptide):

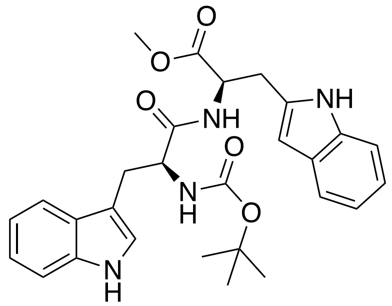

L-Trp-N-Boc (250 mg, 821 μmol, 1.0 equiv.) and D-Trp-OMe hydrochloride salt (209 mg, 821 μmol, 1.0 equiv.) were suspended in anhydrous dichloromethane (50 mL). The suspension was cooled to 0 °C in ice-water bath. This was followed by addition of triethyl amine (350 μL, 1.6 mmol, 2.0 equiv.) which resulted in a clear solution. N-hydroxybenzotriazole monohydrate (138 mg, 904 μmol, 1.1 equiv.) was then added to the reaction mixture, followed by addition of 1-ethyl-3-(3-dimethylaminopropyl)carbodiimide hydrochloride (173 mg, 904 μmol, 1.1 equiv.). This mixture was allowed to stir at 0 °C for one hour and then at room temperature for 15 hours. The product mixture was washed with saturated citric acid solution in a separatory funnel, followed by washing with saturated $NaHCO_3$ solution. The organic layer was dried over anhydrous Na_2SO_4 and solvent removed *in vacuo*. The dipeptide product was purified using column chromatography with silica stationary phase and 35-40% EtOAc/hexanes as eluent to yield methyl (tert-butoxycarbonyl)-D-tryptophyl-L-tryptophanate (351 mg, 695 μmol, 85% yield). Images of IR, UV-Vis, ^{1}H NMR, and ^{13}C NMR spectral data are provided in Supplementary Figs. 26, 30, 36, and 37, respectively.
FT-IR (cm^{-1}): 619, 675, 744, 857, 1054, 1102, 1167, 1239, 1362, 1446, 1509, 1667, 2930, 2974, 3059, 3405, 3739, 3869.
^{1}H NMR (400 MHz, DMSO-d_6) δ 10.87 (s, 1H), 10.75 (s, 1H), 8.34 (d, *J* = 7.7 Hz, 1H), 7.51 (m, 2H), 7.31 (t, *J* = 8.3 Hz, 2H), 7.13 (s, 1H), 7.09 – 7.03 (m, 2H), 7.02 – 6.95 (m, 2H), 6.92 (t, J = 7.5 Hz, 1H), 6.65 (d, J = 8.5 Hz, 1H), 4.51 (m, 1H), 4.25 (td, *J* = 9.0, 4.6 Hz, 1H), 3.59 (s, 3H), 3.14 (dd, *J* = 14.5, 5.7 Hz, 1H), 3.03 (dd, *J* = 14.5, 8.2 Hz, 1H), 2.93 (dd, *J* = 14.5, 4.5 Hz, 1H), 2.76 (dd, *J* = 14.6, 9.4 Hz, 1H), 1.28 (s, 9H).

^{13}C NMR (100 MHz, DMSO-d_6) δ 172.8, 172.4, 155.7, 136.6, 136.5, 127.6, 127.4, 124.2, 121.5, 121.3, 121.1, 119.0, 118.6, 118.5, 112.0, 111.8, 110.6, 109.8, 109.6, 80.6, 59.2, 56.4, 52.3, 28.6, 27.7.
HRMS: Calc. for $C_{28}H_{33}N_4O_5$ $[M+H]^+$ m/z = 505.2451, found: 505.2476.

Methyl D-tryptophyl-L-tryptophanate formic acid salt:
To the magnetically stirred solution of L-Trp-D-Trp dipeptide (351 mg, 695 μmol, 1.0 equiv.) in 50 mL of dichloromethane was added 50 mL of formic acid dropwise at room temperature. The reaction was allowed to stir for 12 hours, then DCM and formic acid were evaporated *in vacuo* to provide a reddish oil that was used for next step without further purification.

(3*R*,6*S*)-3,6-bis((1H-indol-3-yl)methyl)piperazine-2,5-dione (*cyclo*-L-Trp-D-Trp (4)):

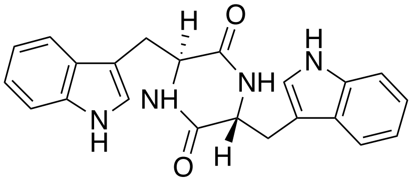

A homogenous solution of the above reddish oil was prepared in 14 M methanolic ammonia (pH ≈ 10, excess) and refluxed at 60 °C overnight with magnetic stirring. The solvent was removed *in vacuo*, and the resulting residue was washed with ice-cold water and sonicated until suspension. The suspension was vacuum filtered and the solid was washed with ice-cold MeCN (10 mL), which furnished pure *cyclo*-D-Trp-L-Trp DKP (**4**) as a white solid (228 mg, 612 μmol, 88% yield calculated for N-Boc deprotection followed by cyclization in methanolic ammonia). Images of IR, UV-Vis, ^1H NMR, and ^{13}C NMR spectral data are provided in Supplementary Figs. 27, 31, 38, and 39, respectively.
FT-IR (cm^{-1}): 535, 646, 741, 875, 1045, 1088, 1283, 1325, 1450, 1662, 2922, 2976, 3322, 3735.
^1H NMR (400 MHz, DMSO-d_6) δ 10.85 (s, 2H), 7.83 (s, 2H), 7.52 (d, J = 7.9 Hz, 2H), 7.31 (d, J = 8.1 Hz, 2H), 7.04 (m, 2H), 6.98 (m, 2H), 6.93 (m, 2H), 3.41 (m, 2H), 3.11 (dd, J = 14.5, 4.1 Hz, 2H), 2.86 (dd, J = 14.5, 4.5 Hz, 2H).
^{13}C NMR (100 MHz, DMSO-d_6) δ 168.1 (2 x C=O), 136.3 (2 x C), 128.1 (2 x C), 124.9 (2 x CH), 121.3 (2 x CH), 119.4 (2 x CH), 118.8 (2 x CH), 111.6 (2 x CH), 108.9 (2 x C), 55.1 (2 x CH), 28.8 (2 x CH_2).
HRMS: Calc. for $C_{22}H_{21}N_4O_2$ $[M+H]^+$ m/z = 373.1665, found: 373.1657.

Synthesis of *cyclo*-D-Trp-*C*3'-prenyl-D-Trp (6)

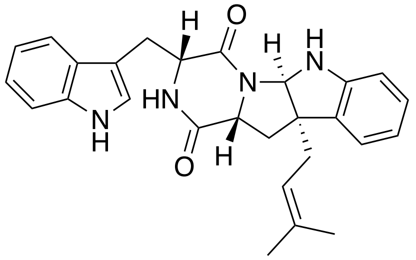

Cyclo-D-Trp-*C*3'-prenyl-D-Trp DKP (**6**) was synthesized following the same methodology as our recent synthesis of *ent*-**6**.[1] Briefly, DD-cWW (**5**, 100 mg, 269 μmol, 1.0 equiv.) was suspended in glacial acetic acid (5 mL). To this mixture, sodium acetate (44 mg, 538 μmol, 2.0 equiv.) followed by prenyl bromide (80 mg, 538 μmol, 2 equiv.) were added. The reaction was magnetically stirred overnight at room temperature and **6** purified using the semipreparative reversed-phase C_{18} HPLC method described for biosynthetic **6** in the main text to yield **6** as a white powder (45 mg, 102 μmol, 38% yield). Images of ECD and ^{1}H NMR spectral data are provided in Supplementary Figs. 15 and 40, respectively.
^{1}H NMR (500 MHz, Chloroform-*d*) δ 8.25 (s, 1H), 7.53 (dm, *J* = 8.0 Hz, 1H), 7.36 (dm, *J* = 8.2 Hz, 1H), 7.20 (tm, *J* = 7.5 Hz, 1H), 7.11 (m, 1H), 7.10 (m, 1H), 7.08 (m, 1H), 7.05 (m, 1H), 6.79 (t, *J* = 7.6 Hz, 1H), 6.75 (dm, *J* = 7.0 Hz, 1H), 5.75 (s, 1H), 5.40 (m, 1H), 5.12 (tm, *J* = 7.5 Hz, 1H), 4.32 (dm, *J* = 10.7 Hz, 1H), 3.97 (m, 1H), 3.71 (dd, *J* = 15.1, 3.7 Hz, 1H), 2.98 (dd, *J* = 15.1, 10.7 Hz, 1H), 2.58 (dd, *J* = 12.6, 6.1 Hz, 1H), 2.33-2.38 (m, 2H), 2.20 (dd, *J* = 12.9, 11.3 Hz, 1H), 1.69 (s, 3H), 1.52 (s, 3H).
^{13}C NMR (125 MHz, Chloroform-*d*) δ 169.2 (C), 166.4 (C), 149.0 (C), 136.8 (C), 136.0 (C), 131.3 (C), 128.9 (CH), 126.7 (C), 123.5 (CH), 123.4 (CH), 123.0 (CH), 120.3 (CH), 119.5 (CH), 118.6 (CH), 118.5 (CH), 111.7 (CH), 109.9 (C), 109.6 (CH), 79.7 (CH), 59.2 (CH), 55.8 (C), 54.7 (CH), 38.1 (CH_2), 35.6 (CH_2), 27.1 (CH_2), 26.2 (CH_3), 18.1 (CH_3)
HRMS: Calc. for $C_{27}H_{29}N_4O_2$ $[M+H]^+$ *m/z* = 441.2291, found: 441.2213.

Synthesis of *cyclo*-D-Trp-*N*1'-methyl-*C*3'-prenyl-D-Trp (7)

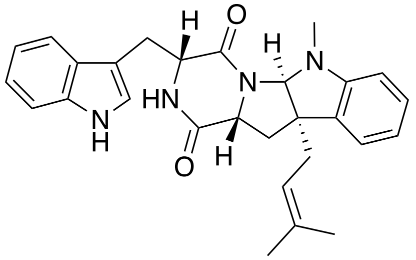

To a magnetically stirred ice-cold solution of *cyclo*-D-Trp-*C*3'-prenyl-D-Trp DKP (**6**) (25 mg, 57 μmol, 1.0 equiv.) in AcOH/MeCN (1:1, 1 mL) was added $NaBH_3CN$ (21 mg, 334 μmol, 5.8 equiv.). The reaction mixture was allowed to warm to room temperature with stirring for 1 hour. Saturated $NaHCO_3$ was then added to the mixture, and the organic layer was extracted with EtOAc (3 × 10 mL). The organic layers were combined, washed with brine, and dried over anhydrous Na_2SO_4. Reaction product **7** was purified using column chromatography with silica stationary phase and 1-2% MeOH in DCM as eluent

to provide **7** (18 mg, 40 μmol, 70% yield). Images of ^{1}H, ^{13}C, and ROESY NMR spectral data are provided in Supplementary Figs. 41-43.

^{1}H NMR (400 MHz, Chloroform-*d*) δ 8.34 (s, 1H), 7.61 (dd, *J* = 7.9, 1.0 Hz, 1H), 7.37 (dt, *J* = 8.1, 0.9 Hz, 1H), 7.21 (ddd, *J* = 8.2, 7.1, 1.2 Hz, 1H), 7.16 – 7.09 (m, 2H), 7.05 (d, *J* = 2.4 Hz, 1H), 6.97 (dd, *J* = 7.3, 1.3 Hz, 1H), 6.67 (td, *J* = 7.4, 1.0 Hz, 1H), 6.39 (d, *J* = 7.8 Hz, 1H), 5.98 (s, 1H), 5.38 (s, 1H), 5.04 – 4.91 (m, 1H), 4.38 (ddd, *J* = 9.6, 3.9, 1.8 Hz, 1H), 3.94 (ddd, *J* = 11.4, 5.9, 1.8 Hz, 1H), 3.63 (ddd, *J* = 14.9, 3.7, 1.0 Hz, 1H), 3.10 (m, 1H), 3.07 (s, 3H), 2.49 (dd, *J* = 12.4, 6.0 Hz, 1H), 2.22 (t, *J* = 6.1 Hz, 2H), 1.80 (dd, *J* = 12.3, 11.4 Hz, 1H), 1.67 (s, 3H), 1.51 (s, 3H).

^{13}C NMR (125 MHz, Chloroform-*d*) δ 168.5 (C), 165.5 (C), 136.6 (C), 135.7 (C), 132.1 (C), 129.0 (CH), 126.9 (C), 123.8 (CH), 123.7 (C), 123.3 (CH), 122.9 (CH), 120.2 (CH), 118.8 (CH), 118.7 (CH), 118.6 (CH), 111.6 (CH), 109.6 (C), 106.9 (CH), 85.7 (CH), 58.8 (CH), 54.9 (CH), 54.5 (C), 41.0 (CH_2), 36.8 (CH_2), 34.1 (CH_3), 28.3 (CH_2), 26.1 (CH_3), 18.2 (CH_3)

$[\alpha]\frac{250}{D}$ +110.1 (*c* 0.13, MeOH)

Stereoisomerase NozR and Methyltransferase NozMT Modeling and Docking

Modeling

NozR homology model was constructed using the sequence:
MRLTVIAPTSDPAILDLVRDEAAGWASPGTAIDVAGLPSTAAIASRHDTALAVPDLLAAI
GKAERSGSEGVLVSCAGDPAVHEARETAAIPVVGGFQPAALTALSLGERLGFITVLPSV
VPMLRALIRSEGLTGRVGTIRALGADTLDVHDTGALTGLIREQAADILLRDEADVIVLGC
TGFVGAAARVQQDLADTGFDVPVVDPTGAAVLWLESLVRLGVRSSRTTYHPPPTMTW
LA. The hybrid composite model was generated using PDB codes: 2EQ5, 3QVJ, 3QVL, 5LG5.[2,3]

NozMT homology model was constructed using the sequence:
MTAPATAPHDHPQPYATIADSYDRLLDWSSRHWDESPRTRVGDFLDTLWAGRAEPV
RTVIELCCGTGAVLEELAERGYTVAGLDRSAPMLEQARARLGPAVDLVHSELPDIPAR
ATYDALISVGTGLNYLASPDDLARTLESMARTVRPGGPVVFDLLSVRMLTVDAVEGFS
QRPIVEFDDSSFFWTYEHNVAEGHCDLTYTRFQRCPGTSEELFTRTRELHRIHLRERD
LVERLAREAGFTDIAVYDNYAHRPASAETQYEVWTMVAPS. The final hybrid model was generated with fragments from PDB codes: 3D2L, 3PFG, 1WZN, and 6M82.[4,5]

Docking

Docking for compounds **3, 5,** and **6** against the final models of NozMT and/or NozR were carried out using AutoDock4 Vina[6] plugin with YASARA. For NozMT, since the active site is not strictly characterized, pocket searching was carried out in proximity to the SAM binding site, and the most ideal pocket was selected for initial pose screening. AutoDock Vina was also used to provide an estimation of binding free energy. Visualizations, pose evaluation, distance measurements, and other analyses were carried out using PyMOL v2.0 (The PyMOL Molecular Graphics System, Schrödinger, LLC).

Sequences of Synthetic Genes for Expression of NozPT and NozMT in *E. coli*

Sequence of synthetic gene for expression of prenyltransferase NozPT in *E. coli*. The sequence is listed from 5'- to 3'-, and EcoRI (at 5' end) and HindIII (at 3' end) restriction sites used for cloning with pCDFDuet-1 vector are underlined:
AGAGGAATTCGAGCACTCGGGAATTGGATGCCGCCGGCTTTCGGGACTTGCGTCTG
CGTGATGCCTATGGCACGTGTCAGACCTTCCTCCGCCATCGTAACTCAGCCACGTAT
CCAGCGGCCTCTACACTGCTGCCGCCCGGAAAACGTCCGTATTGGGACGCCGTCTT
TGCGTTTACCACCTATGCTGACGATCTGATCGATGATCCGTTACGTCCGCCTGAATC
GCGCGCTGCGCGCTTTGATGAGTTCGAGCGCATGTTCTTCCTGCTGCTGAAAAGCGA
TCATCCGTGGCGCGAAGCGCCTGCGGAAGGTCAAGGTCGCCTCGCACGCCGTCTG
AGTCTGGCGTTTCTGCACACCGTCCGCACCTGGGGCATCTCGGAAGAGAGCATTCG
CCAGTTCATGTCCACGATTCGTACCGATCTGCACACCACAGACTACCCCGCATTTGC
GGATCTGCATGCCTACATTCACGGTGTTTGCGCAGTAGGCACGCAGTGGAACGTGG
AACTCCTGGAACCACACGATGACGAAGCCGCGCGCCGTGCTGCCAGCATGTCTGTG
GGGTTGCAGCTTACCGACATCCTTCTGGACTTGCGCGAAGATCTGGCAGTAGGACG
CCTGTATCTGCCGGTGGAAGATTTGCGCCGCTTTGGCCTGACTCGCGCTGACGTCG
AAGATGCTGCGGCGCATGGTCGCTTAACAGACCCGTTACGCGAGTTAGTGCGCTTTG
AGGCAGATCGTGCGCGCAGTGCGTTTGCGGATGCCGGTGATTGGTGGCGCTTAGCA
CATCCGTGCACTCGTGAGTTACCGCGCCTGTACATGCAGCTCGGCCGTGCAAGCCT
GGAATCCATTGTGCGCGCCCGTTTCGACGTCCTGAATCCGGTTCGCGCAGGGCGGC
TTGGCGATACGGCGCGTGCGTGTGGTGCGTCAGCGCTTGCCTACTCGCGTGCCTGG
CGTGTGCGCGCAGCTGCTCGTGGCCGTCCGGTAACCGTTCAACCAGCACCAGTTGG
GCCTCGGAAGCTTAGAG

Sequence of synthetic gene for expression of methyltransferase NozMT in *E. coli*. The sequence is listed from 5'- to 3'-, and BamHI (at 5' end) and HindIII (at 3' end) restriction sites used for cloning with pQE31 vector are underlined:
AGAGGGATCCAATGACAGCGCCAGCAACCGCACCACATGATCACCCTCAGCCATA
TGCAACTATCGCCGACTCGTATGATCGTCTTTTGGATTGGTCCTCACGTCATTGGG
ATGAAAGCCCTCGTACTCGCGTAGGTGATTTCCTAGATACGTTATGGGCAGGGCG
GGCGGAGCCGGTTCGTACCGTTATTGAGTTATGTTGCGGTACGGGTGCGGTGTTG
GAGGAACTTGCAGAGCGGGGTTATACGGTGGCGGGCCTCGACAGATCGGCTCCG
ATGCTCGAGCAAGCCCGCGCACGTCTGGGGCCTGCCGTCGACTTAGTGCACTCTG
AGTTGCCCGATATCCCCGCCCGTGCGACATATGACGCCCTTATTTCCGTTGGAAC
GGGACTCAACTATCTGGCCTCACCGGACGATCTGGCTCGCACACTGGAGTCGATG
GCGCGTACTGTACGCCCGGGTGGCCCGGTCGTATTTGATCTGCTGAGCGTTAGGA
TGCTGACGGTTGACGCCGTGGAAGGCTTTTCTCAACGCCCGATTGTCGAATTCGA
CGACAGCAGCTTCTTCTGGACTTATGAACACAACGTGGCCGAAGGCCACTGCGAT
CTGACCTACACGCGCTTTCAGCGTTGTCCGGGCACCAGTGAAGAACTGTTTACCC
GTACCCGAGAACTGCATCGCATCCATCTGCGCGAACGCGATCTGGTCGAACGACT
GGCGCGCGAAGCTGGCTTTACCGATATTGCTGTGTACGATAATTACGCGCATCGT
CCGGCGAGTGCTGAAACCCAGTACGAAGTGTGGACCATGGTGGCGCCGAGCAAG
CTTAGAG

Supplementary Table 1. Annotation of the *noz2* genomic region (GenBank Accession #MZ913435) from *Nocardiopsis* sp. CMB-M0232. Current work revealed that genes shown in blue/bold encode tailoring of LL-cWW (**3**) to yield nocardioazine B (**2**). Annotations of the *noz* (#KT184400) and *ncd* (#KT184401) loci, which each encode for production of **3**, were previously reported.[7,8]

gene	translated protein size (#aa)	annotation	NCBI accession number of homolog	identity/ similarity (%)
orf5324	286	NADP(H)-binding protein	WP_012887122	64/75
orf5325	266	PIG-L family deacetylase	WP_106581080	75/84
orf5326	174	TMEM165/GDT1 family protein	WP_106585140	82/90
nozMT	**270**	**SAM-dependent methyltransferase**	**WP_014151049**	**49/61**
nozR	**238**	**DKP D/L-isomerase**	**WP_214980922**	**54/69**
nozPT	**337**	**squalene/phytoene synthase like (PSL) prenyltransferase**	**WP_014151343**	**52/63**
orf5330	403	sensor histidine kinase	PSK96551	60/69
orf5331	255	response regulator transcription factor	PSK96552	75/80
orf5332	509	MFS transporter	WP_114024791	65/74

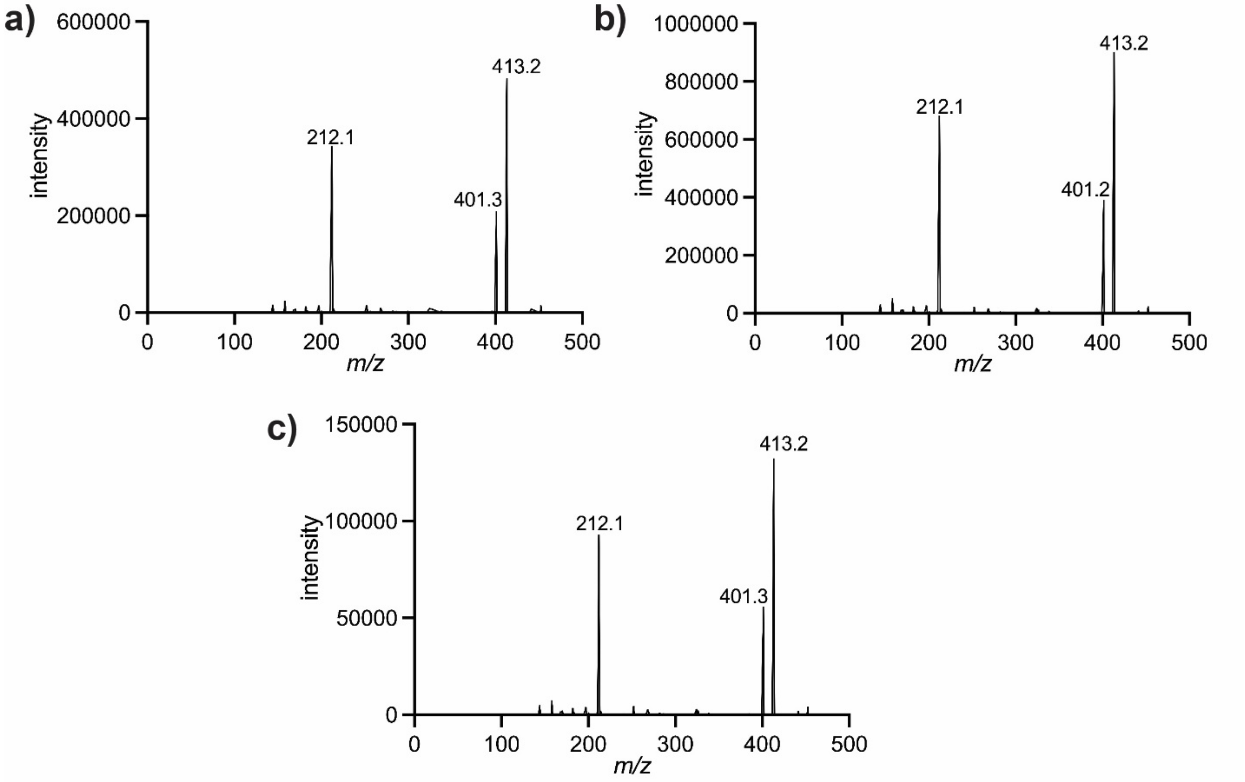

Supplementary Figure 1. MS^2 spectra for nocardioazine B resulting from heterologous expression and biotransformation experiments. MS^2 spectra of $[M+H]^+$ *m/z* 469 for **(a) 2** standard isolated from *Nocardiopsis* sp. CMB-M0232, **(b) 2** resulting from the chemical complementation of *S. lividans* TK24 *noz2* transformants with synthetic LL-cWW (**3**), and **(c) 2** resulting from the heterologous co-expression of both *noz* and *noz2* gene clusters in *S. lividans* TK24. LC-MS^1 selected ion recording (SIR) *m/z* 469 traces corresponding to these MS^2 data are provided in main text Fig. 2. Source data are provided as a Source Data file.

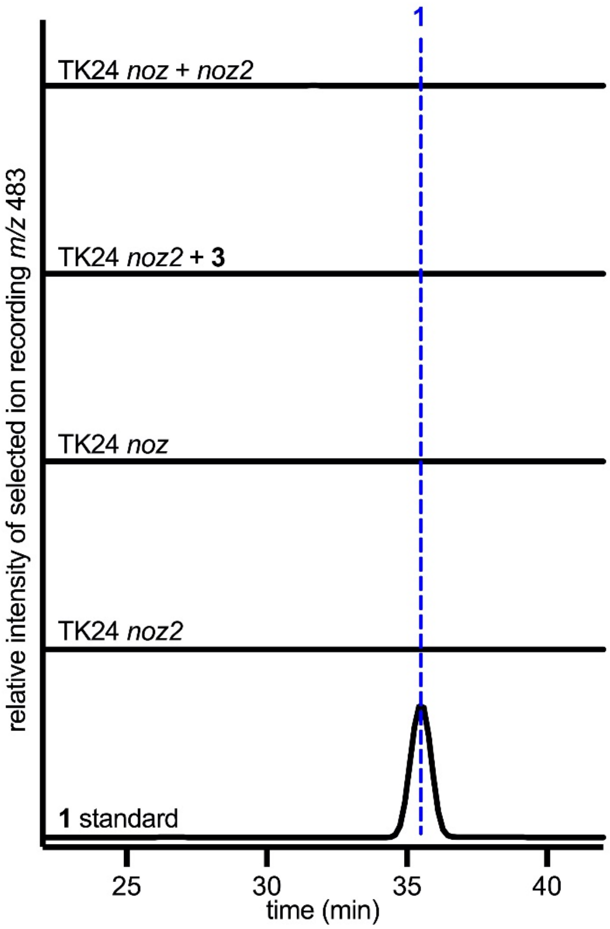

Supplementary Figure 2. Evaluation of *S. lividans* TK24 *noz* and/or *noz2* heterologous expression and biotransformation cultures for nocardioazine A (1). LC-MS analyses revealed that neither the chemical complementation of *S. lividans* TK24 *noz2* transformants with synthetic precursor LL-cWW (**3**) nor the heterologous expression of *noz*, *noz2*, or both of these clusters resulted in production of **1**. LC-MS was conducted with analyte separation by a C_{18} stationary phase with H_2O/MeCN gradient (detailed in main text Methods) and detection by positive-mode electrospray ionization (ESI^+). LC-MS traces show selected ion recording (SIR) for $[M+H]^+$ *m/z* 483, corresponding to **1** from *Nocardiopsis* sp. CMB-M0232. Source data are provided as a Source Data file.

a) **b)**

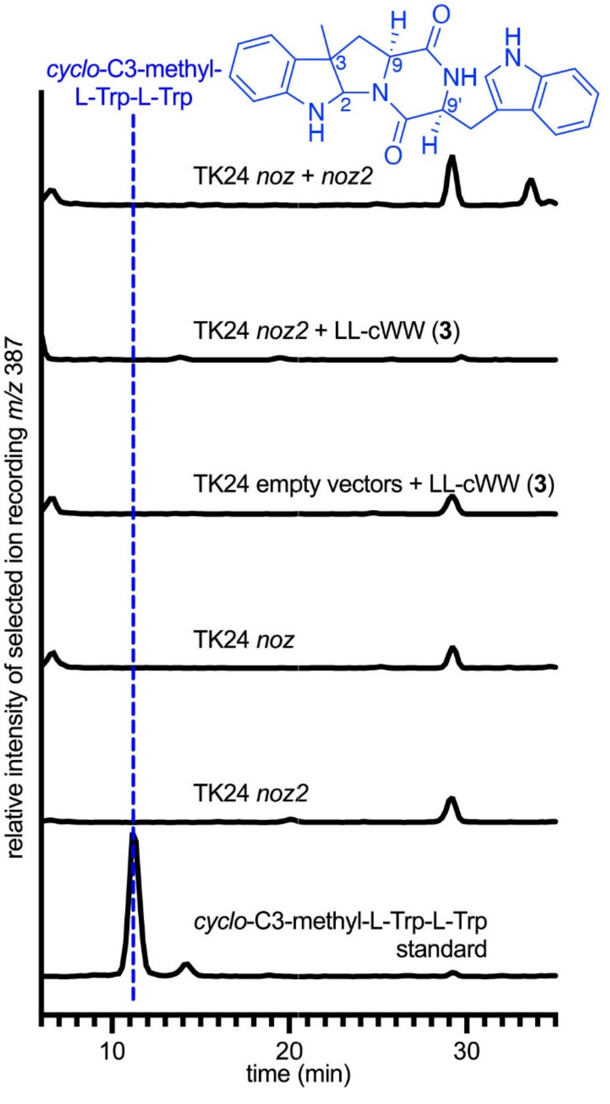

cyclo-C3-methyl-
L-Trp-L-Trp
TK24 noz + noz2
TK24 noz2 + LL-cWW (3)
TK24 empty vectors + LL-cWW (3)
TK24 noz
TK24 noz2
cyclo-C3-methyl-L-Trp-L-Trp
standard
relative intensity of selected ion recording m/z 387
10
20
30
time (min)

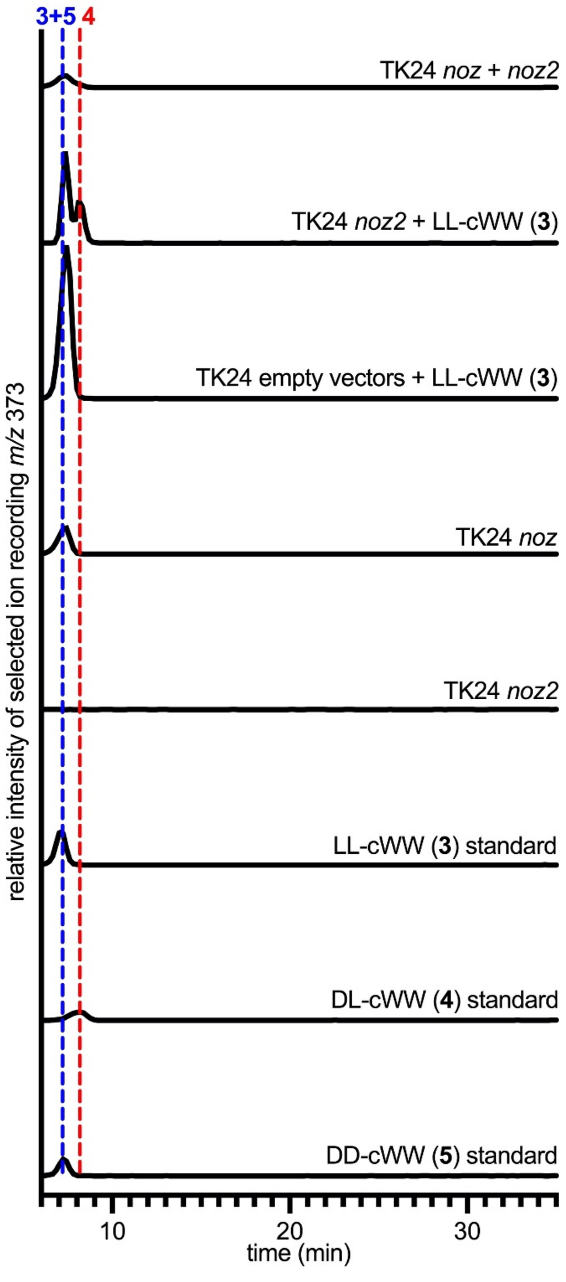

3+5
4
TK24 noz + noz2
TK24 noz2 + LL-cWW (3)
TK24 empty vectors + LL-cWW (3)
TK24 noz
TK24 noz2
LL-cWW (3) standard
DL-cWW (4) standard
DD-cWW (5) standard
relative intensity of selected ion recording m/z 373
10
20
30
time (min)

c)

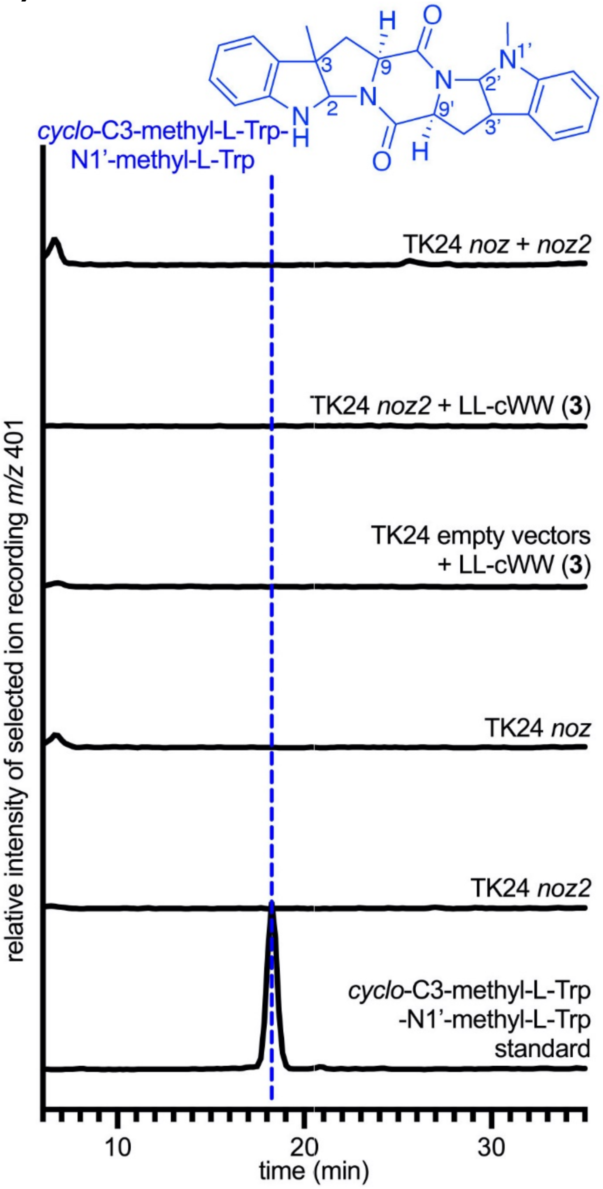
cyclo-C3-methyl-L-Trp-N1'-methyl-L-Trp
TK24 noz + noz2
TK24 noz2 + LL-cWW (3)
TK24 empty vectors + LL-cWW (3)
TK24 noz
TK24 noz2
cyclo-C3-methyl-L-Trp -N1'-methyl-L-Trp standard
relative intensity of selected ion recording m/z 401
10
20
30
time (min)

d)

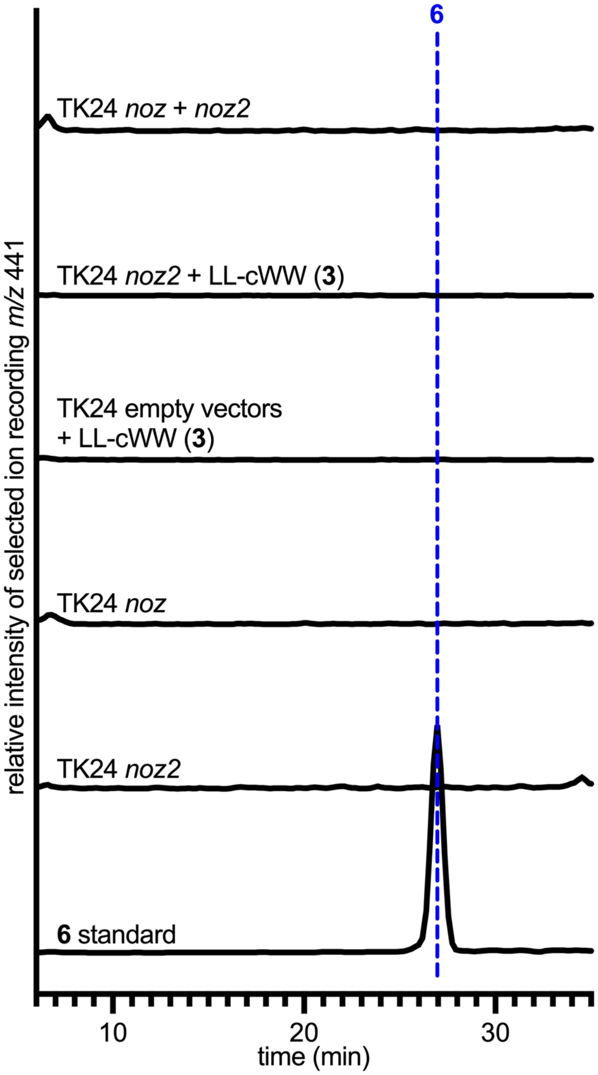
6
TK24 noz + noz2
TK24 noz2 + LL-cWW (3)
TK24 empty vectors + LL-cWW (3)
TK24 noz
TK24 noz2
6 standard
relative intensity of selected ion recording m/z 441
10
20
30
time (min)

e)

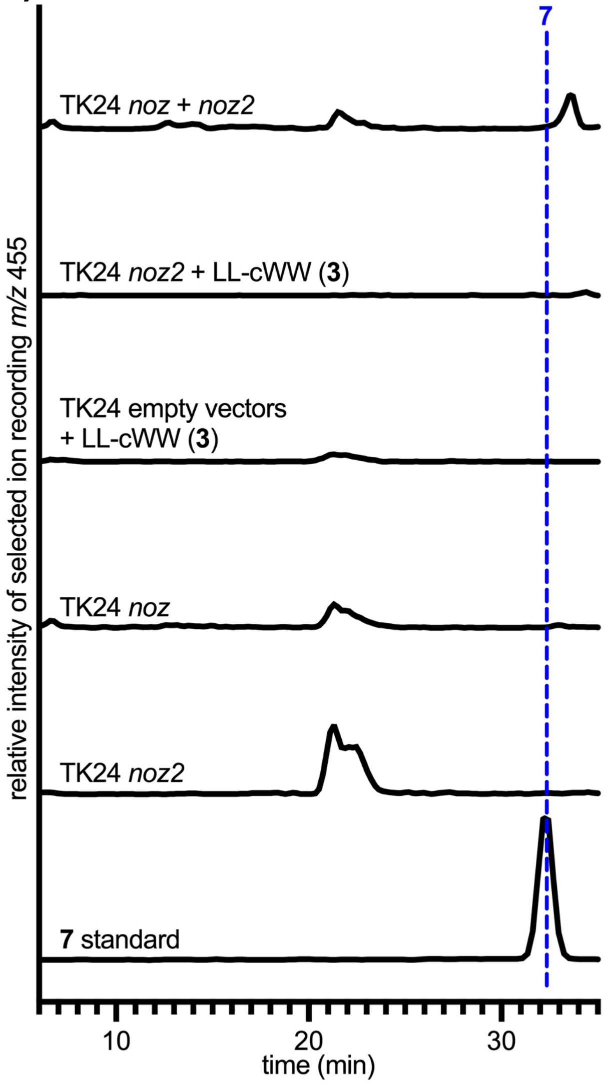

Supplementary Figure 3. LC-MS analyses of chemical extracts from *S. lividans* TK24 *noz* and/or *noz2* heterologous expression and biotransformation cultures for candidate nocardioazine B intermediates. LC-MS was conducted with analyte separation by a C_{18} stationary phase with H_2O/MeCN gradient (described in main text Methods) and detection by ESI$^+$. Plots show SIRs for **(a)** cWWs **3**-**5** ($[M+H]^+$ *m/z* 373), **(b)** methyl-cWW regioisomers including *cyclo*-C3-methyl-L-Trp-L-Trp synthetic standard ($[M+H]^+$ *m/z* 387), **(c)** dimethyl-cWW regioisomers including *cyclo*-C3-methyl-L-Trp-N1'-methyl-L-Trp synthetic standard ($[M+H]^+$ *m/z* 401), **(d)** prenylated cWW regioisomers including *cyclo*-D-Trp-C3'-prenyl-D-Trp (**6**) synthetic standard ($[M+H]^+$ *m/z* 441), and **(e)** monomethylated, prenylated cWW regioisomers including *cyclo*-D-Trp-N1'-methyl-C3'-prenyl-D-Trp (**7**) standard ($[M+H]^+$ *m/z* 455). Source data are provided as a Source Data file.

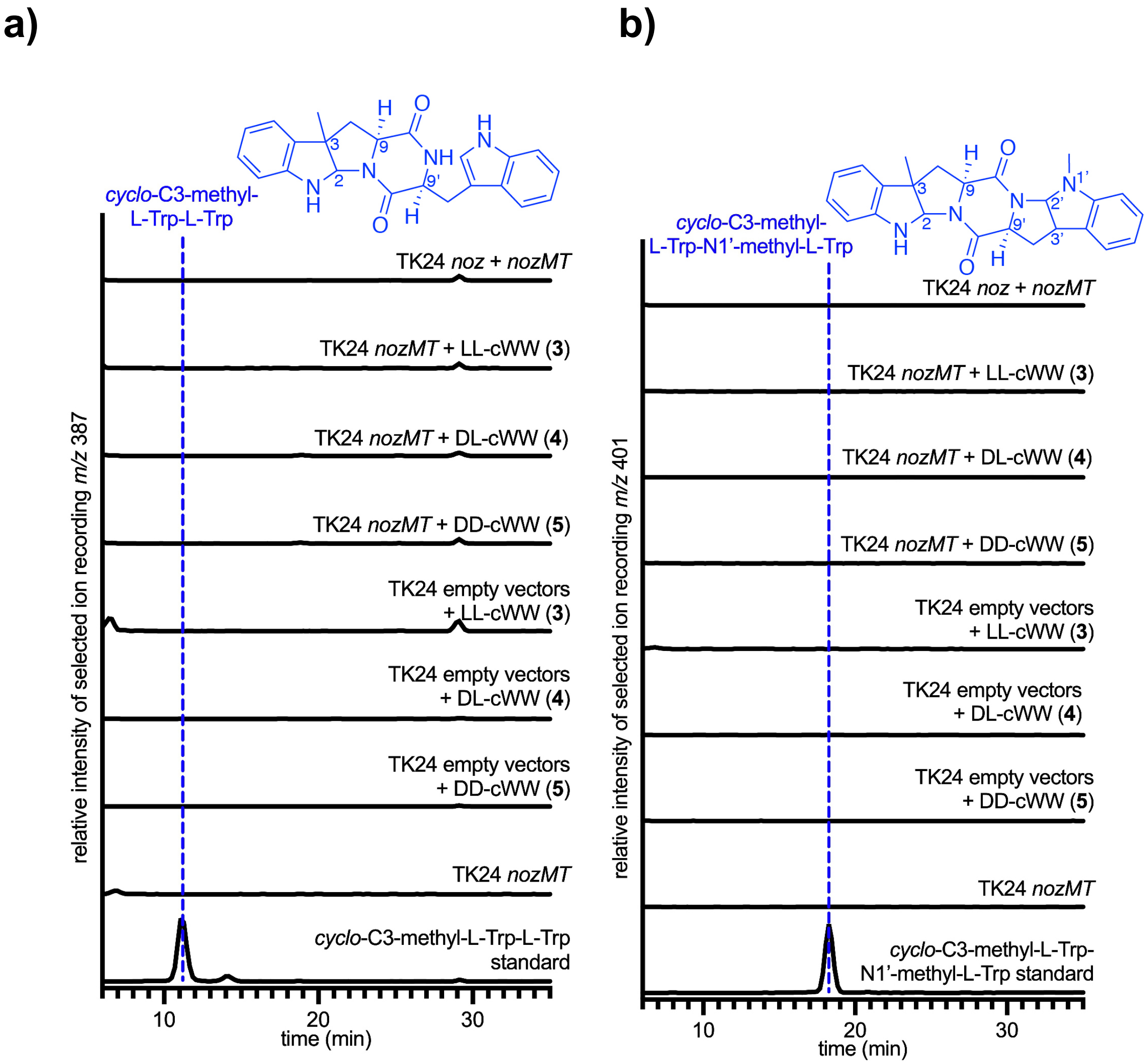

Supplementary Figure 4. LC-MS evaluation for candidate methylated nocardioazine intermediates resulting from chemical complementation of *S. lividans* TK24 *nozMT* with cWWs 3-5 or heterologous expression of *noz* (encoding NozA CDPS) with *nozMT* in *S. lividans* TK24. LC-MS was conducted with analyte separation by a C_{18} stationary phase with H_2O/MeCN gradient (described in main text Methods) and detection by ESI^+. **(a)** The $[M+H]^+$ *m/z* 387 SIR corresponds to monomethylated cWW candidates including *cyclo*-C3-methyl-L-Trp-L-Trp. **(b)** The $[M+H]^+$ *m/z* 401 SIR corresponds to dimethylated cWW candidates including *cyclo*-C3-methyl-L-Trp-N1'-methyl-L-Trp. Source data are provided as a Source Data file.

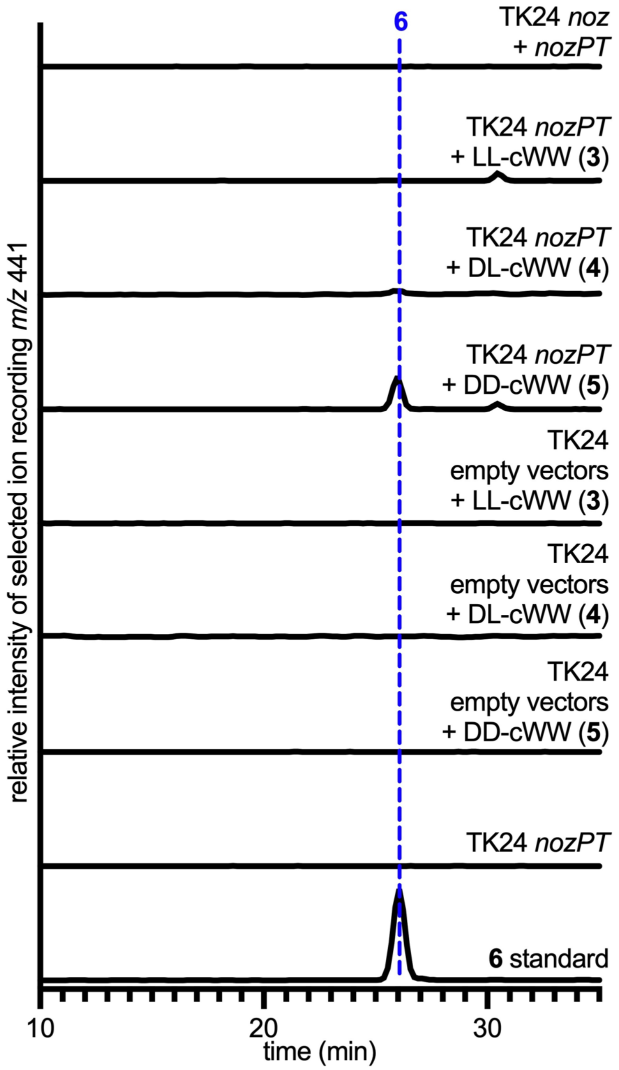

Supplementary Figure 5. LC-MS evaluation of candidate prenylated cWW intermediates resulting from chemical complementation of *S. lividans* TK24 *nozPT* with cWWs 3-5 or heterologous expression of *noz* (encoding NozA CDPS) with *nozPT* in TK24. LC-MS was conducted with analyte separation by a C_{18} stationary phase with H_2O/MeCN gradient (described in main text Methods) and detection by ESI^+. The $[M+H]^+$ *m/z* 441 SIR corresponds to prenylated cWW candidates including *cyclo*-D-Trp-C3'-prenyl-D-Trp (**6**) and regio- or stereoisomers. Source data are provided as a Source Data file.

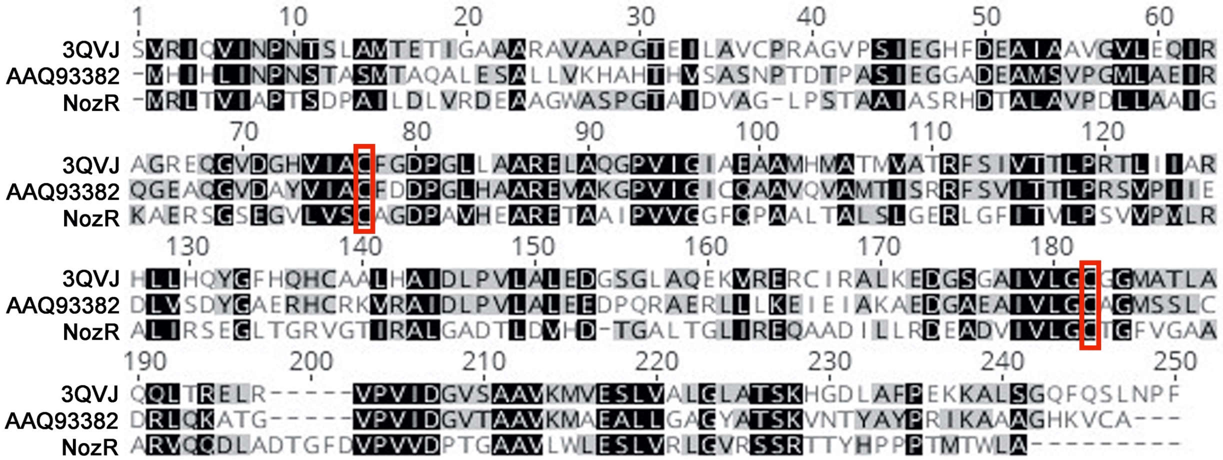

Supplementary Figure 6. Alignment of NozR amino acid sequence with selected structurally characterized allantoin racemase sequences (GenBank accession #3QVJ and #AAQ93382). Alignment of NozR with these other racemases indicated conservation of an acid-base catalytic cysteine dyad (red boxes). Amino acid residues are colored according to similarity (black = most similar; white = most dissimilar).

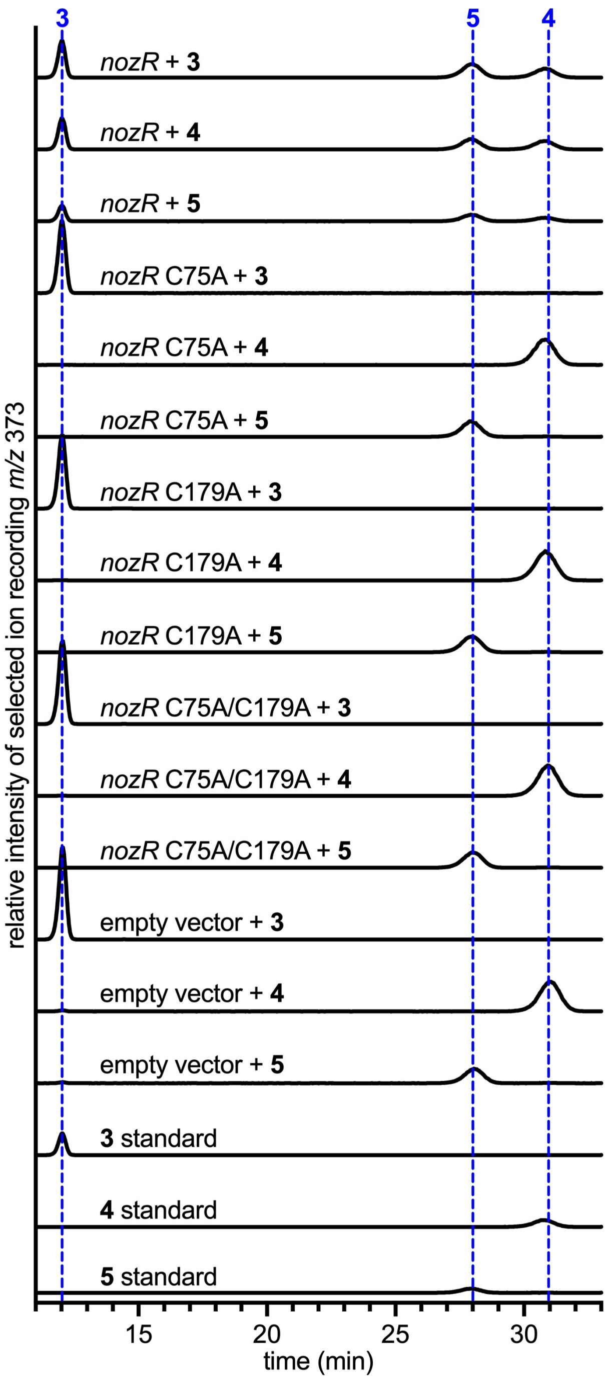

Supplementary Figure 7. *In vivo* biotransformation experiments support the hypothesized NozR stereoisomerase catalytic dyad of Cys-75 and Cys-179. Chemical complementation of *S. lividans* TK24 pUWL201-*nozR* transformants with individual cWW isomers **3**, **4**, or **5** resulted in observation of all three cWW stereoisomers. In contrast, execution of these complementation experiments using *S. lividans* TK24 transformants with pUWL201-*nozR* constructs encoding NozR C75A, C179A, or C75A/C179A mutants resulted in no detectable interconversion of stereoisomers by LC-MS with a chiral cellulose column and isocratic mobile phase of 50/50 H_2O/MeCN (details provided in main text Methods). Source data are provided as a Source Data file.

Supplementary Table 2. Summary of ^{1}H, ^{13}C, HMBC, COSY, and ROESY NMR spectral data for biosynthetic *cyclo*-D-Trp-C3'-prenyl-D-Trp (6) in $CDCl_3$. Direct connections between carbon and hydrogen atoms were assigned from HSQC spectra. Signals are referenced to residual chloroform signal at 77.16 ppm for ^{13}C and 7.24 ppm for ^{1}H. Source data are provided as a Source Data file.

position	δ ^{1}H, mult. ($J_{H,H}$ in Hz), integral	δ ^{13}C	^{1}H-^{13}C HMBC	COSY	ROESY
1 (NH)	8.11, s, 1H	-	-	2	-
2	7.09, s, 1H	123.4	3, 7a	1	-
3	-	109.9	-	-	-
3a	-	126.7	-	-	-
4	7.54, dm (*8.0*), 1H	118.6	3, 6, 7a	5	5
5	7.11, dm (*7.5*), 1H	120.3	3a, 7	4, 6	4
6	7.21, tm (*7.6*), 1H	123.1	4, 7a	5, 7	7
7	7.37, dt (*8.1, 0.8*), 1H	111.7	3a, 5	6	6
7a	-	136.8	-	-	-
8a	2.96, dd (*15.2, 10.8*), 1H	27.1	2, 3, 3a, 9	8b, 9	8b, 9
8b	3.73, dd (*15.1, 3.7*), 1H		-	8a, 9	8a, 9
9	4.31, dm (*10.7*), 1H	54.7	-	8a, 8b	8a, 8b, NH
10	-	166.4	-	-	-
2'	5.32, d (*3.9*), 1H	79.7	1", 3'a, 7'a, 9'	-	1"
3'	-	55.8	-	-	-
3'a	-	131.3	-	-	-
4'	7.05, brd (*7.9*), 1H	123.5	3', 6', 7'a	5'	5', 8'b
5'	6.75, m, 1H	119.5	3'a, 7'	4', 6'	4', 6'
6'	7.08, m, 1H	128.9	4', 7'a	5', 7'	5', 7'
7'	6.62, t (*7.6*), 1H	109.6	3'a, 5'	6'	6'
7'a	-	149.0	-	-	-
8'a	2.22, dd (*13.2, 11.3*), 1H	38.1	1", 3', 3'a, 9', 10'	8'b, 9	1", 8'b
8'b	2.58, dd (*12.5, 5.9*), 1H		2', 3', 3'a, 9'	8'a, 9	4', 8'a, 9'
9'	3.96, m, 1H	59.2	-	8'a, 8'b	8'b
10'	-	169.2	-	-	-
1"	2.34-2.37, m, 2H	35.5	2', 2", 3', 3'a, 3", 8'	2"	2', 2", 8'a, 5"
2"	5.13, tm (*7.4*), 1H	118.5	4", 5"	1"	1", 4"
3"	-	136.0	-	-	-
4"	1.69, s, 1H	26.2	2", 3", 5"	-	2"
5"	1.52, s, 1H	18.1	2", 3", 4"	-	1"
NH	5.65, s, 1H	-	9', 10	-	9

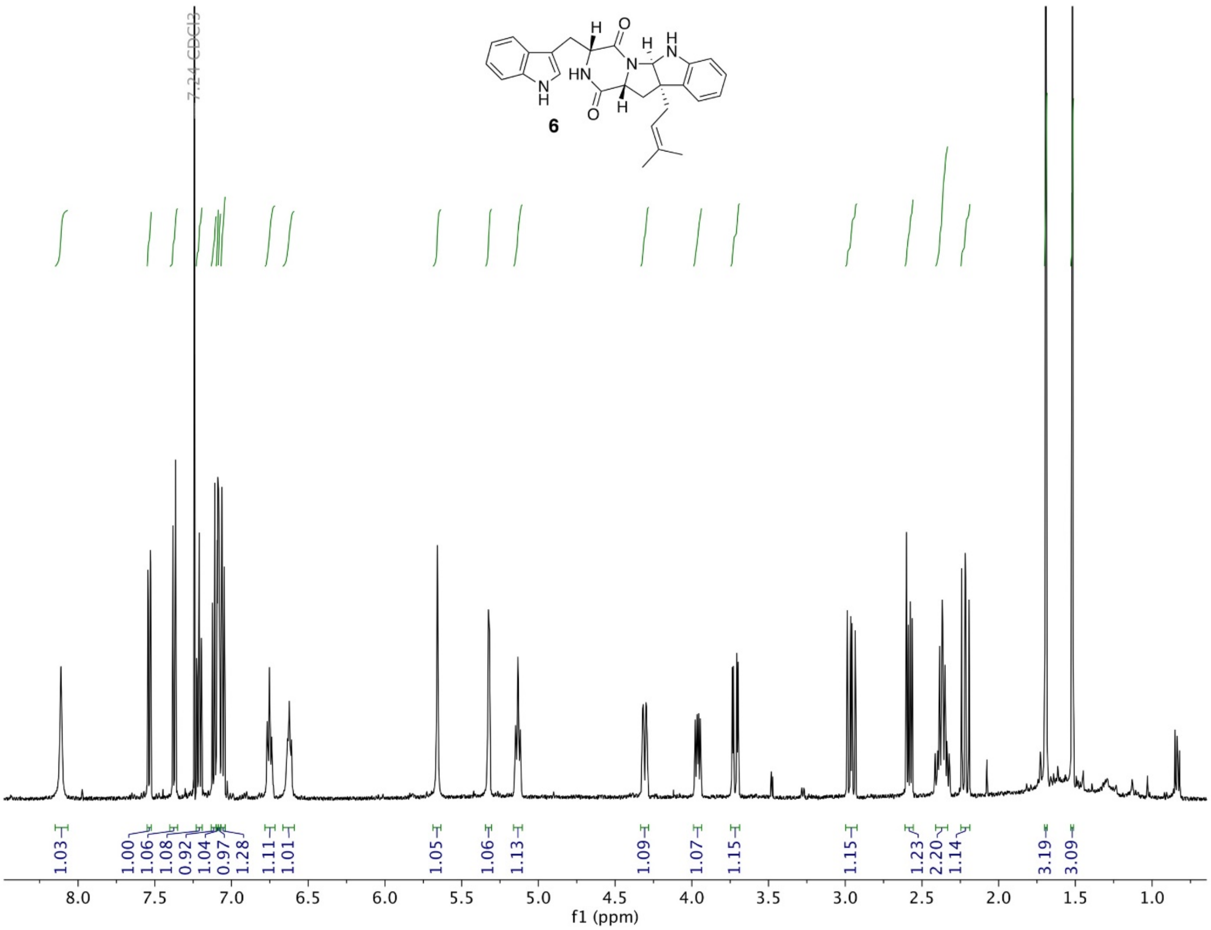

Supplementary Figure 8. ^{1}H NMR spectrum of biosynthetic *cyclo*-D-Trp-C3'-prenyl-D-Trp (**6**) (500 MHz; $CDCl_3$). Source data are provided as a Source Data file.

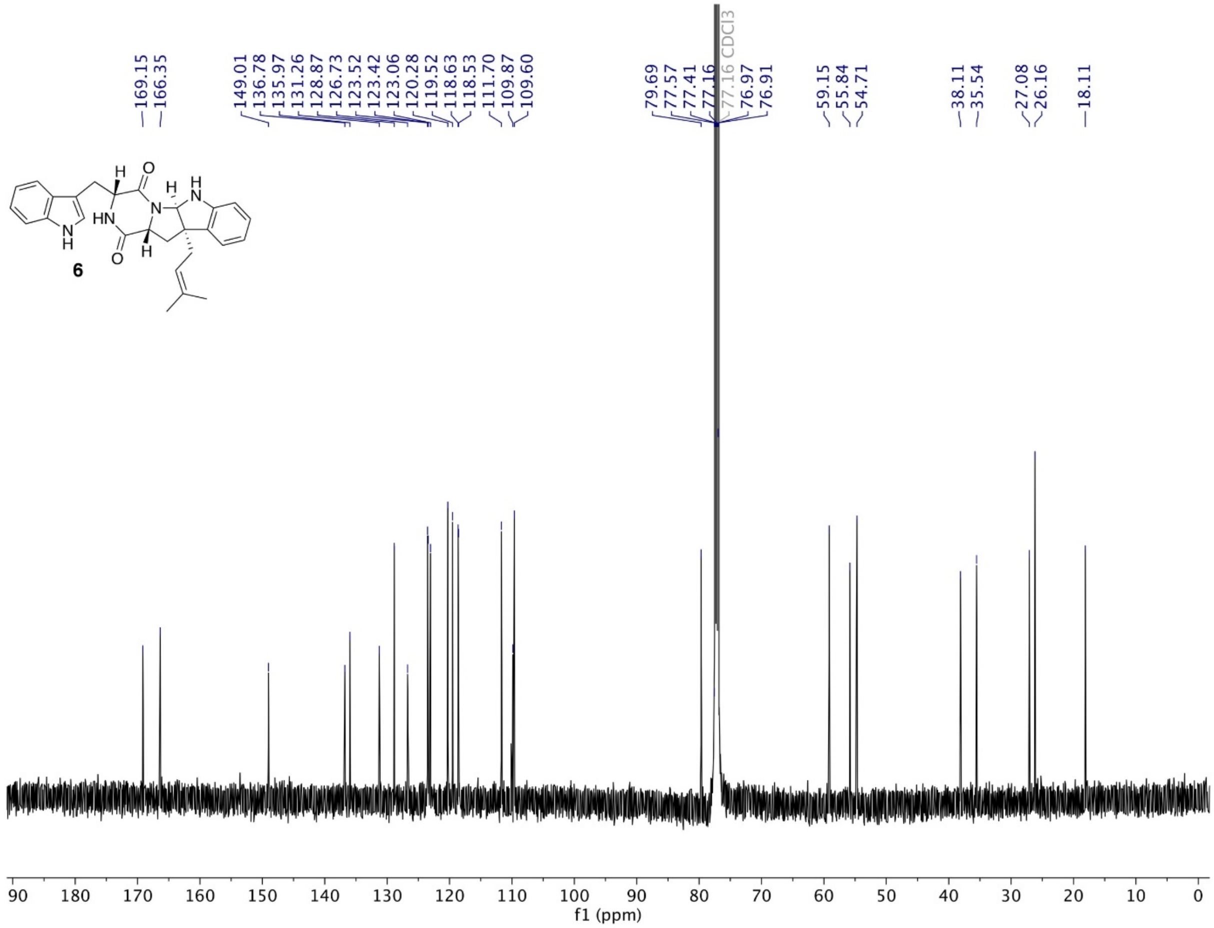

Supplementary Figure 9. ^{13}C NMR spectrum of biosynthetic *cyclo*-D-Trp-C3'-prenyl-D-Trp (**6**) (125 MHz; $CDCl_3$). Source data are provided as a Source Data file.

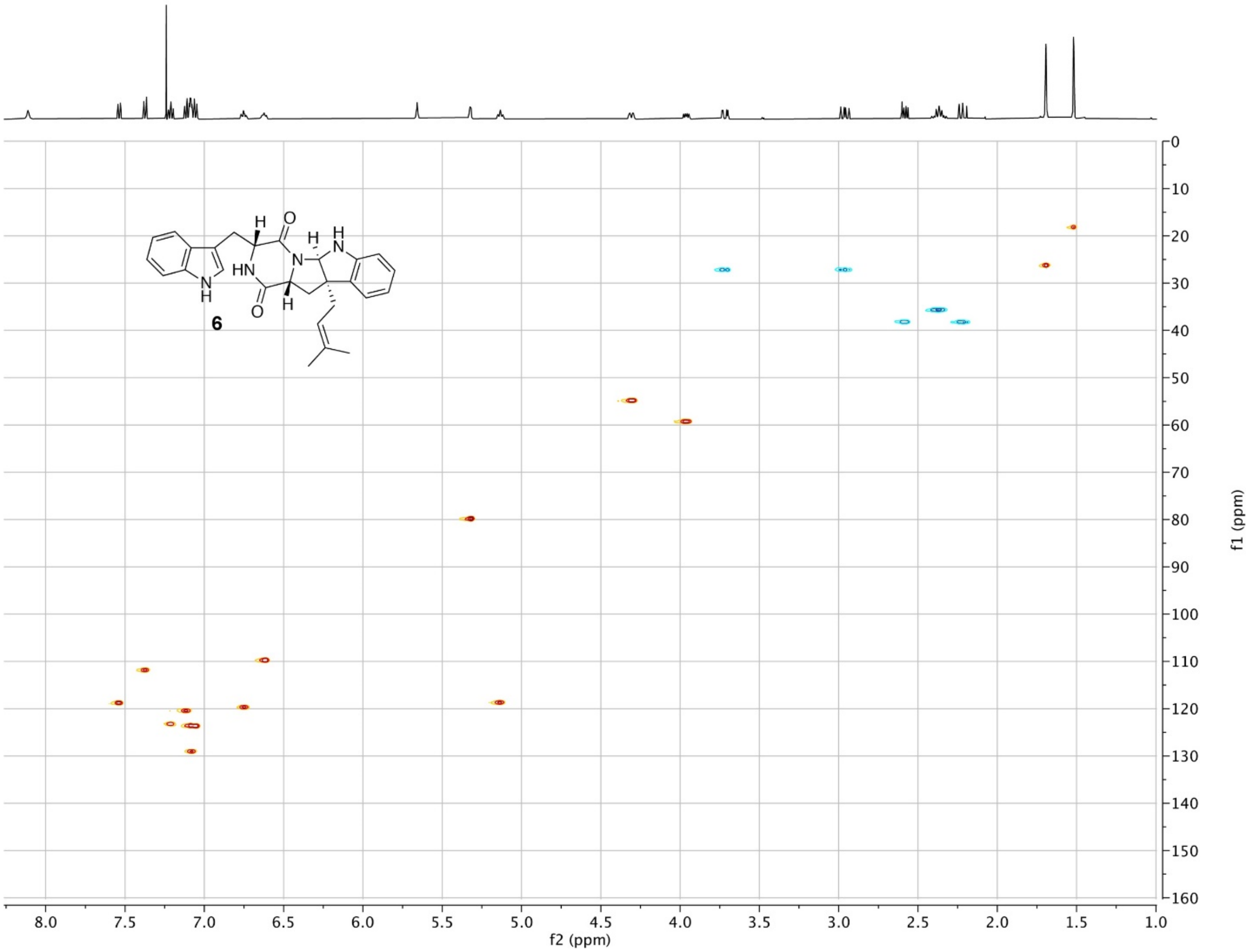

Supplementary Figure 10. HSQC spectrum of biosynthetic *cyclo*-D-Trp-C3'-prenyl-D-Trp (**6**) in $CDCl_3$. Source data are provided as a Source Data file.

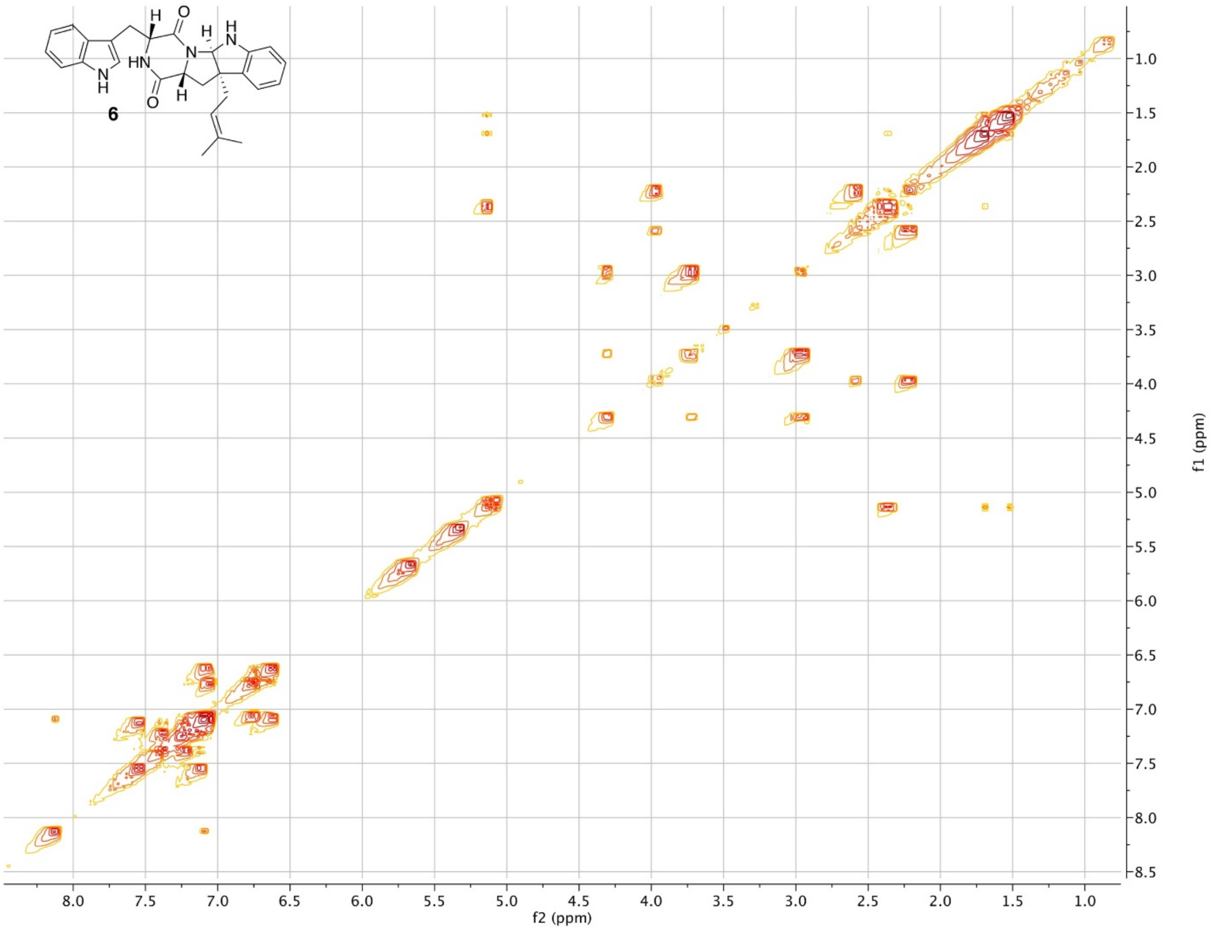

Supplementary Figure 11. COSY spectrum of biosynthetic *cyclo*-D-Trp-C3'-prenyl-D-Trp (**6**) in $CDCl_3$. Source data are provided as a Source Data file.

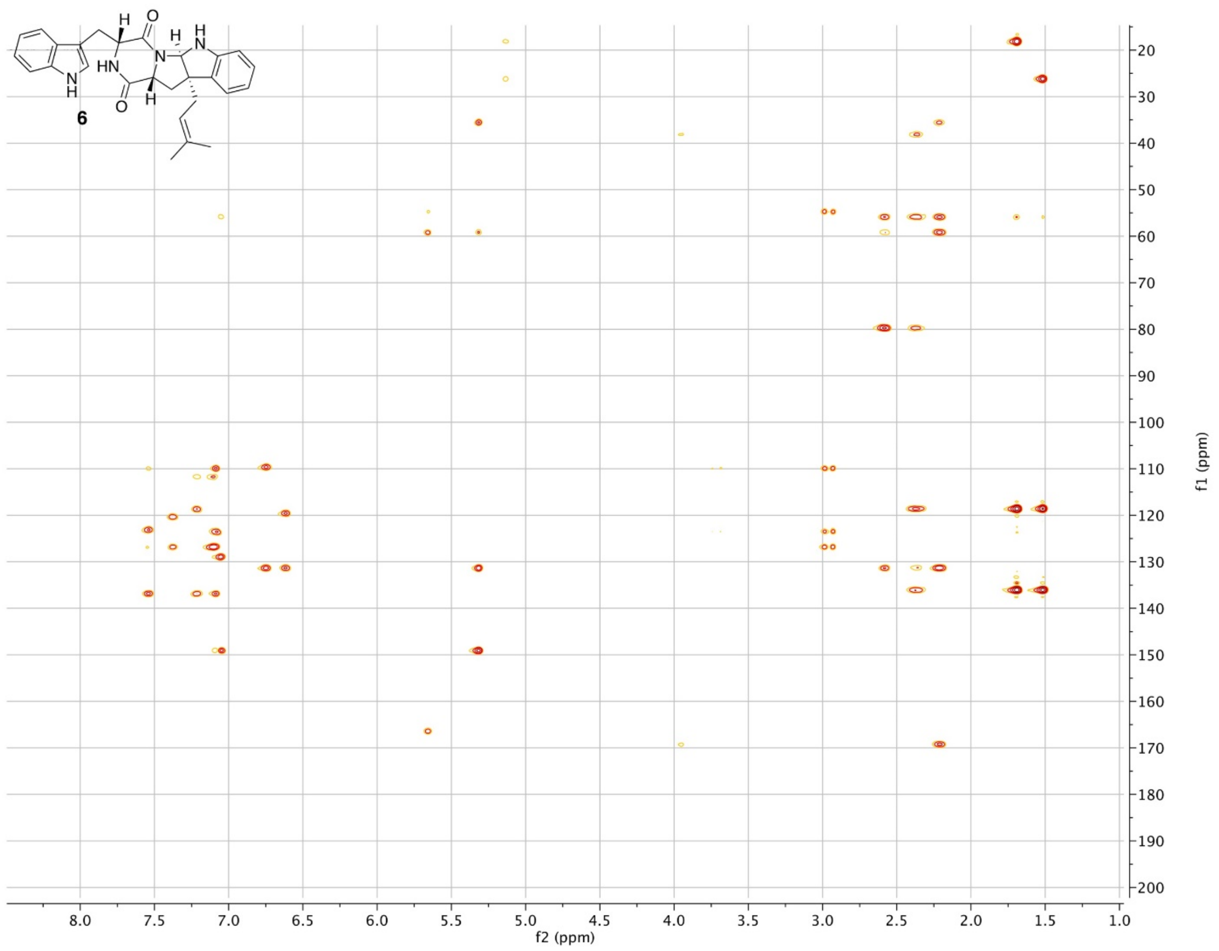

Supplementary Figure 12. HMBC spectrum of biosynthetic *cyclo*-D-Trp-C3'-prenyl-D-Trp (**6**) in $CDCl_3$. Source data are provided as a Source Data file.

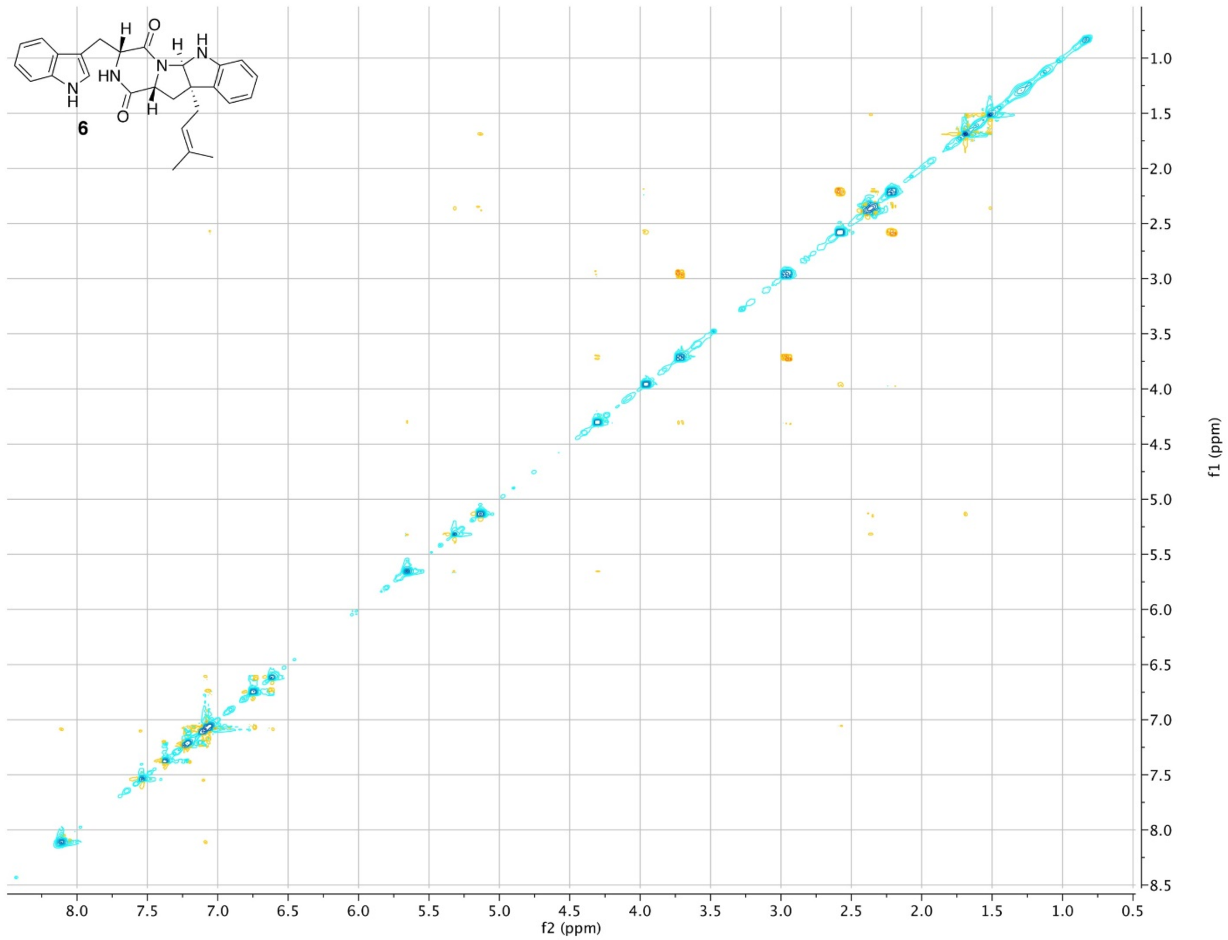

Supplementary Figure 13. ROESY spectrum of biosynthetic *cyclo*-D-Trp-C3'-prenyl-D-Trp (**6**) in $CDCl_3$. Source data are provided as a Source Data file.

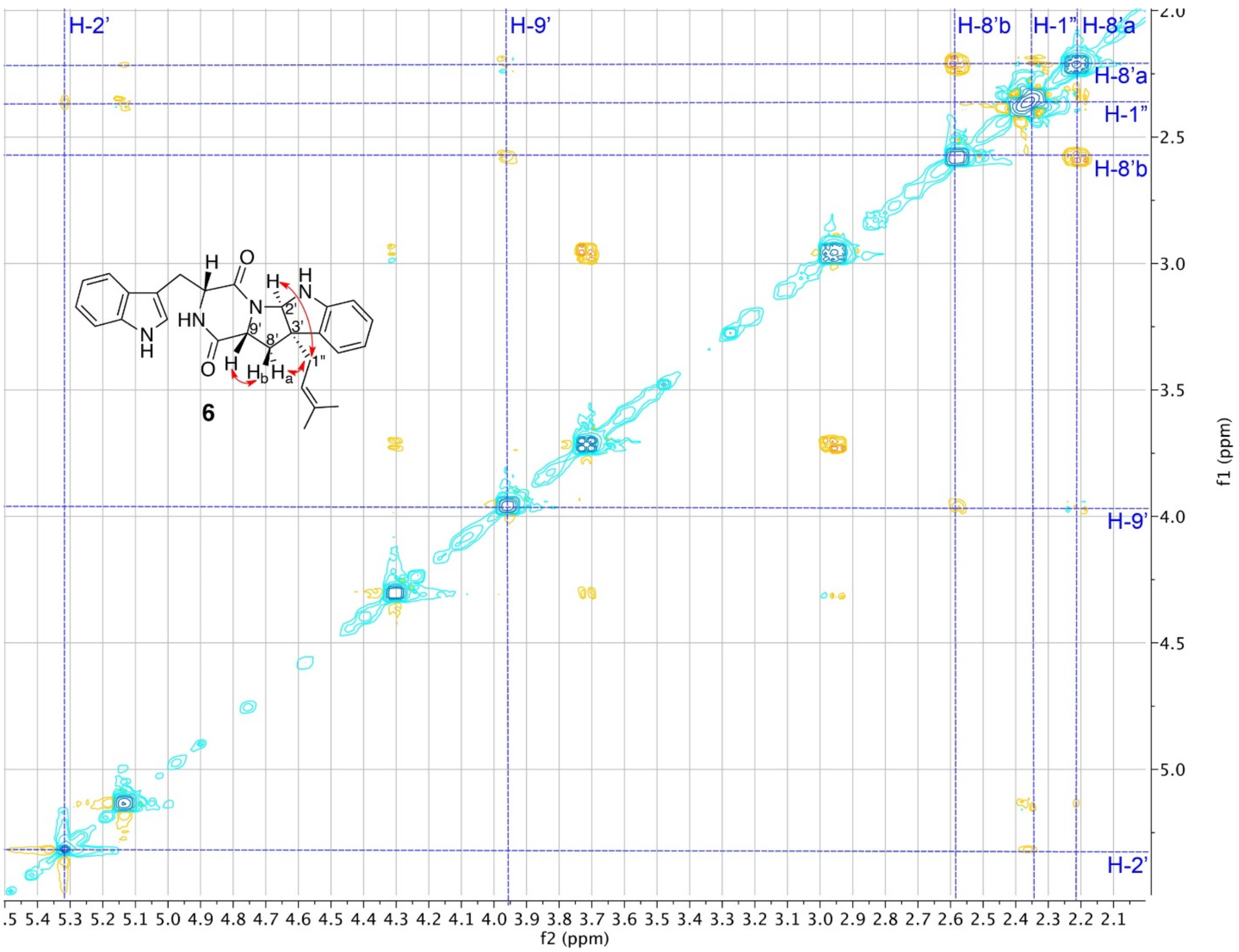

Supplementary Figure 14. Enlargement of region of ROESY spectrum from Fig. S13 for biosynthetic *cyclo*-D-Trp-C3'-prenyl-D-Trp (**6**) that shows key correlations for relative stereochemistry determination. Source data are provided as a Source Data file.

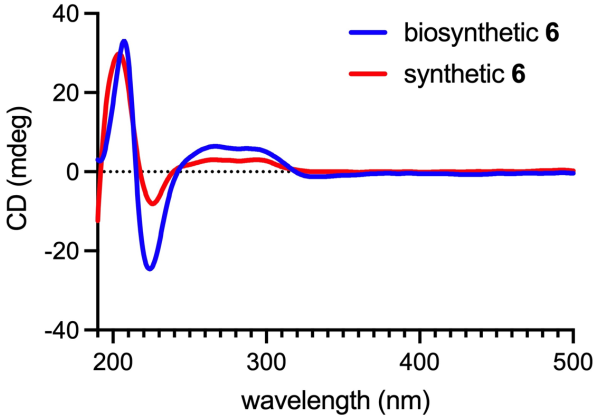

Supplementary Figure 15. Comparison of ECD spectra for biosynthetic and synthetic *cyclo*-D-Trp-*C*3'-prenyl-D-Trp (6). Spectra were collected at an analyte concentration of ~100μM in methanol. Source data are provided as a Source Data file.

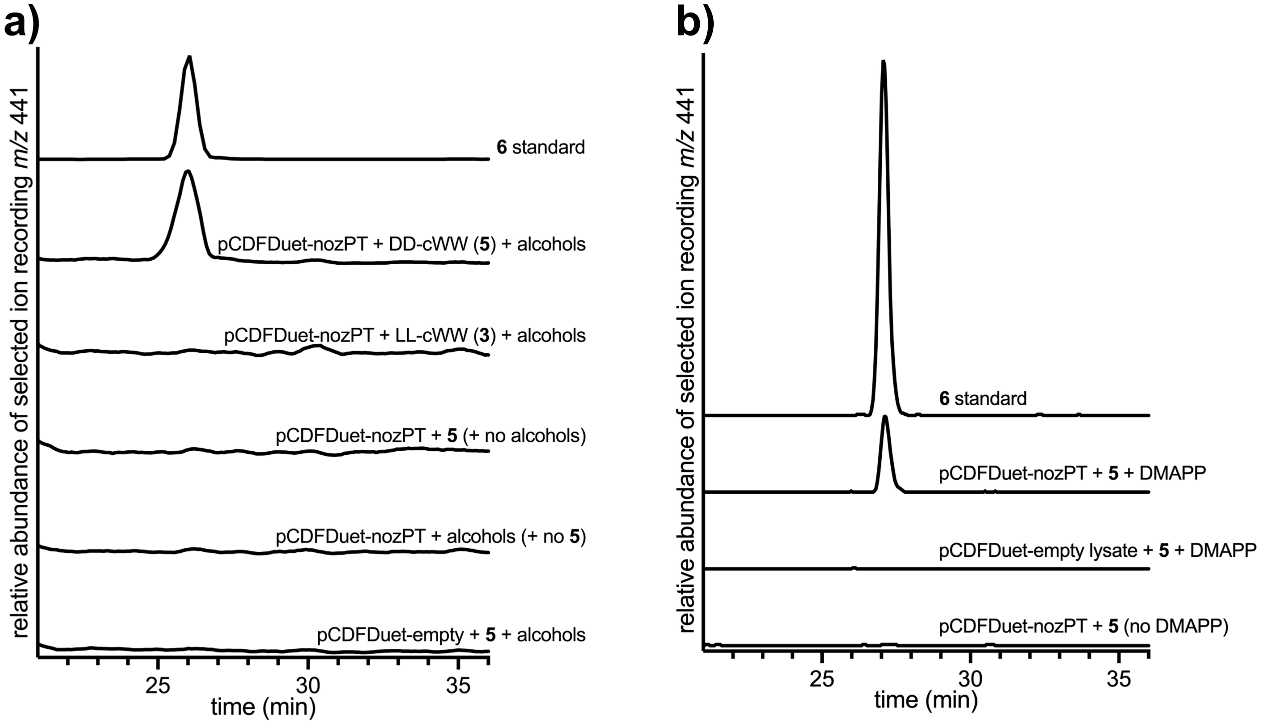

Supplementary Figure 16. LC-MS evaluation of *cyclo*-D-Trp-C3'-prenyl-D-Trp (6) production by *E. coli* NozPT heterologous expression systems. (a) Evaluation of **6** production by chemical complementation of *E. coli* BL21(DE3) pCDFDuet-nozPT pETDuet-PhoN-IPK cultures. Cultures were supplemented with alcohols 3-methylbut-2-en-1-ol and 3-methylbut-3-en-1-ol, as dimethylallyl pyrophosphate (DMAPP) and isopentenyl pyrophosphate (IPP) precursors, and either LL-cWW (**3**) or DD-cWW (**5**). **(b)** For *in vitro* experiments, **5** was incubated with DMAPP and cell lysates from *E. coli* BL21(DE3) pCDFDuet-nozPT to yield **6**. LC-MS was conducted with analyte separation by a C_{18} stationary phase with H_2O/MeCN gradient (described in main text Methods) and detection by ESI^+. Plots show SIR *m/z* 441 for the $[M+H]^+$ of **6** or other prenylated isomers. Source data are provided as a Source Data file.

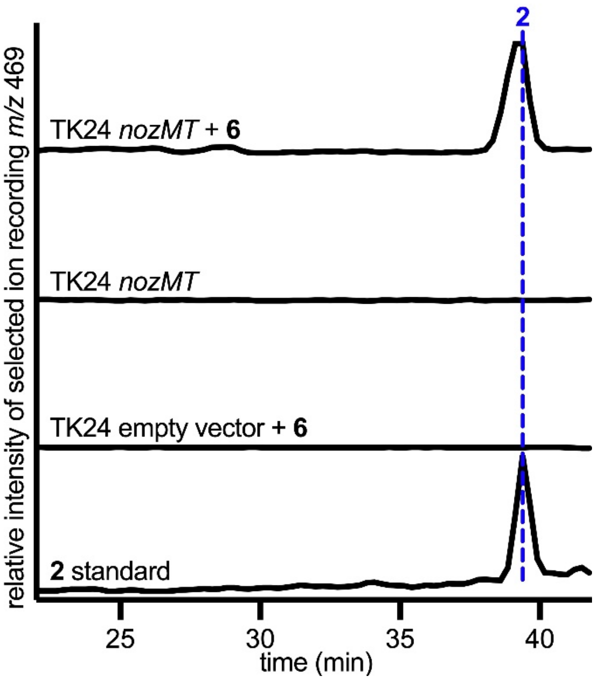

Supplementary Figure 17. LC-MS evaluation of nocardioazine B production from chemical complementation of *S. lividans* TK24 *nozMT* transformants with prenylated cWW 6. LC-MS was conducted with analyte separation by a C_{18} stationary phase with H_2O/MeCN gradient (described in main text Methods) and detection by ESI^+. Plots show SIR for *m/z* 469 corresponding to the $[M+H]^+$ of nocardioazine B (**2**). The trace for **2** standard from *Nocardiopsis* sp. CMB-M0232 is also shown. Source data are provided as a Source Data file.

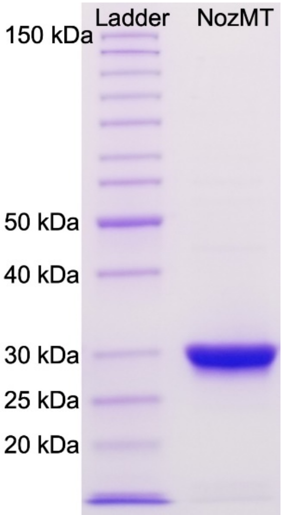

Supplementary Figure 18. SDS-PAGE analysis of recombinant N-terminal hexahistidine-tagged NozMT following purification by Ni affinity chromatography. Protein ladder is shown in lane 1, and lane 2 shows pooled fractions used for enzyme assays of NozMT. This result is representative of independent replication of this experiment three times. The theoretical molecular weight of hexahistidine-tagged NozMT is 30.2 kDa. Source data are provided as a Source Data file.

a)

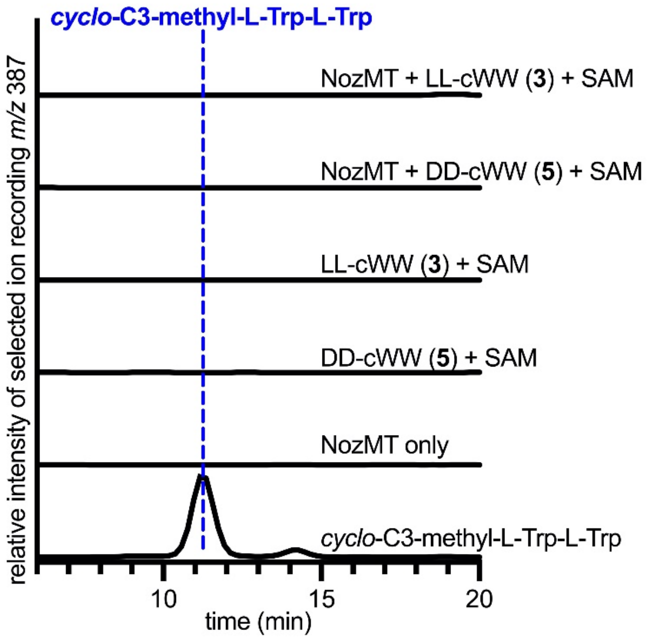

b)

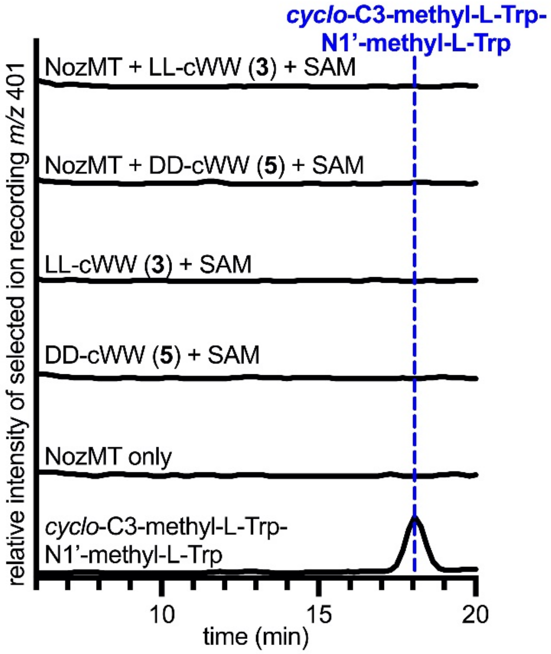

Supplementary Figure 19. LC-MS evaluation of cWW stereoisomers as NozMT substrates for *in vitro* methylation reactions. Incubation of purified recombinant NozMT with LL-cWW (**3**) or DD-cWW (**5**) along with *S*-adenosylmethionine (SAM) yielded no detectable candidate **(a)** monomethylated cWW ($[M+H]^+$ *m/z* 387) or **(b)** dimethylated cWW ($[M+H]^+$ *m/z* 401) derivatives. Traces for selected synthetic standards are also shown. LC-MS was conducted with analyte separation by a C_{18} stationary phase with H_2O/MeCN gradient (described in main text Methods) and detection by ESI^+. Source data are provided as a Source Data file.

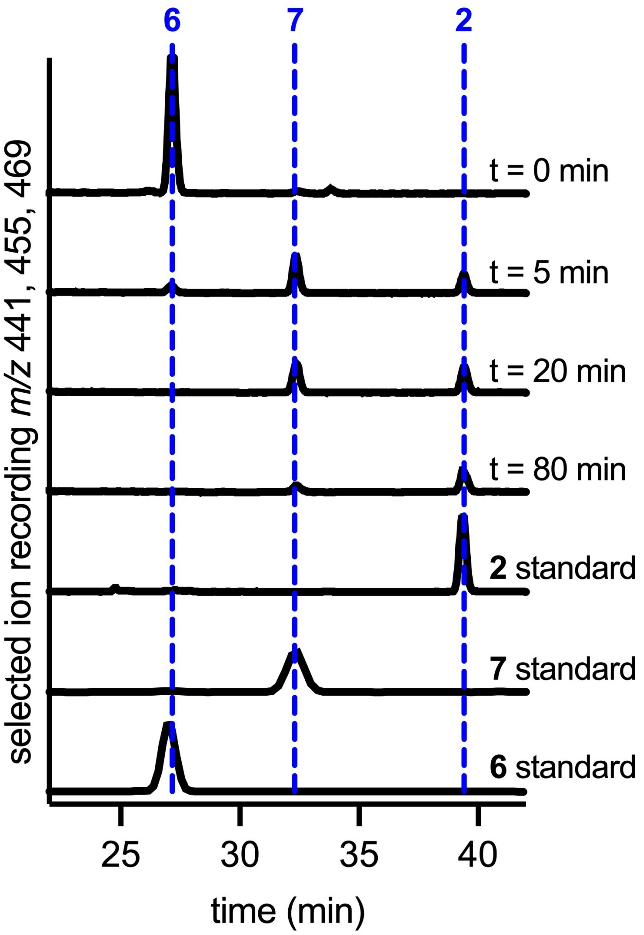

Supplementary Figure 20. LC-MS evaluation of intermediates and products from *in vitro* NozMT reactions with prenylated 6 substrate at selected time points. Reaction mixtures contained NozMT, reactant *cyclo*-D-Trp-C3'-prenyl-D-Trp (**6**), and SAM. LC-MS was conducted with analyte separation by a C_{18} stationary phase with H_2O/MeCN gradient (described in main text Methods) and detection by ESI^+. SIR data were collected for $[M+H]^+$ *m/z* 441 of reactant **6**, $[M+H]^+$ *m/z* 455 of hypothesized *N*1'- or *C*3-monomethylated intermediates including observed intermediate *cyclo*-D-Trp-N1'-methyl-C3'-prenyl-D-Trp (**7**), and $[M+H]^+$ *m/z* 469 of nocardioazine B (**2**) product. Source data are provided as a Source Data file.

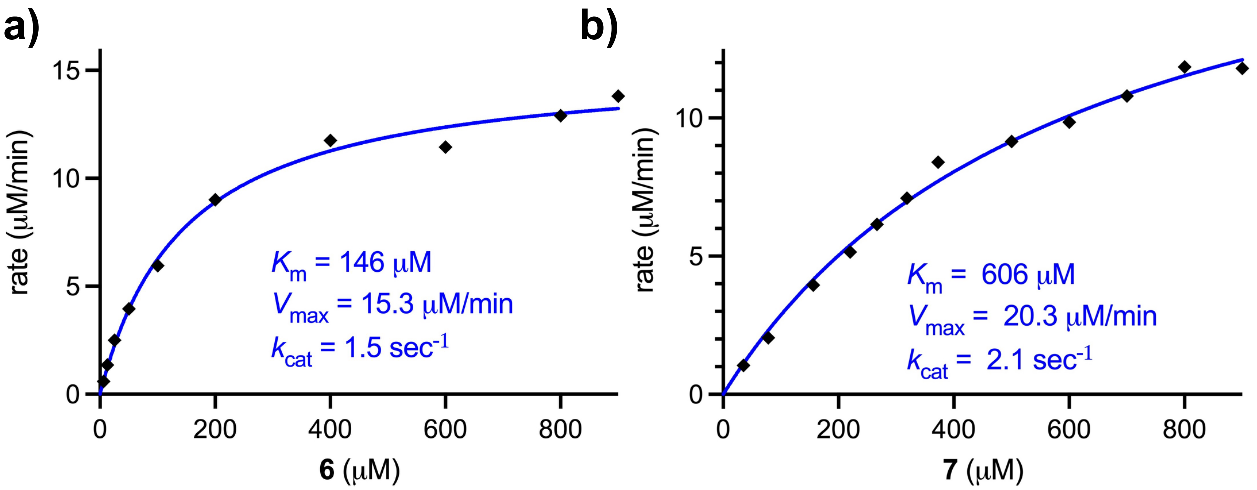

Supplementary Figure 21. Kinetics characterization of NozMT for methylation of substrates *cyclo*-D-Trp-C3'-prenyl-D-Trp (6) and *cyclo*-D-Trp-N1'-methyl-C3'-prenyl-D-Trp (7). Plots of initial velocity (v_o) versus concentration of substrates **(a) 6** and **(b) 7** were used to evaluate Michaelis-Menten kinetics parameters. K_m, V_{max}, and k_{cat} values and individual plotted data points indicate mean for n = 2 independent replicates. Source data are provided as a Source Data file.

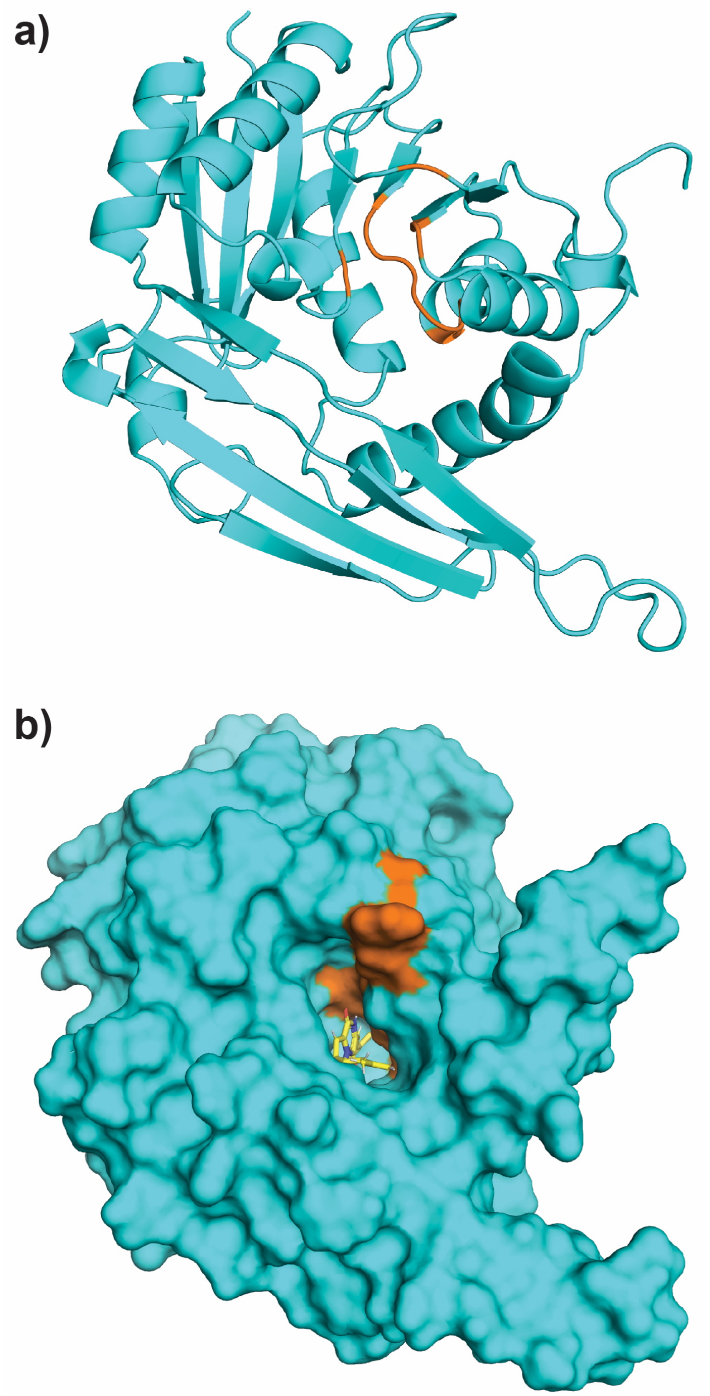

Supplementary Figure 22. Homology model of dual function *N*1'- and *C*3-methyltransferase NozMT and docked substrate *cyclo*-D-Trp-C3'-prenyl-D-Trp (6). **(a)** This model supports that NozMT shares features typical of class I methyltransferases, including a Rossmann-like fold of beta strands enveloped by alpha helices. In this ribbons diagram, SAM binding site residues conserved among class I methyltransferases are shown in orange and other residues in cyan. **(b)** The SAM binding site (orange) is proximal to docked substrate **6** (yellow sticks), with NozMT rendered as a Connolly surface in cyan except for the orange SAM binding site. Supplementary Files 2-3 provide PDB files of docking structures.

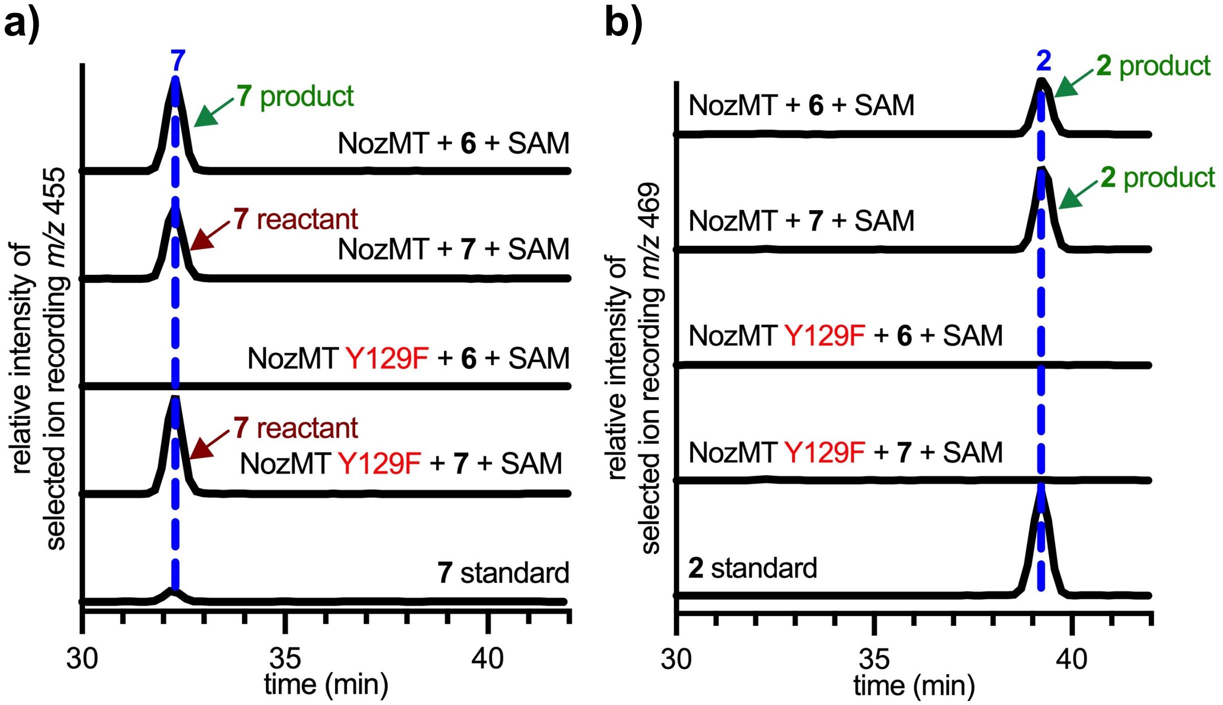

Supplementary Figure 23. ***In vitro*** **evaluation of NozMT Y129F mutant for catalysis of methylation of** ***cyclo*****-D-Trp-C3'-prenyl-D-Trp (6) or** ***cyclo*****-D-Trp-N1'-methyl-C3'-prenyl-D-Trp (7).** Incubation of purified recombinant NozMT Y129F with **6** or **7** along with SAM yielded no detectable candidate products corresponding to methylation catalysis, while incubation of wild-type NozMT with substrates **6**-**7** and SAM resulted in accumulation of methylated products detected by LC/MS. LC-MS was conducted with analyte separation by a C_{18} stationary phase with H_2O/MeCN gradient (described in main text Methods) and detection by ESI^+. Plots show SIRs for **(a)** **7** (with $[M+H]^+$ *m/z* 455) and **(b)** nocardioazine B (**2**, with $[M+H]^+$ *m/z* 469). Traces for synthetic **2** and **7** standards are also shown. Source data are provided as a Source Data file.

Supplementary Table 3. Sequences of oligonucleotides used for PCR amplification of *noz2* cluster genes for cloning with pUWL201. Nucleotides corresponding to gene(s) from the *noz2* genomic locus are capitalized, while those corresponding to the pUWL201 sequence are lower case.

primer name	purpose	sequence (5'-3')	amplicon size (kB)
noz2_F2<br>noz2_R2	cloning of entire *noz2* cluster using pUWL201	gttggtaggatcgacggtatcgataCGTGGGCCCGGAAGGAAC<br>taatgcagagcttctagaactagtgTCAGGACGGTGCGACCATG	2.7
MT_F<br>MT_R	cloning of *nozMT* using pUWL201	taggatcgacggtatcgataAACGAACAGGTGAGTGAACGAC<br>cagagcttctagaactagtgTCAGGACGGTGCGACCATG	0.84
PT_F<br>PT_R	cloning of *nozPT* using pUWL201	taggatcgacggtatcgataCGTGGGCCCGGAAGGAACGC<br>cagagcttctagaactagtgTCAGCGCGGCCCGACGGG	1.0
racemase_F<br>racemase_R	cloning of *nozR* using pUWL201	taggatcgacggtatcgataCCGAATCGAACCGACGGG<br>cagagcttctagaactagtgTCAGGCGAGCCACGTCATC	0.75

Supplementary Table 4. Sequences of oligonucleotides for site-directed mutagenesis of *nozR* in pUWL201 using Agilent Quikchange XL II kit. Nucleotides encoding the cysteine to alanine mutation (TGC → GCC) are underlined/bold; all other nucleotides correspond to the wild-type *nozR* gene.

primer name	purpose	sequence (5'-3')
nozRC75A_F<br>nozRC75A_R	creation of pUWL201 construct encoding NozR C75A mutant	GGCGTGCTGGTCTCC**<u>GCC</u>**GCGGGCGACCCCGC<br>GCGGGGTCGCCCGC**<u>GGC</u>**GGAGACCAGCACGCC
nozRC179A_F<br>nozRC179A_R	creation of pUWL201 construct encoding NozR C179A mutant	GTCATCGTGCTGGGC**<u>GCC</u>**ACGGGCTTCGTGGG<br>CCCACGAAGCCCGT**<u>GGC</u>**GCCCAGCACGATGAC

Supplementary Table 5. Sequences of oligonucleotides for site-directed mutagenesis of *nozMT* synthetic gene in pQE31 using Agilent Quikchange XL II kit. Nucleotides encoding the tyrosine to phenylalanine mutation (TAT → TTT) are underlined/bold; all other nucleotides correspond to the unmutated synthetic *nozMT* gene sequence.

primer name	purpose	sequence (5'-3')
nozMT_Y22F_F<br>nozMT_Y22F_R	creation of pQE31 construct encoding NozMT Y22F mutant	GCAACTATCGCCGACTCG**<u>TTT</u>**GATCGTCTTTTGG<br>CCAAAAGACGATC**<u>AAA</u>**CGAGTCGGCGATAGTTGC
nozMT_Y129F_F<br>nozMT_Y129F_R	creation of pQE31 construct encoding NozMT Y129F mutant	GGAACGGGACTCAAC**<u>TTT</u>**CTGGCCTCACCGGACG<br>CGTCCGGTGAGGCCAG**<u>AAA</u>**GTTGAGTCCCGTTCC

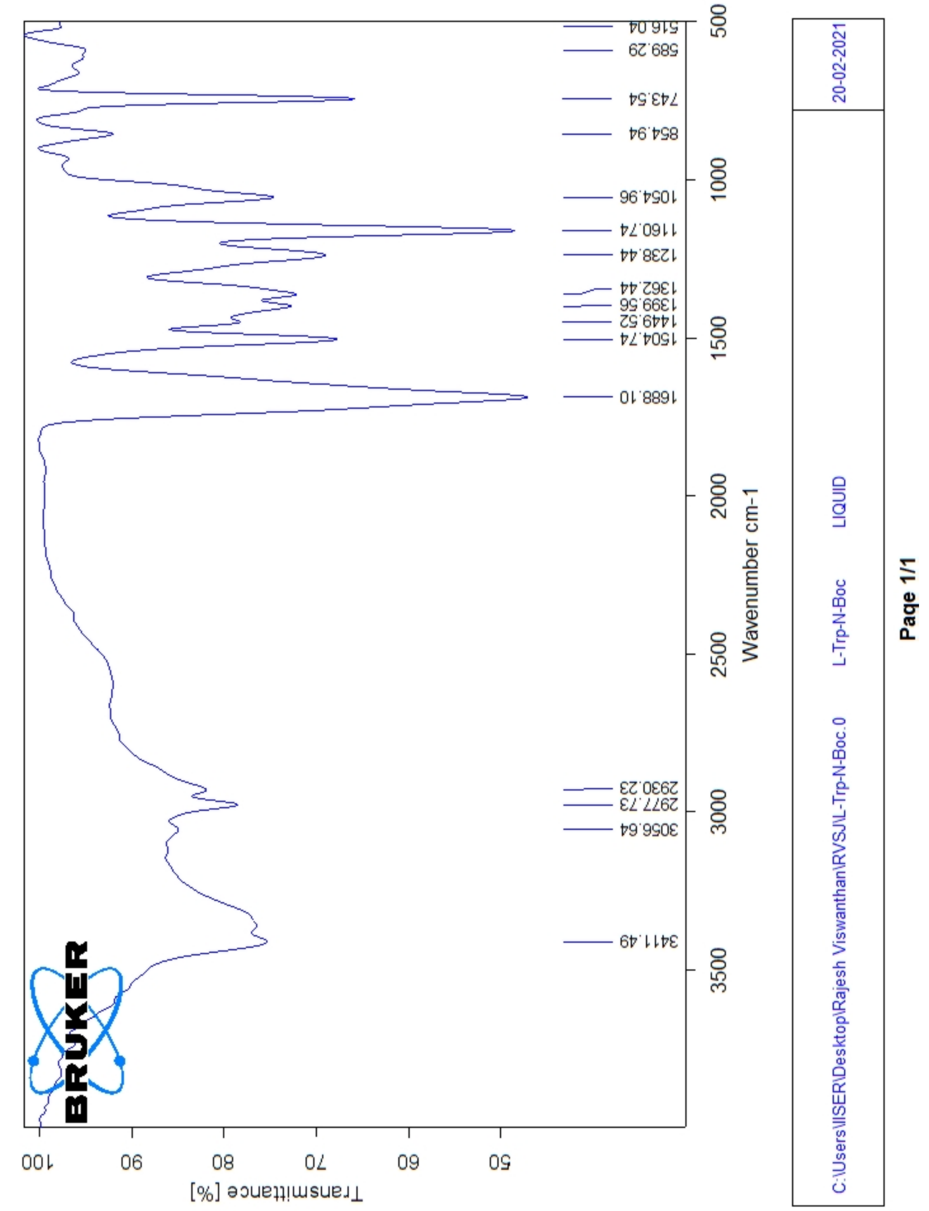

Supplementary Figure 24. Infrared spectrum for L-Trp-N-Boc.

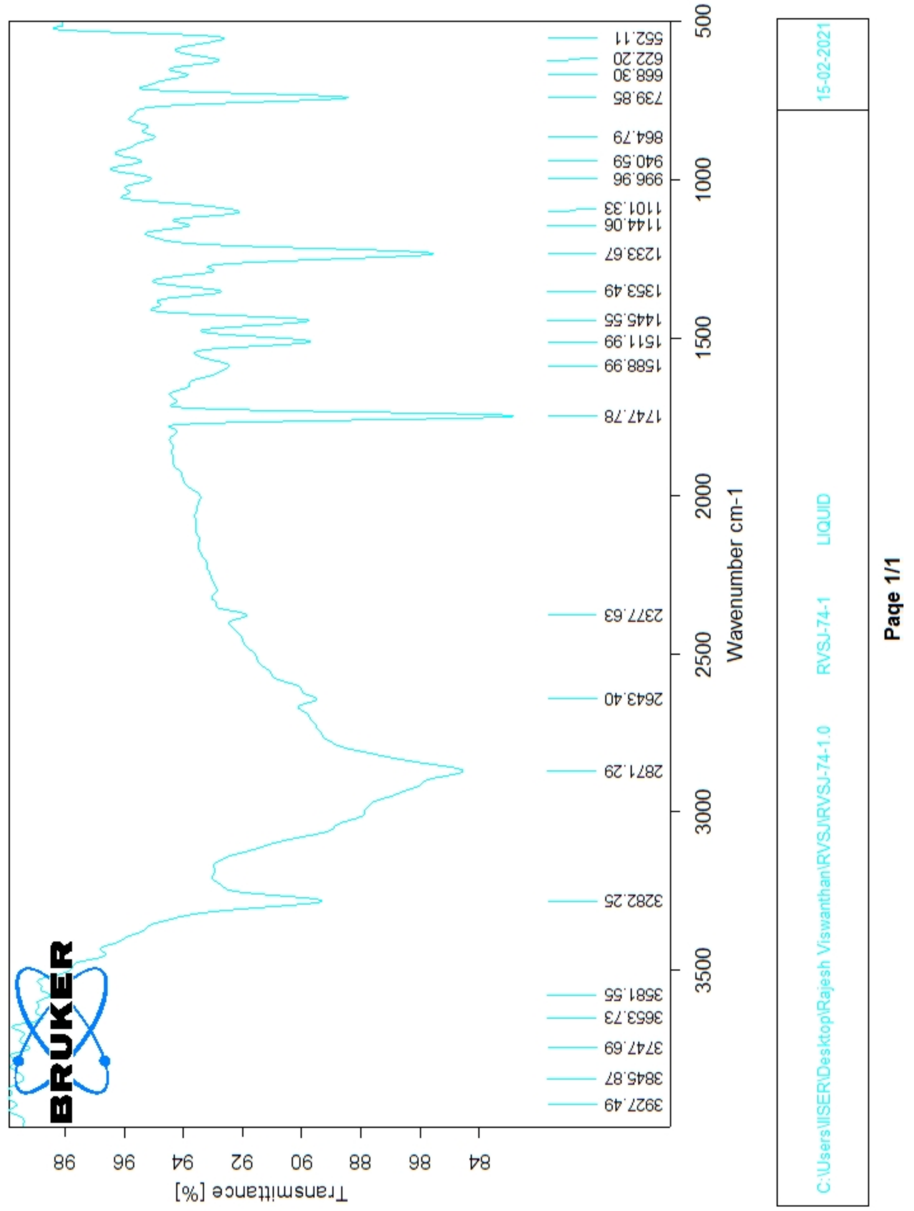

Supplementary Figure 25. Infrared spectrum for D-Trp-OMe hydrochloride salt.

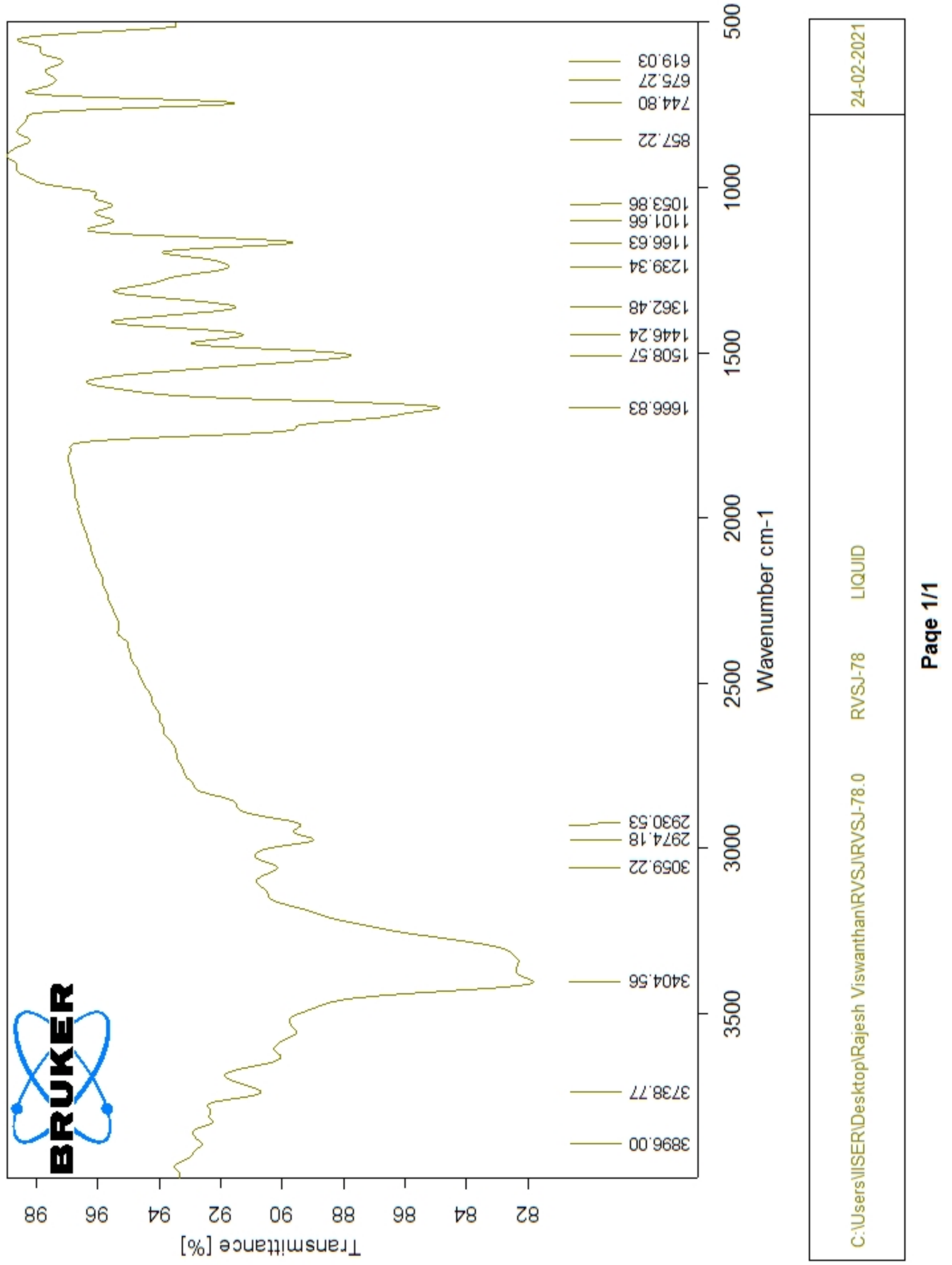

Supplementary Figure 26. Infrared spectrum for L-Trp-D-Trp dipeptide.

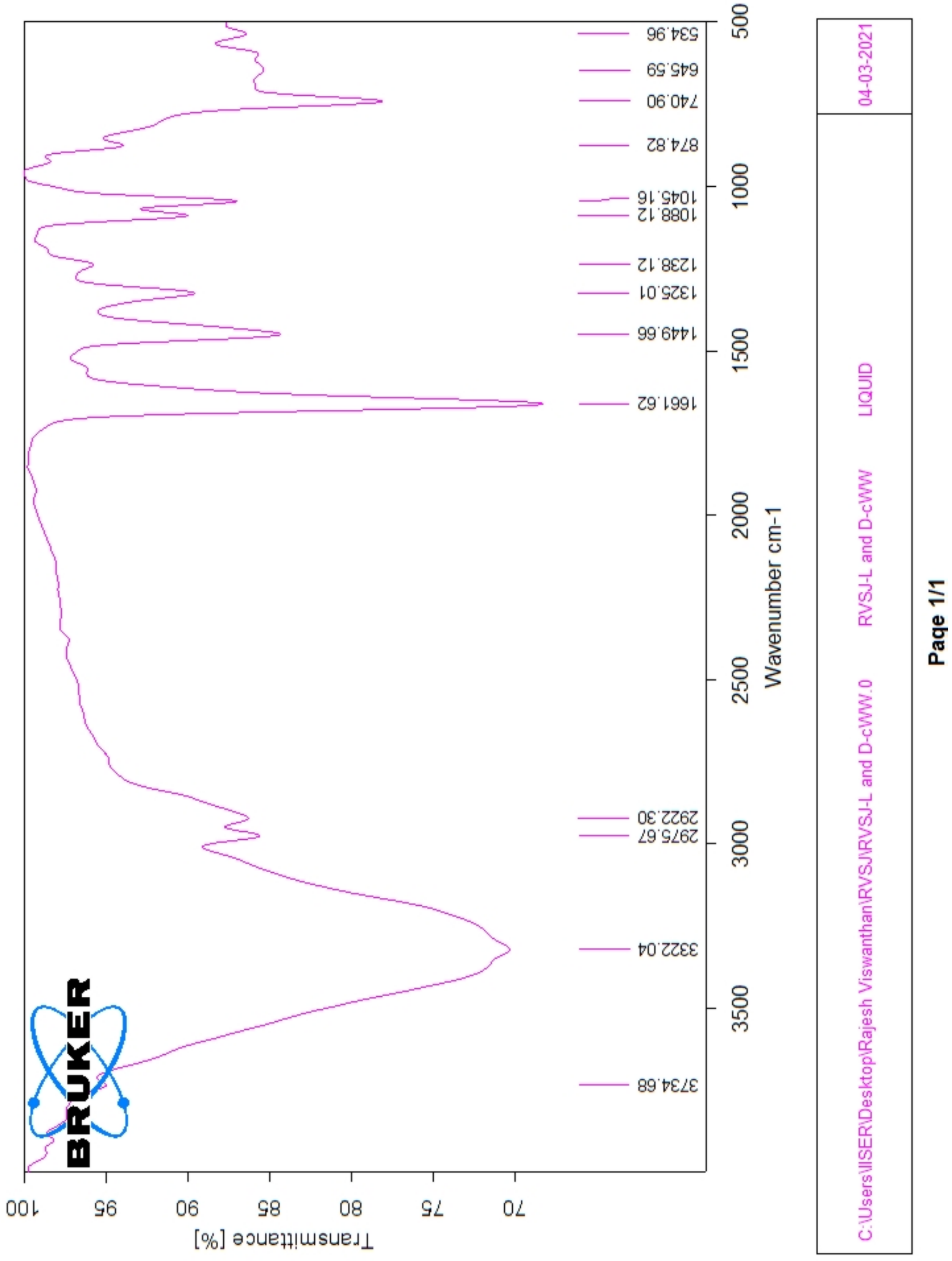

Supplementary Figure 27. Infrared spectrum for *cyclo*-L-Trp-D-Trp DKP (**4**).

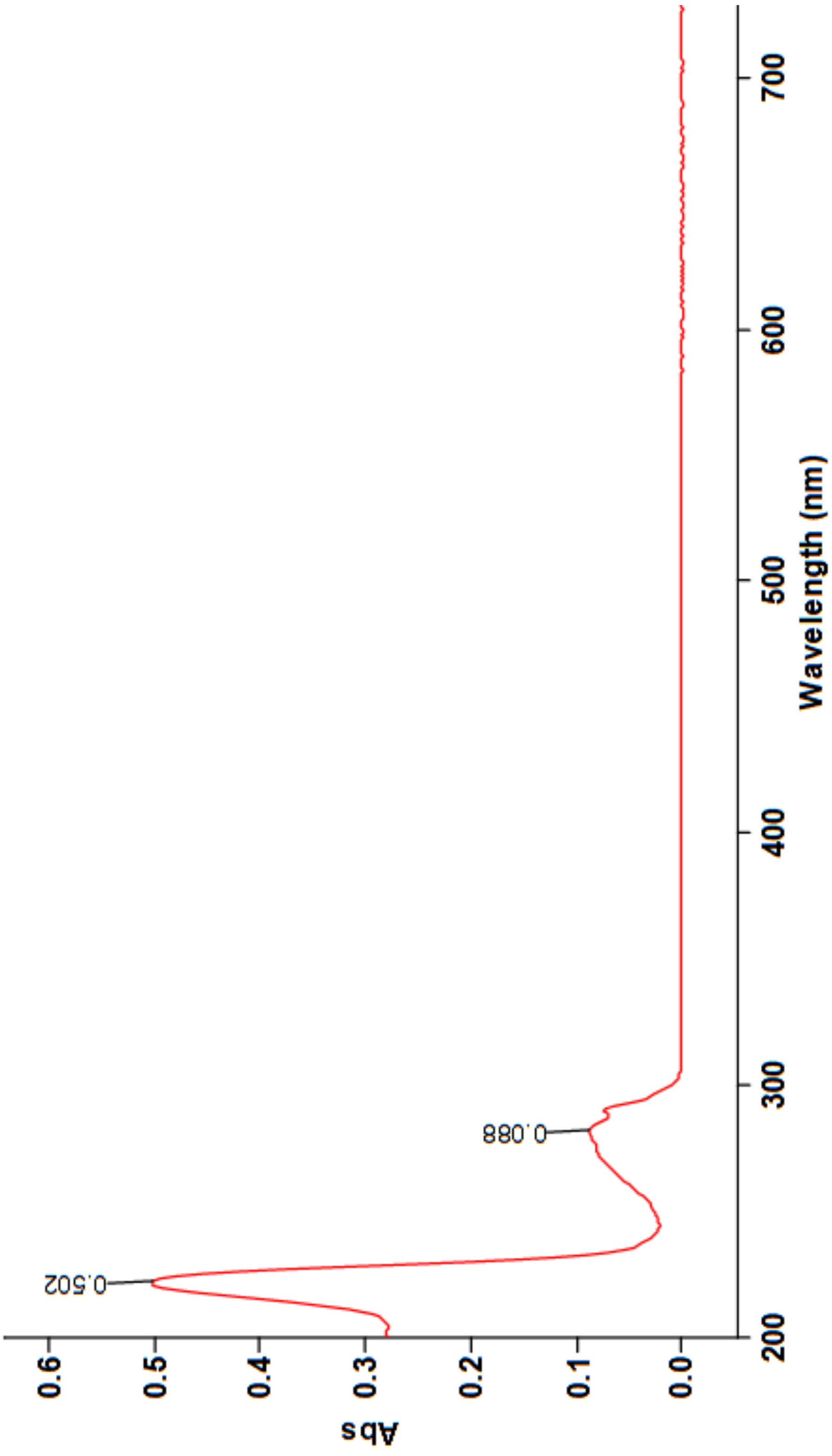

Supplementary Figure 28. UV-Vis spectrum for L-Trp-N-Boc.

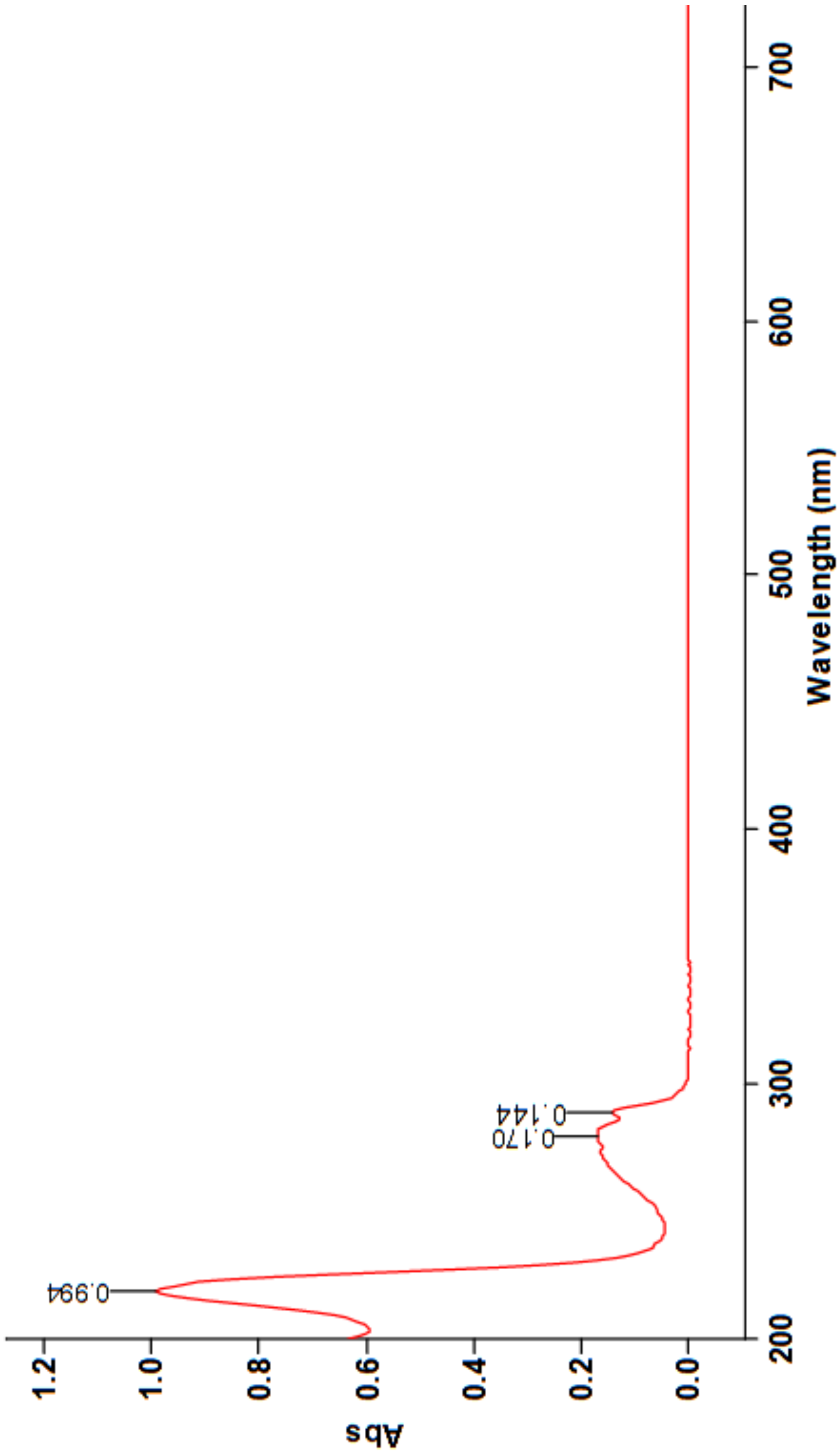

Supplementary Figure 29. UV-Vis spectrum for D-Trp-OMe hydrochloride salt.

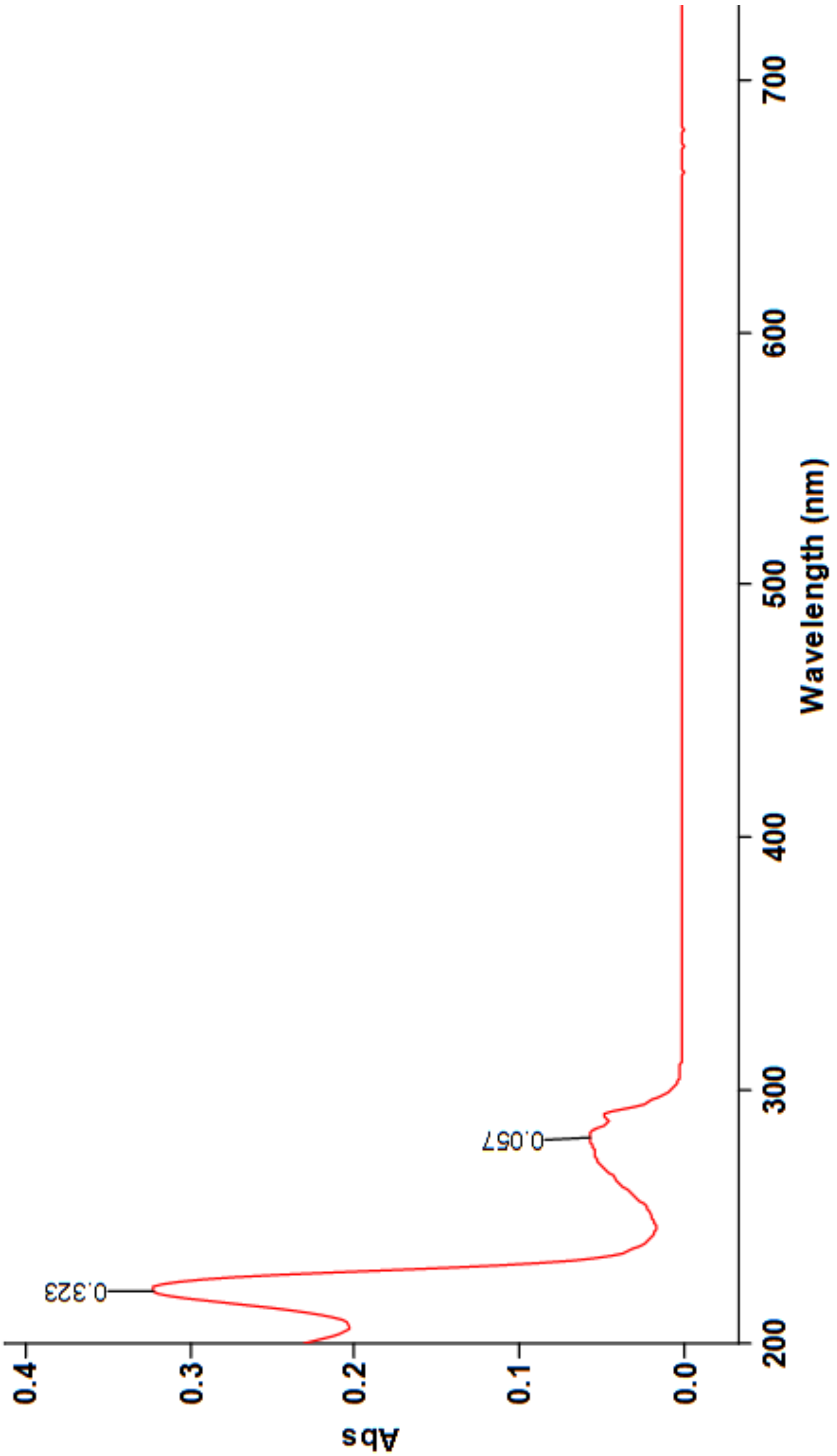

Supplementary Figure 30. UV-Vis spectrum for the L-Trp-D-Trp dipeptide.

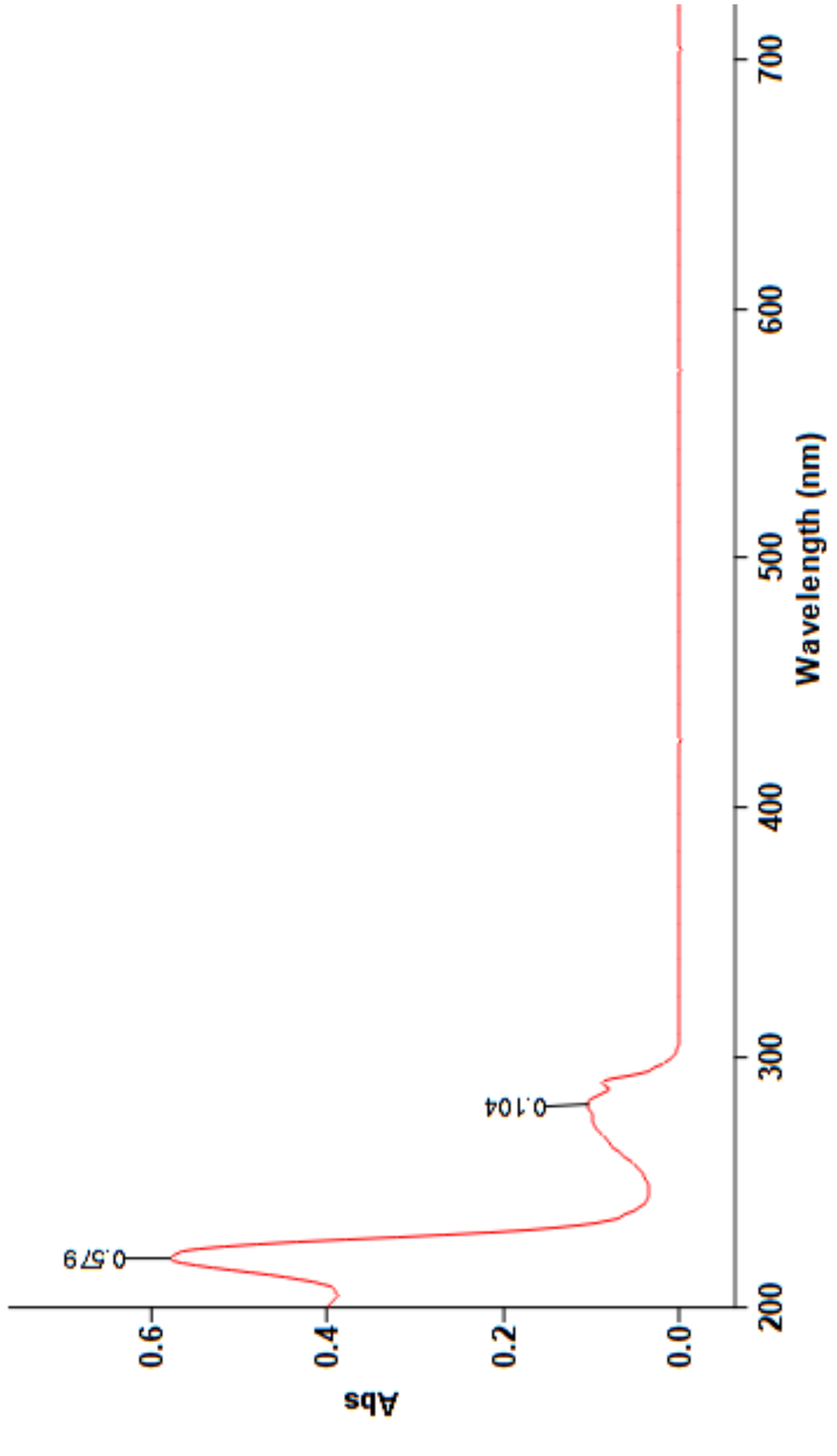

Supplementary Figure 31. UV-Vis spectrum for *cyclo*-L-Trp-D-Trp DKP (**4**).

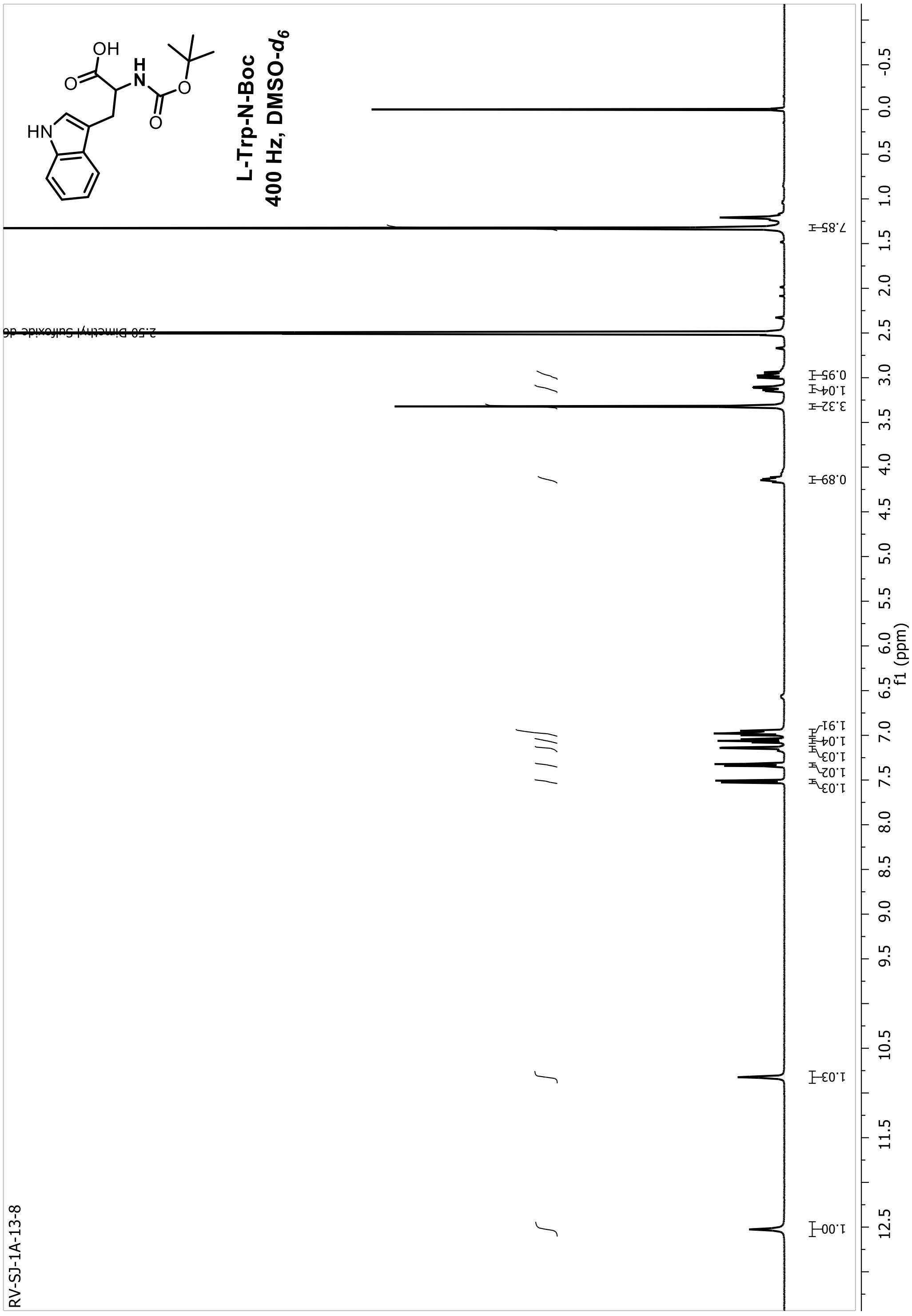

Supplementary Figure 32. ^{1}H NMR spectrum for L-Trp-N-Boc. Source data are provided as a Source Data file.

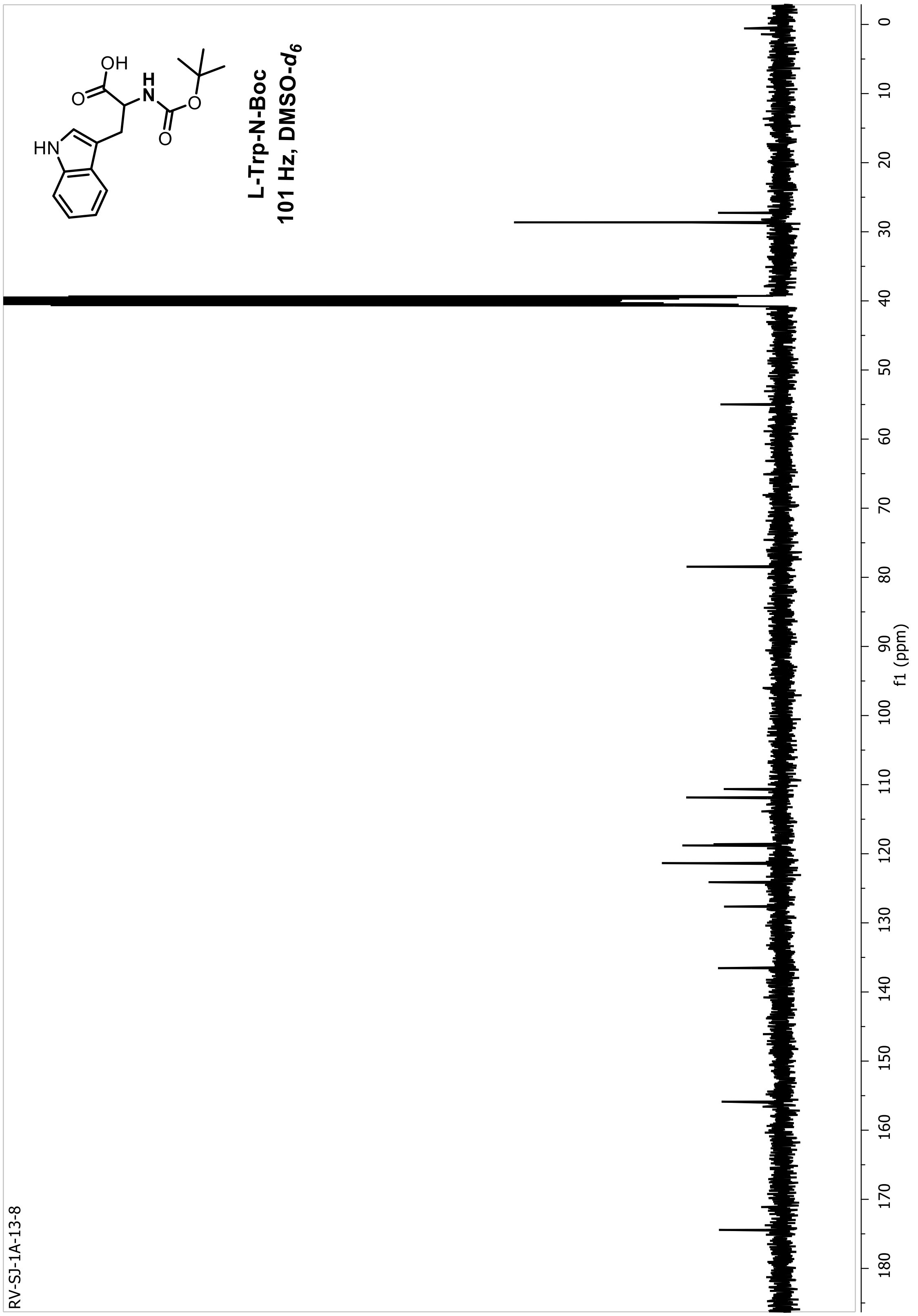

Supplementary Figure 33. ^{13}C NMR spectrum for L-Trp-N-Boc. Source data are provided as a Source Data file.

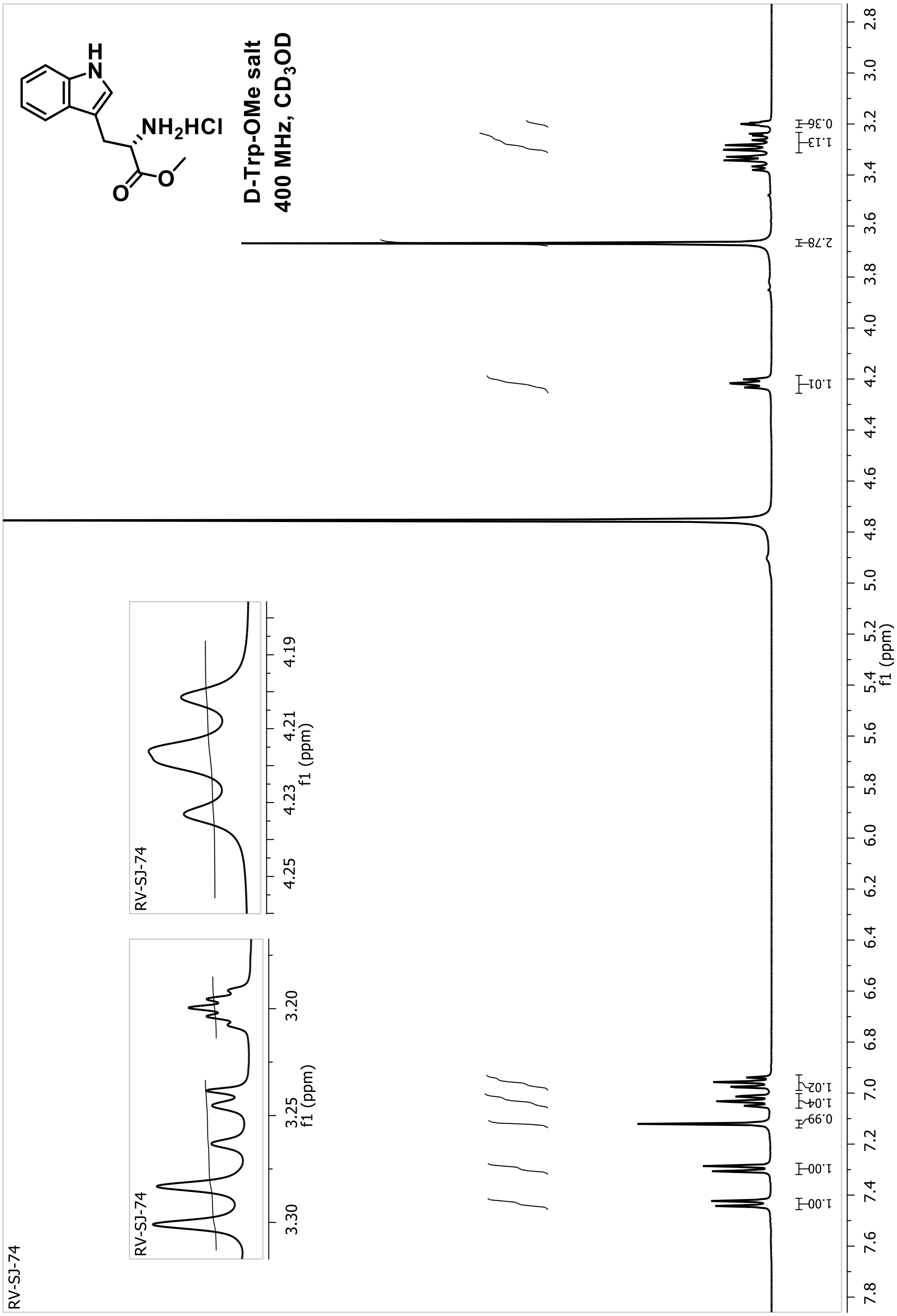

Supplementary Figure 34. ^{1}H NMR spectrum for D-Trp-O-Me hydrochloride salt. Source data are provided as a Source Data file.

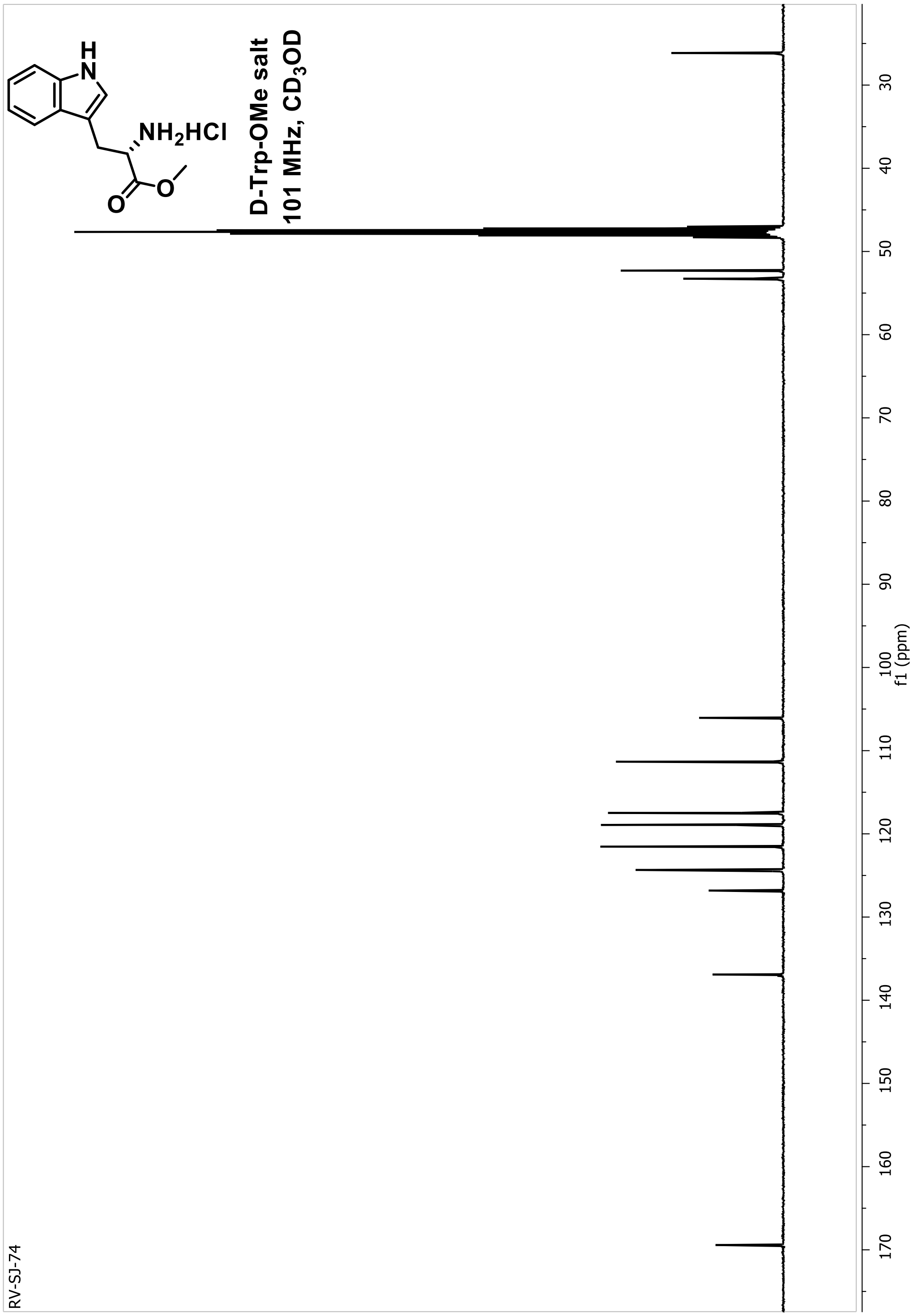

Supplementary Figure 35. ^{13}C NMR spectrum for D-Trp-O-Me hydrochloride salt. Source data are provided as a Source Data file. Source data are provided as a Source Data file.

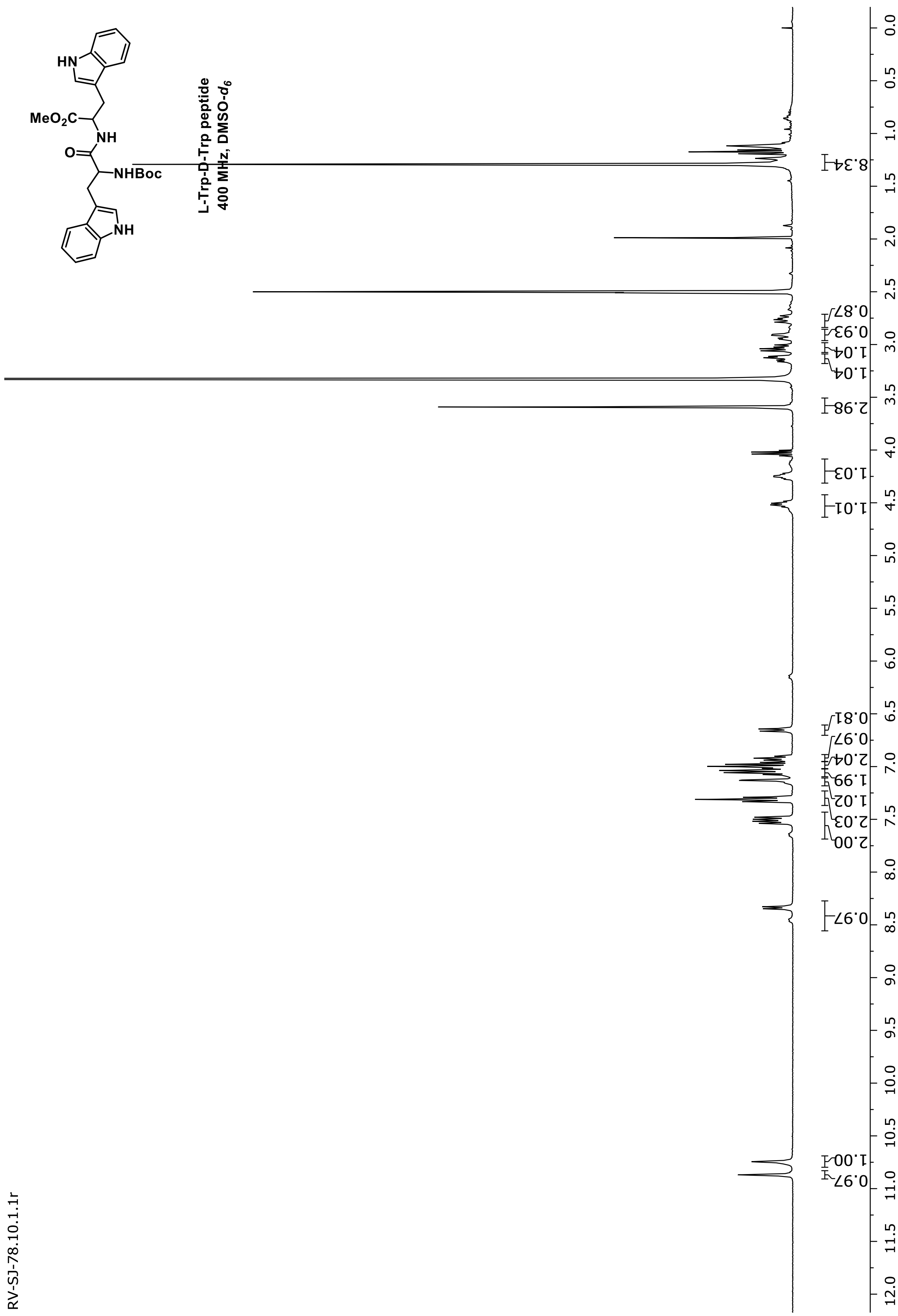

Supplementary Figure 36. ^{1}H NMR spectrum for L-Trp-D-Trp dipeptide. Source data are provided as a Source Data file.

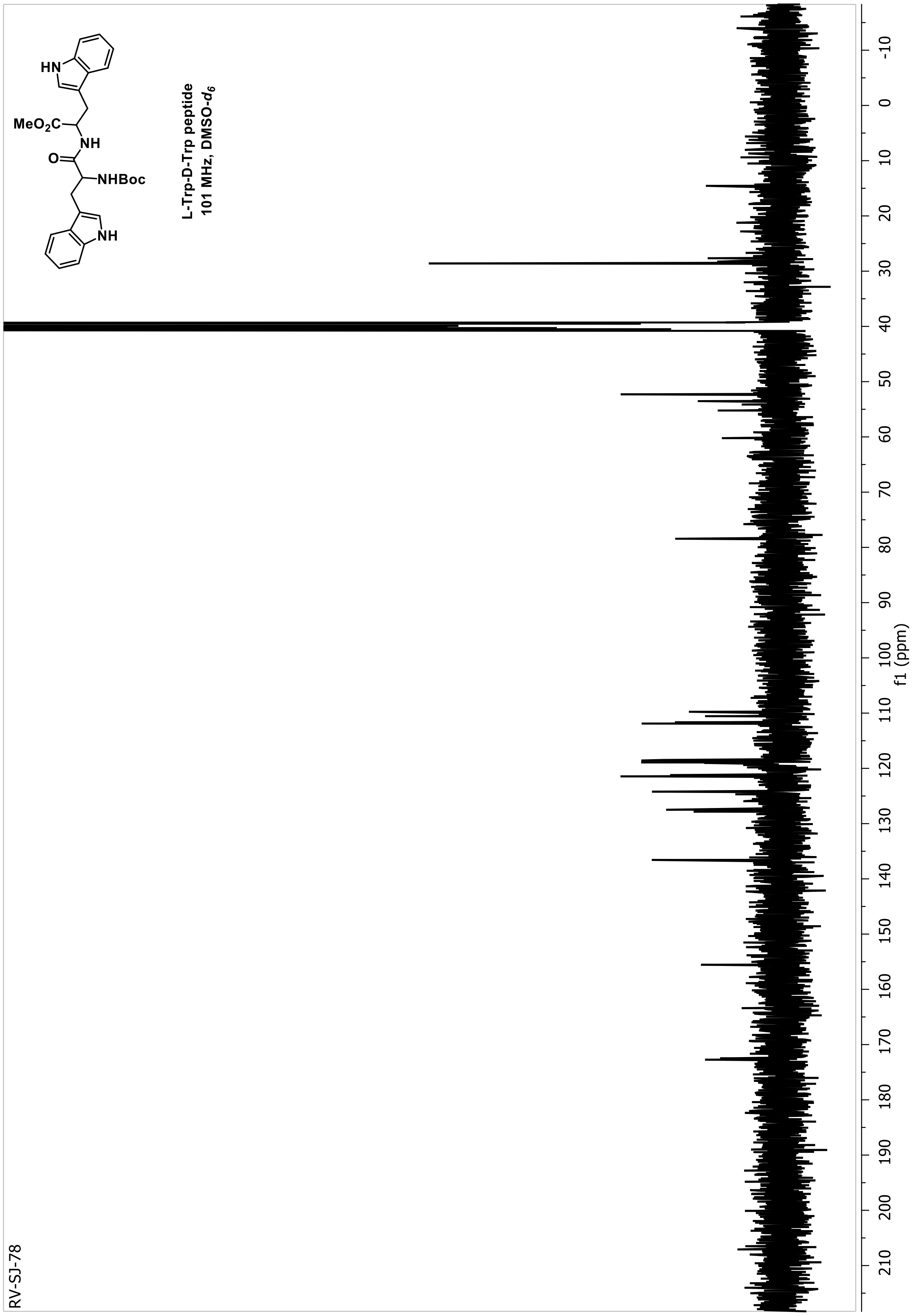

Supplementary Figure 37. ^{13}C NMR spectrum for L-Trp-D-Trp dipeptide. Source data are provided as a Source Data file.

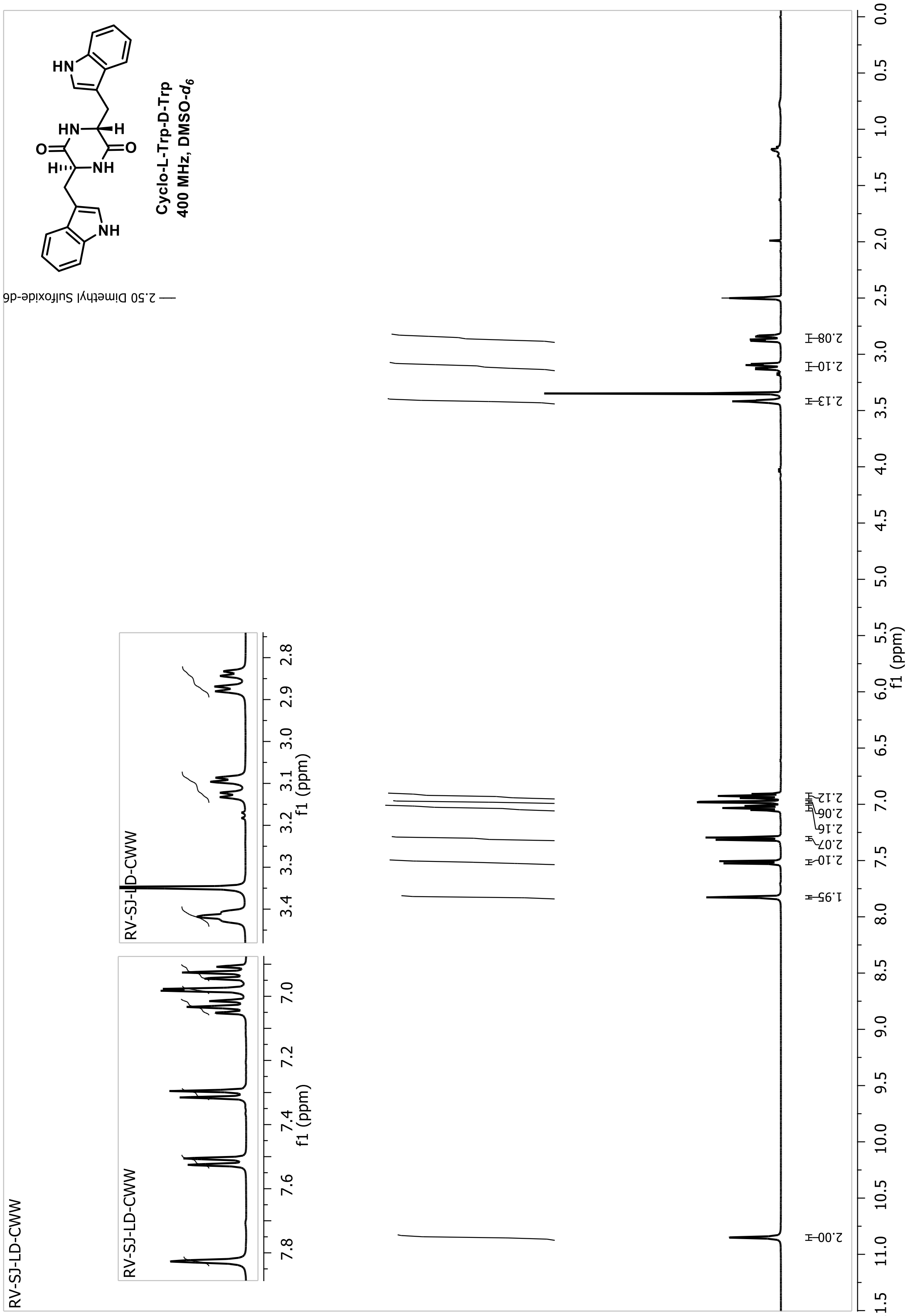

Supplementary Figure 38. ^{1}H NMR for synthetic *cyclo*-L-Trp-D-Trp DKP (**4**). Source data are provided as a Source Data file.

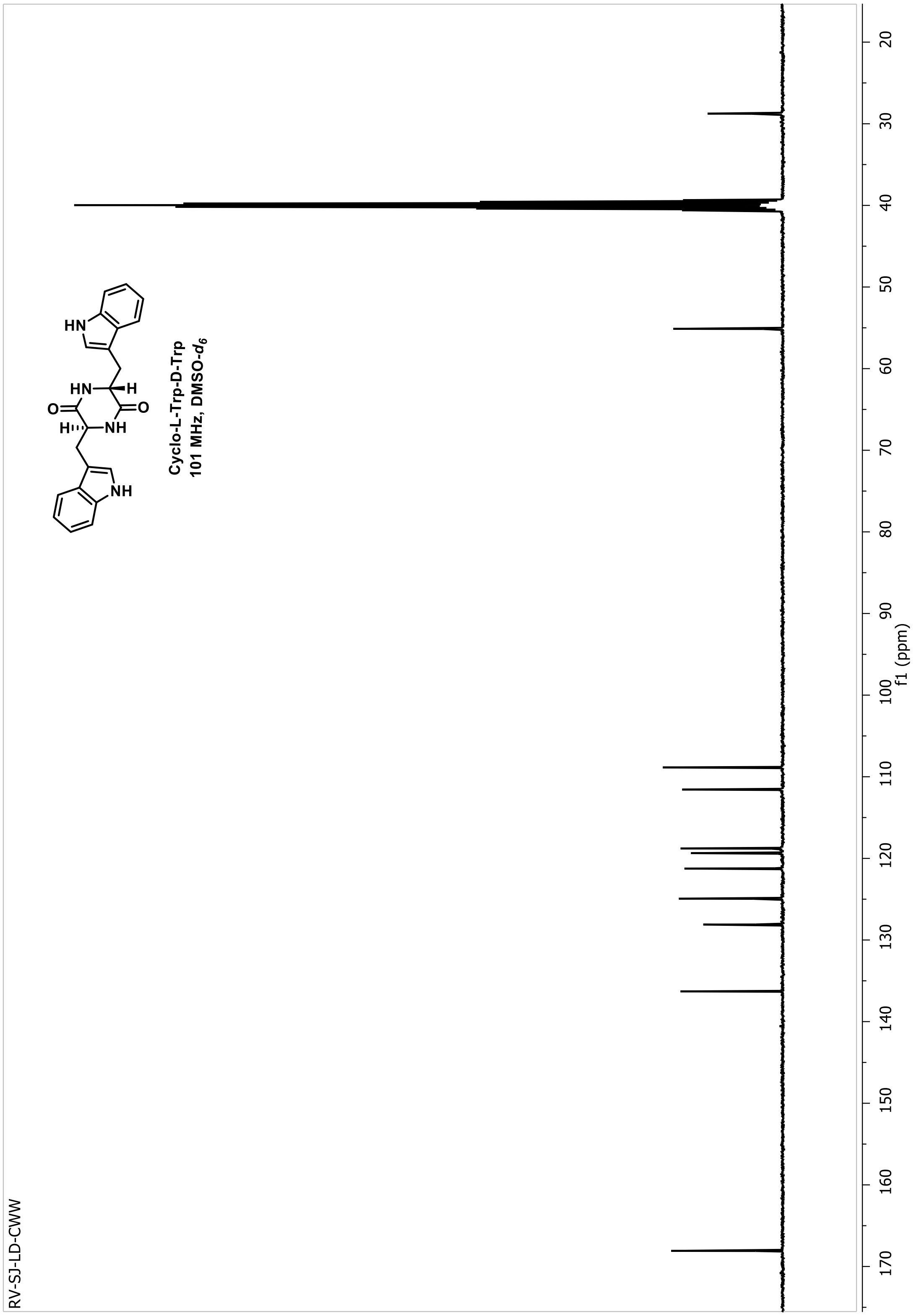

Supplementary Figure 39. ^{13}C NMR spectrum for synthetic cyclo-L-Trp-D-Trp DKP (**4**). Source data are provided as a Source Data file.

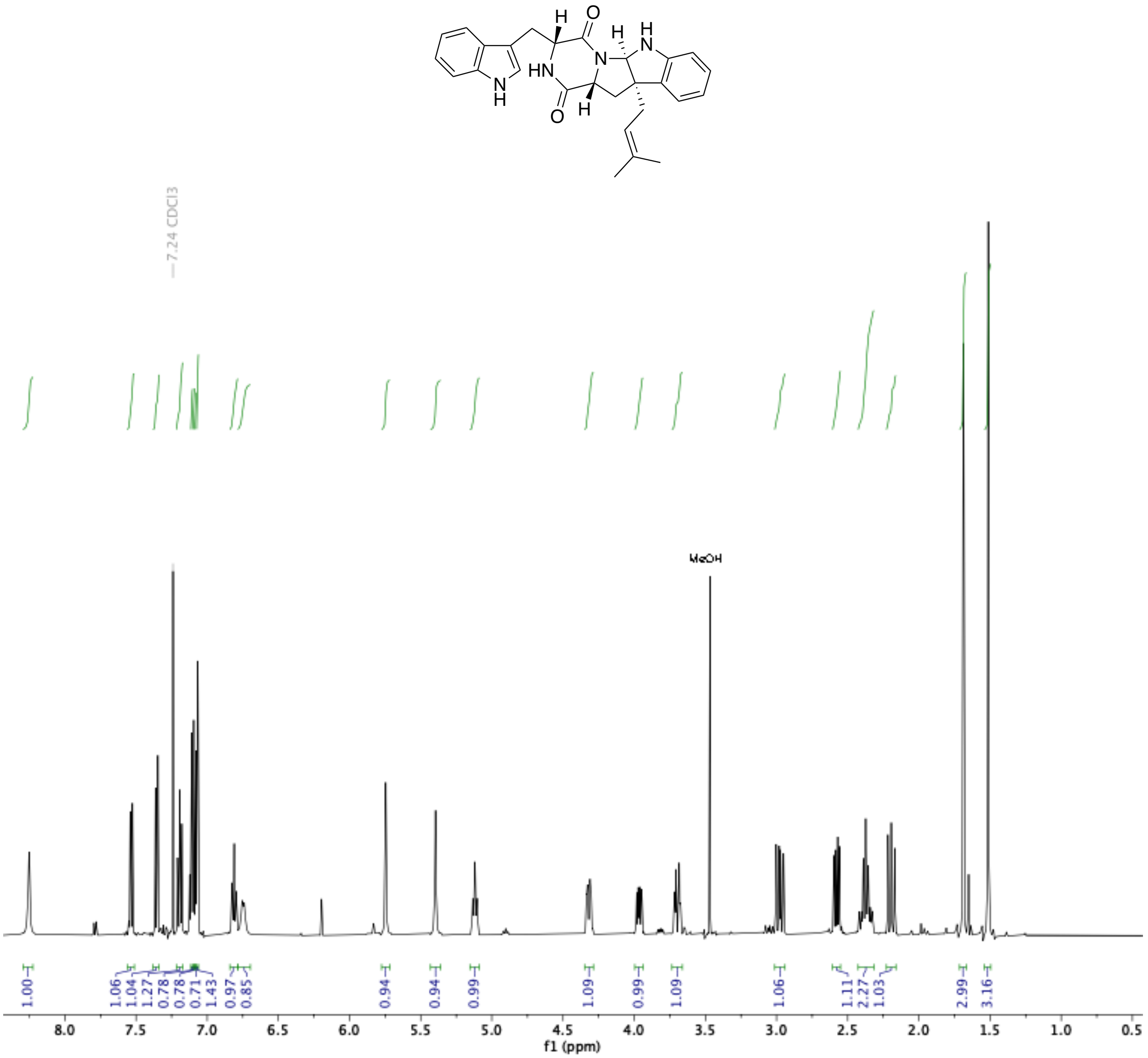

Supplementary Figure 40. ^{1}H NMR spectrum of synthetic *cyclo*-D-Trp-C3'-prenyl-D-Trp (**6**) (500 MHz, $CDCl_3$). Source data are provided as a Source Data file.

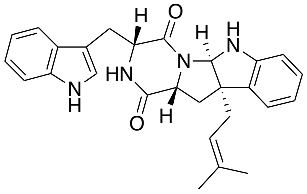

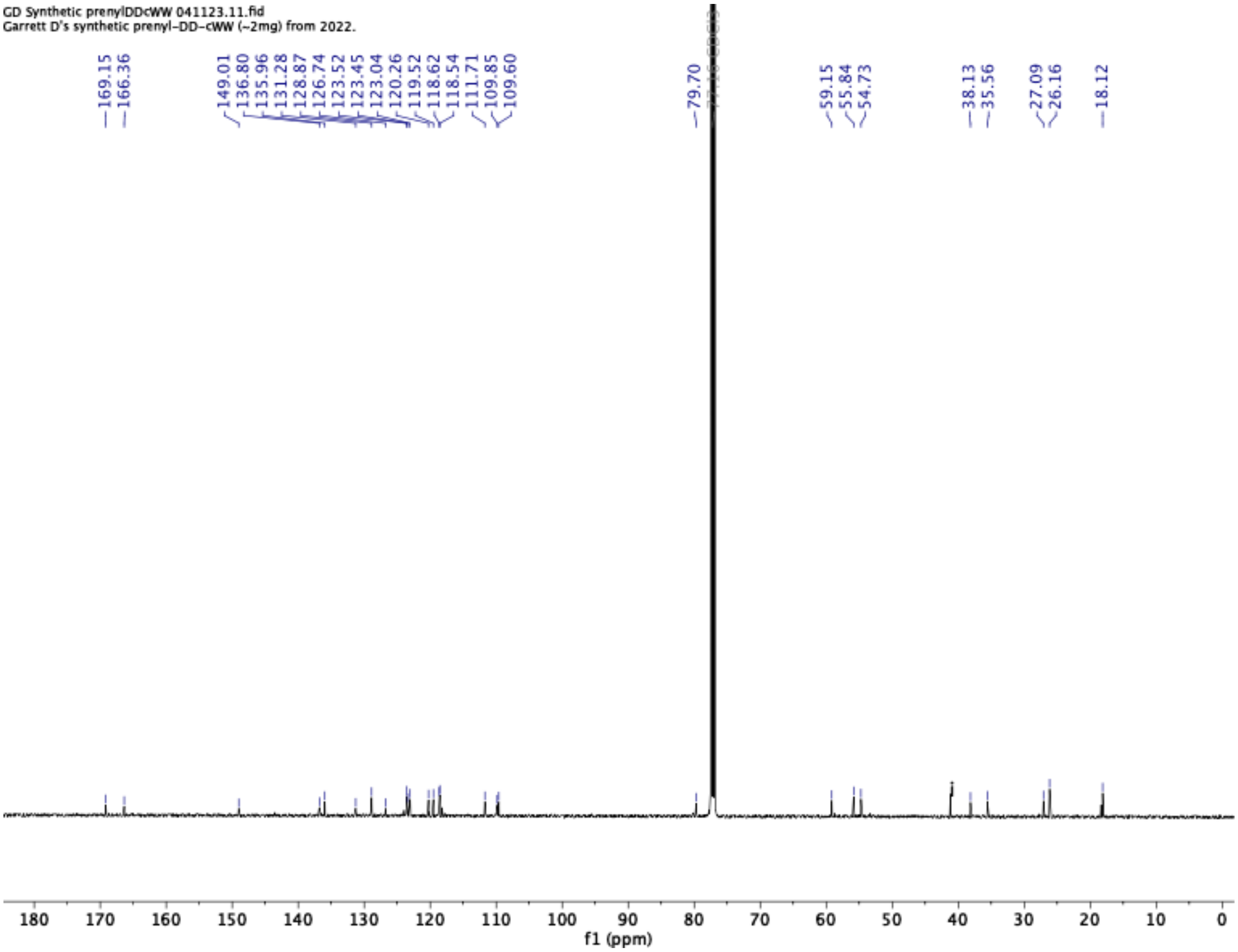

Supplementary Figure 41. ^{13}C NMR spectrum of synthetic *cyclo*-D-Trp-C3'-prenyl-D-Trp (**6**) (125 MHz, $CDCl_3$). Source data are provided as a Source Data file.

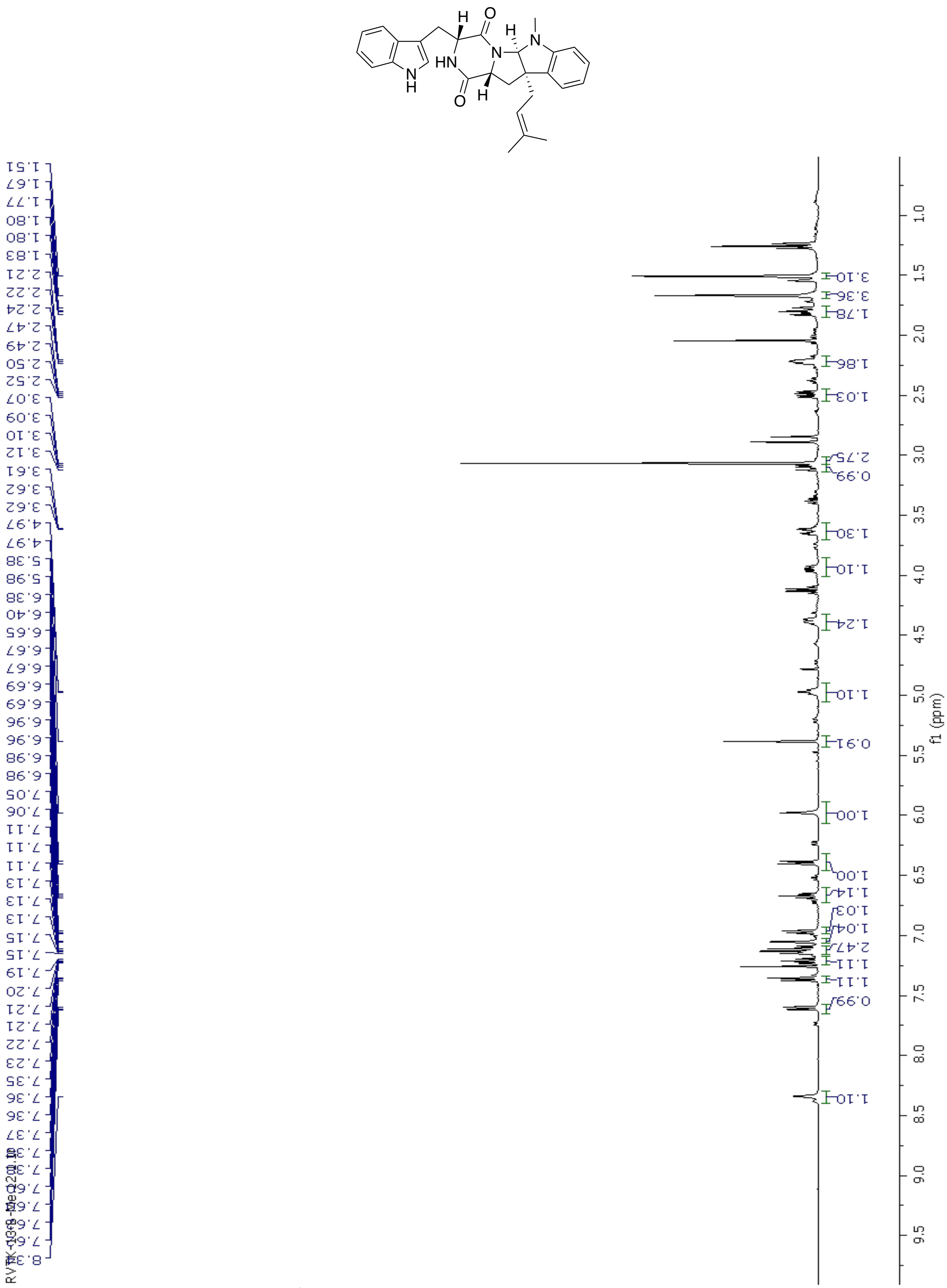

Supplementary Figure 42. ^{1}H NMR spectrum for synthetic *cyclo*-D-Trp-N1’-methyl-C3’-prenyl-D-Trp (**7**) (400 MHz, $CDCl_3$). Source data are provided as a Source Data file.

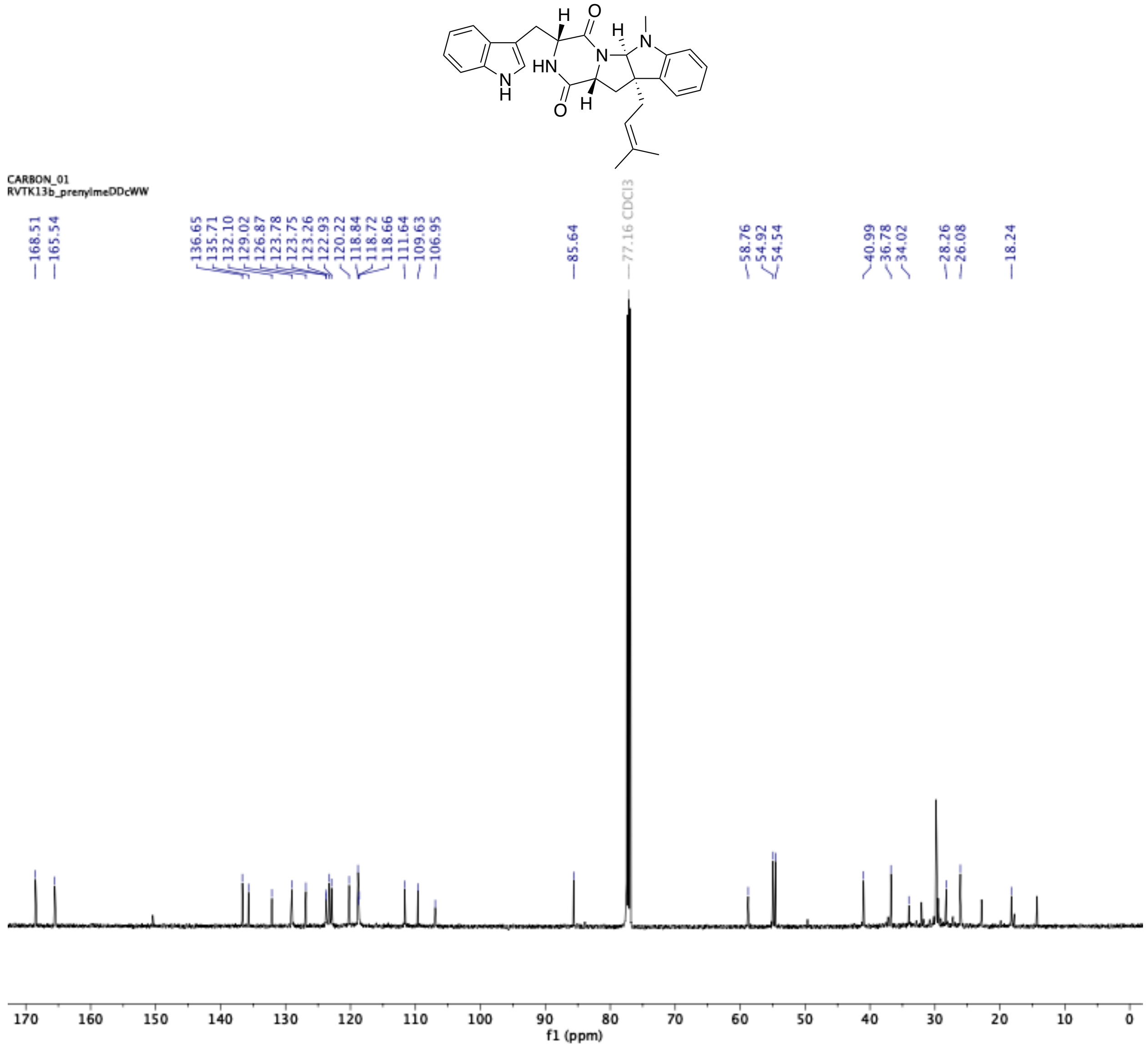

Supplementary Figure 43. ^{13}C NMR spectrum for synthetic *cyclo*-D-Trp-N1'-methyl-C3'-prenyl-D-Trp DKP (**7**) (125 MHz, $CDCl_3$). Source data are provided as a Source Data file.

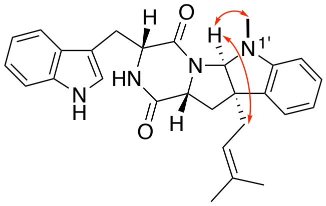

cyclo-D-Trp-N1'-methyl-C3'-prenyl-D-Trp (**7**)

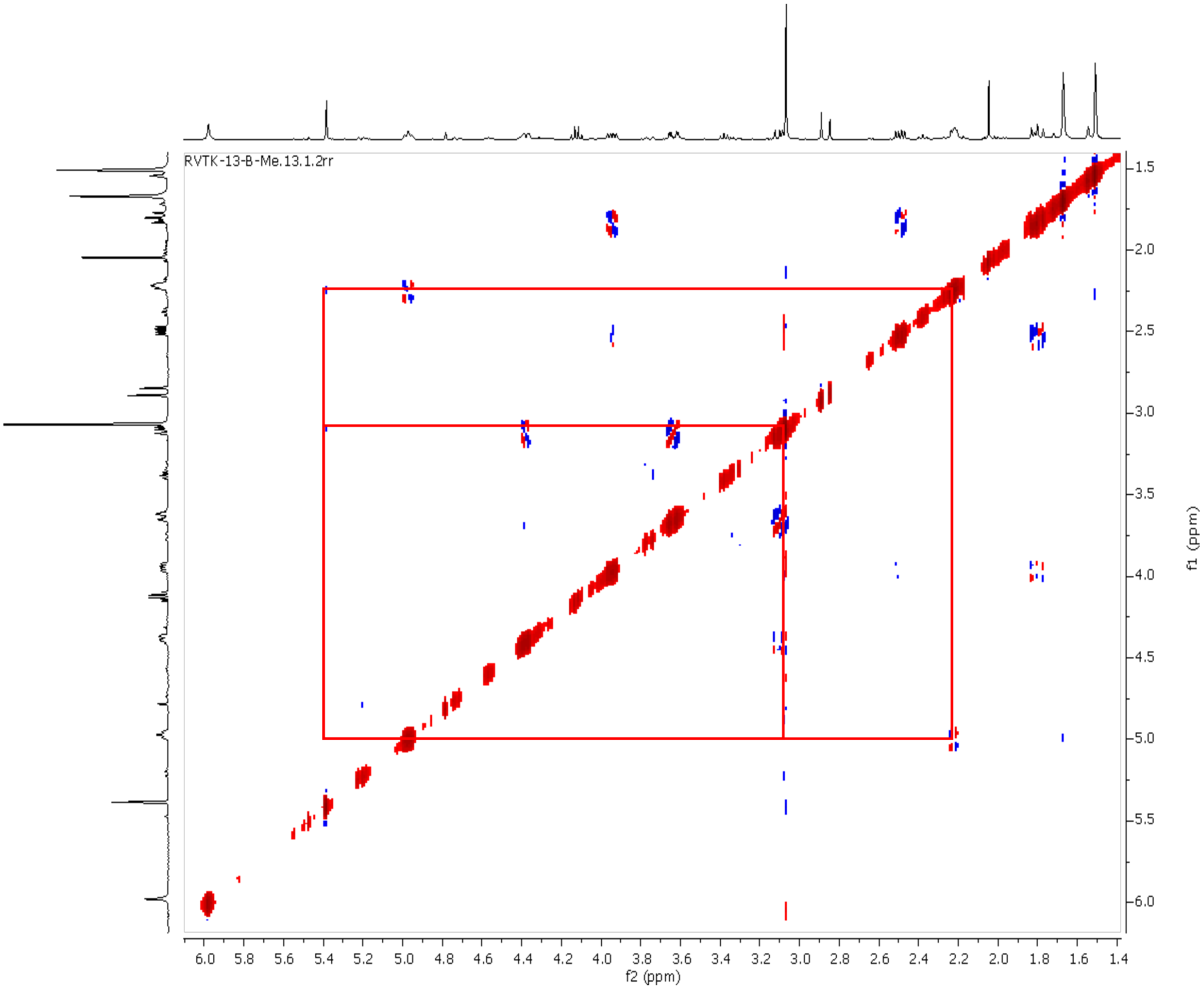

Supplementary Figure 44. ROESY spectrum for synthetic *cyclo*-D-Trp-N1'-methyl-C3'-prenyl-D-Trp (**7**) in $CDCl_3$. Red arrows on the structure denote key correlations observed; these are denoted by red lines in the spectrum. Source data are provided as a Source Data file.

References

1 Khopade, T. M., Ajayan, K., Joshi, S. S., Lane, A. L. & Viswanathan, R. Bioinspired Brønsted Acid-Promoted Regioselective Tryptophan Isoprenylations. *ACS Omega* **6**, 10840–10858 (2021).

2 Cendron, L. *et al.* The Structure and Function of a Microbial Allantoin Racemase Reveal the Origin and Conservation of a Catalytic Mechanism. *Biochemistry* **55**, 6421-6432 (2016).

3 French, J. B., Neau, D. B. & Ealick, S. E. Characterization of the structure and function of Klebsiella pneumoniae allantoin racemase. *J Mol Biol* **410**, 447-460 (2011).

4 Carney, A. E. & Holden, H. M. Molecular architecture of TylM1 from Streptomyces fradiae: an N,N-dimethyltransferase involved in the production of dTDP-D-mycaminose. *Biochemistry* **50**, 780-787 (2011).

5 Fick, R. J. *et al.* Structural and Functional Characterization of Sulfonium Carbon-Oxygen Hydrogen Bonding in the Deoxyamino Sugar Methyltransferase TylM1. *Biochemistry* **58**, 2152-2159 (2019).

6 Trott, O. & Olson, A. J. AutoDock Vina: improving the speed and accuracy of docking with a new scoring function, efficient optimization, and multithreading. *J Comput Chem* **31**, 455-461 (2010).

7 Alqahtani, N. *et al.* Synergism between Genome Sequencing, Tandem Mass Spectrometry and Bio-Inspired Synthesis Reveals Insights into Nocardioazine B Biogenesis. *Org. Biomol. Chem.* **13**, 7177-7192 (2015).

8 James, E. D. *et al.* Two Distinct Cyclodipeptide Synthases from a Marine Actinomycete Catalyze Biosynthesis of the Same Diketopiperazine Natural Product. *ACS Synth. Biol.* **5**, 547-553 (2016).
