# Supplementary material for: Unveiling an indole alkaloid diketopiperazine biosynthetic pathway that features a unique stereoisomerase and multifunctional methyltransferase
Source: Nat Commun. 2023 May 3;14:2558. doi: 10.1038/s41467-023-38168-3 (PMC10156859; doi:10.1038/s41467-023-38168-3)
Supplement: Supplementary file 2 — Description of Additional Supplementary Files [file 41467_2023_38168_MOESM2_ESM.pdf]

Description of Additional Supplementary Files

Supplementary Data 1: PDB file of NozR model docked with 3.

Supplementary Data 2: PDB file of NozMT model docked with 6.

Supplementary Data 3: PDB file of NozMT model docked with 5.
