# Supplementary material for: Unveiling an indole alkaloid diketopiperazine biosynthetic pathway that features a unique stereoisomerase and multifunctional methyltransferase
Source: Nat Commun. 2023 May 3;14:2558. doi: 10.1038/s41467-023-38168-3 (PMC10156859; doi:10.1038/s41467-023-38168-3)
Supplement: Supplementary file 6 — Reporting Summary [file 41467_2023_38168_MOESM6_ESM.pdf]

nature portfolio

Corresponding author(s): Amy L. Lane, Rajesh Viswanathan

Last updated by author(s): 03/22/2023

Reporting Summary

Nature Portfolio wishes to improve the reproducibility of the work that we publish. This form provides structure for consistency and transparency in reporting. For further information on Nature Portfolio policies, see our Editorial Policies and the Editorial Policy Checklist.

Statistics

For all statistical analyses, confirm that the following items are present in the figure legend, table legend, main text, or Methods section.

n/a	Confirmed	
☐	☒	The exact sample size (n) for each experimental group/condition, given as a discrete number and unit of measurement
☐	☒	A statement on whether measurements were taken from distinct samples or whether the same sample was measured repeatedly
☒	☐	The statistical test(s) used AND whether they are one- or two-sided<br>*Only common tests should be described solely by name; describe more complex techniques in the Methods section.*
☒	☐	A description of all covariates tested
☒	☐	A description of any assumptions or corrections, such as tests of normality and adjustment for multiple comparisons
☒	☐	A full description of the statistical parameters including central tendency (e.g. means) or other basic estimates (e.g. regression coefficient) AND variation (e.g. standard deviation) or associated estimates of uncertainty (e.g. confidence intervals)
☒	☐	For null hypothesis testing, the test statistic (e.g. F, t, r) with confidence intervals, effect sizes, degrees of freedom and P value noted<br>*Give P values as exact values whenever suitable.*
☒	☐	For Bayesian analysis, information on the choice of priors and Markov chain Monte Carlo settings
☒	☐	For hierarchical and complex designs, identification of the appropriate level for tests and full reporting of outcomes
☒	☐	Estimates of effect sizes (e.g. Cohen's d, Pearson's r), indicating how they were calculated

Our web collection on statistics for biologists contains articles on many of the points above.

Software and code

Policy information about availability of computer code

Data collection	Molecular models and docking data were collected using PHYRE v2.0, Prime v3.0 (Schrodinger, LLC), YASARA v21.8.26 SSP/Homology/PSSM, AutoDock4, Sybyl v8.0, and Pymol v2.0. References for academic programs are provided in manuscript and/or Supplementary Information. Bioinformatics analyses and protein sequence alignments were conducted using GeneMarkS version 4.28, Geneious v10, and ClustalW v1.2.2.
Data analysis	Raw LC-MS data were evaluated using Thermo XCalibur and CSV files exported to GraphPad Prism 9 for plotting. NMR data were processed and evaluated using MestReNova v14 (MestReLab). HPLC-UV data was processed and evaluated using Agilent OpenLab v2.7. Kinetics plots were prepared using GraphPad Prism 9 with Michaelis Menten kinetics non-linear regression package.

For manuscripts utilizing custom algorithms or software that are central to the research but not yet described in published literature, software must be made available to editors and reviewers. We strongly encourage code deposition in a community repository (e.g. GitHub). See the Nature Portfolio guidelines for submitting code & software for further information.

Data

Policy information about availability of data

All manuscripts must include a data availability statement. This statement should provide the following information, where applicable:

- Accession codes, unique identifiers, or web links for publicly available datasets
- A description of any restrictions on data availability
- For clinical datasets or third party data, please ensure that the statement adheres to our policy

The noz2 cluster has been deposited in the GenBank database under accession code MZ913435 [https://www.ncbi.nlm.nih.gov/nuccore/MZ913435]. Other

sequences from this study are available under the following accession codes: noz cluster KT184400 [https://www.ncbi.nlm.nih.gov/nuccore/KT184400]; ncd cluster KT184401 [https://www.ncbi.nlm.nih.gov/nuccore/KT184401]; representative racemases 2XEC [https://www.ncbi.nlm.nih.gov/Structure/pdb/2XEC], 3UXK [https://www.ncbi.nlm.nih.gov/Structure/pdb/3uxk], AAQ93382 [https://www.ncbi.nlm.nih.gov/protein/AAQ93382], 5WXZ [https://www.ncbi.nlm.nih.gov/Structure/pdb/5WXZ], 1JFL [https://www.ncbi.nlm.nih.gov/Structure/pdb/1JFL], 5EVC [https://www.ncbi.nlm.nih.gov/Structure/pdb/5EVC], A0A140N890 [https://www.ncbi.nlm.nih.gov/protein/A0A140N890], 3OUT [https://www.ncbi.nlm.nih.gov/Structure/pdb/3OUT], 1B74 [https://www.ncbi.nlm.nih.gov/Structure/pdb/1B74], 2JFN [https://www.ncbi.nlm.nih.gov/Structure/pdb/2JFN], 4FQ7 [https://www.ncbi.nlm.nih.gov/Structure/pdb/4FQ7]; representative prenyltransferases ALL53320 [https://www.ncbi.nlm.nih.gov/protein/ALL53320], AVP32202 [https://www.ncbi.nlm.nih.gov/protein/AVP32202], CQR65853 [https://www.ncbi.nlm.nih.gov/protein/CQR65853], RLV09121 [https://www.ncbi.nlm.nih.gov/protein/RLV09121], RLV08932 [https://www.ncbi.nlm.nih.gov/protein/RLV08932]; representative methyltransferases QEI59523 [https://www.ncbi.nlm.nih.gov/protein/QEI59523], CQR65853 [https://www.ncbi.nlm.nih.gov/protein/CQR65853].

All data supporting the findings of this study are provided within the main text, Supplementary Information, and/or Supplementary Data. Raw data underlying figures are provided as a zipped Source Data file.

Human research participants

Policy information about studies involving human research participants and Sex and Gender in Research.

Reporting on sex and gender	N/A
Population characteristics	N/A
Recruitment	N/A
Ethics oversight	N/A

Note that full information on the approval of the study protocol must also be provided in the manuscript.

Field-specific reporting

Please select the one below that is the best fit for your research. If you are not sure, read the appropriate sections before making your selection.

[x] Life sciences [ ] Behavioural & social sciences [ ] Ecological, evolutionary & environmental sciences

For a reference copy of the document with all sections, see nature.com/documents/nr-reporting-summary-flat.pdf

Life sciences study design

All studies must disclose on these points even when the disclosure is negative.

Sample size	Sample sizes were selected based on literature precedents for n=2-3 independent replicates for the types of analyses conducted in this study (e.g. heterologous expression, biotransformations, in vitro enzyme assays). Literature precedents with n=1-3 replicates for this type include refs. 6-11, 14, and 18 from our manuscript.
Data exclusions	No data were excluded.
Replication	All constructs were created as at least n=2 independent replicates that were handled and tested independently throughout the course of the study (i.e. in vivo biotransformations, in vitro studies). All attempts at replication were successful.
Randomization	Treatment and control samples were selected in random order for analyses (e.g. enzyme assay setup order, incubator position, LC-MS queue).
Blinding	Blinding was not applicable toward this study, since all selected analytical techniques (e.g. LC-MS, ECD, NMR) are unbiased.

Reporting for specific materials, systems and methods

We require information from authors about some types of materials, experimental systems and methods used in many studies. Here, indicate whether each material, system or method listed is relevant to your study. If you are not sure if a list item applies to your research, read the appropriate section before selecting a response.

Materials & experimental systems

n/a	Involved in the study	
☒	☐	Antibodies
☒	☐	Eukaryotic cell lines
☒	☐	Palaeontology and archaeology
☒	☐	Animals and other organisms
☒	☐	Clinical data
☒	☐	Dual use research of concern

Methods

n/a	Involved in the study	
☒	☐	ChIP-seq
☒	☐	Flow cytometry
☒	☐	MRI-based neuroimaging
