# Supplementary material for: Unveiling an indole alkaloid diketopiperazine biosynthetic pathway that features a unique stereoisomerase and multifunctional methyltransferase
Source: Nat Commun. 2023 May 3;14:2558. doi: 10.1038/s41467-023-38168-3 (PMC10156859; doi:10.1038/s41467-023-38168-3)
Supplement: Supplementary file 7 — Source Data [file 41467_2023_38168_MOESM7_ESM.zip › Source Data/Synthetic 6 NMR/Synthetic6_gHSQC/HSQC_Synthetic6.pdf]

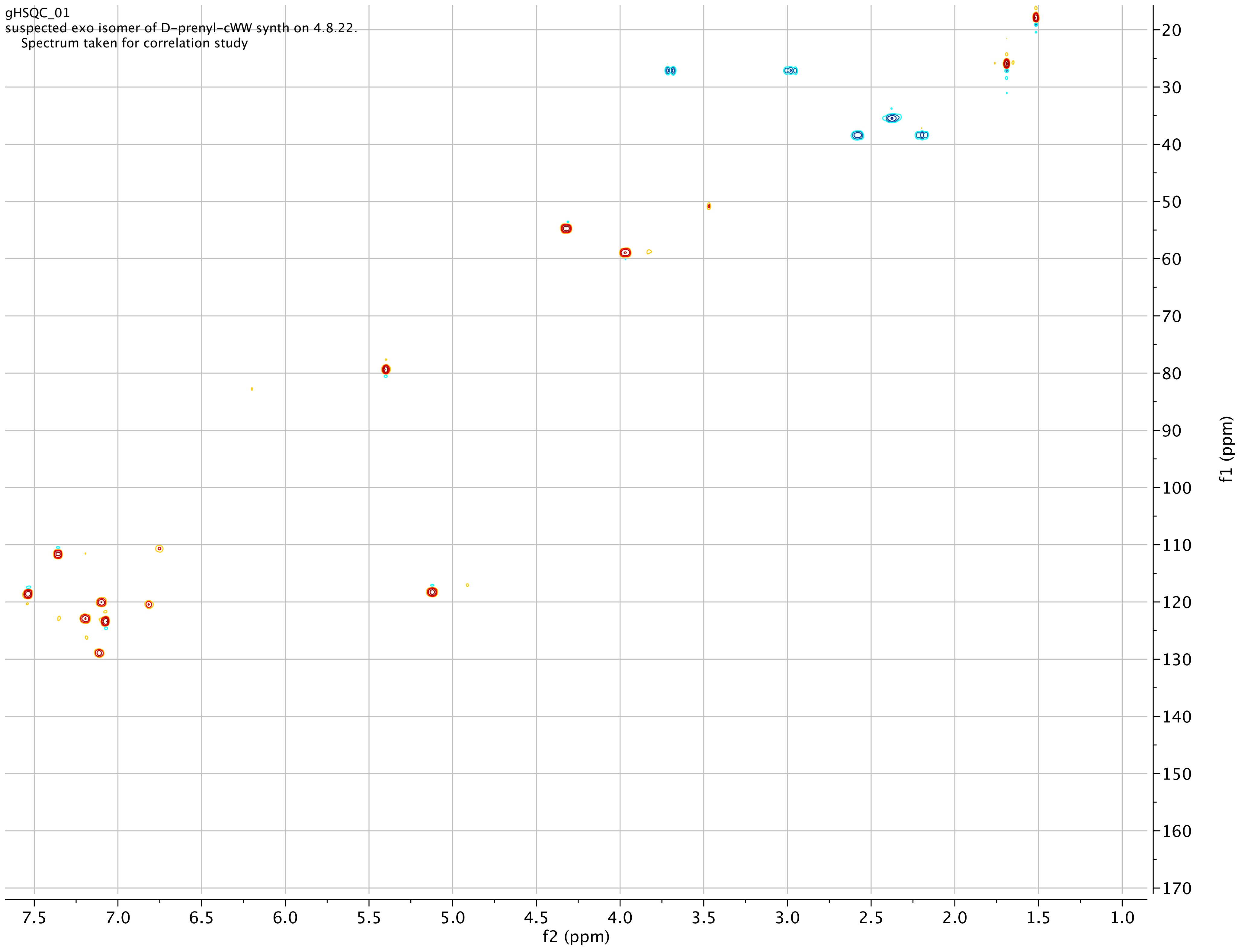
gHSQC_01
suspected exo isomer of D-prenyl-cWW synth on 4.8.22.
Spectrum taken for correlation study
7.5
7.0
6.5
6.0
5.5
5.0
4.5
4.0
3.5
3.0
2.5
2.0
1.5
1.0
f2 (ppm)
20
30
40
50
60
70
80
90
100
110
120
130
140
150
160
170
f1 (ppm)
